# Examining the indirect effects of life satisfaction and perceived social support on selection optimization compensation and PTSD among the senior citizens of Ekiti state: A moderated mediation approach

**DOI:** 10.1371/journal.pmen.0000186

**Published:** 2025-06-24

**Authors:** Dogbahgen Alphonso Yarseah, Omolayo Ogunsanmi Ololade, Ogunsanmi Joyce Olufunke, Alade Folasade Adesola, Falana Bernard Akinlabi, Ibimiluyi Francis Olu, Viola H. Cheeseman

**Affiliations:** 1 Faculty of Education, Department of Guidance and Counseling, Ekiti State University, Ekiti State, Nigeria; 2 Department of Public Health, Babcock University, Ogun State, Nigeria; 3 Department of Guidance and Counseling, Ekiti State University, Facuty of Education, Ekiti State, Nigeria; 4 Faculty of Education, Department of Guidance and Counseling, Ekiti State Universisty, Ekiti State, Nigeria; 5 Faculty of Education, Department of Guidance and Counseling, Ekiti State University, Ekiti State, Nigeria; 6 Faculty of Education, Department of Guidance and Counseling, Ekiti State University, Ado Ekiti, Nigeria; 7 Wuhan University, School of Cyber Science and Engineering, China; West China Hospital of Sichuan University, CHINA

## Abstract

This study addresses the escalating concern of Posttraumatic Stress Disorder (PTSD) among the elderly population in Ekiti State, Nigeria. As the country experiences a growing number of older individuals, understanding and mitigating PTSD in this demographic is of paramount importance. Despite this urgency, there is a significant gap in the literature pertaining to PTSD among older Nigerians. To fill this void, our research investigates the impact of Selection, Optimization, and Compensation (SOC) strategies on perceived social support (PSS), life satisfaction (LS), and PTSD in the elderly. Our study explores the relationships between PSS and LS, considering their potential roles in alleviating the psychological impact of trauma-related disorders. We also examine how age groups moderate the relationship between SOC and PTSD. Additionally, we explore the indirect effects of PSS and LS on the SOC-PTSD relationship. Conducting a cross-sectional study, we collected data from 321 individuals aged 65 and above (260 males, 61 females) across four Local Government Areas. Our data collection instruments included socio-demographic variables, the SOC instrument, the PTSD scale, the Multidimensional scale of PSS, and the LS index-z. Utilizing the Hayes Model 5 macro process for Structural Equation Modeling (SEM) analysis, our results highlight the significant moderating effect of age groups on the SOC-PTSD relationship. Furthermore, we find that PSS and LS partially mediate the relationships between SOC and PTSD, with SOC directly influencing both PTSD and PSS. These findings carry substantial practical implications and provide avenues for future research. In light of our study, we offer recommendations for addressing the mental health needs of Ekiti State’s elderly population, emphasizing the importance of further studies in the critical field of geronpsychology.

## Introduction

Post-traumatic stress disorder (PTSD) is a significant yet underexplored mental health issue among older adults in Nigeria. While PTSD is often linked to trauma or combat exposure at a younger age [1,2,3], it can also emerge later in life. In some cases, PTSD may develop as delayed-onset PTSD, a condition where symptoms of PTSD, such as intrusive memories, heightened arousal, and somatic complaints, manifest months or even years after the traumatic event [4,5]. However, PTSD in older adults is often complicated by aging-related factors, such as cognitive decline and limited coping resources, and the lack of adequate mental health support exacerbates these challenges. Many elderly Nigerians struggle with PTSD symptoms in isolation due to insufficient access to mental health care.

With approximately 75% of older adults in Nigeria experiencing multidimensional deprivation, including poverty, poor healthcare access, and social isolation [6], and a significant proportion facing severe hardship, the need for targeted interventions is critical. PTSD in later life poses distinct challenges, as aging-related cognitive and physical decline can exacerbate trauma symptoms and limit coping resources. In this context, perceived social support, life satisfaction, and the Selection, Optimization, and Compensation (SOC) model have emerged as crucial mechanisms for fostering psychological resilience. The SOC model provides a structured framework for adaptation by enabling older adults to select meaningful goals, optimize their available strengths, and compensate for functional limitations. When applied to PTSD management, SOC strategies may help older individuals mitigate trauma-related distress, strengthen adaptive coping mechanisms, and maintain overall well-being despite the constraints of aging [7].

This broader context of PTSD prevalence and the role of adaptive models like SOC becomes even more critical in State like **Ekiti State**, Southwestern Nigeria where systemic challenges such as widespread poverty, inadequate healthcare access, limited social support, and declining life satisfaction exacerbate the vulnerability of older adults to psychological distress [8, 9, 10]. Despite the potential of social support systems and adaptive models, significant gaps remain in understanding PTSD among older Nigerians, underscoring the urgent need for further research and targeted mental health interventions

Post-traumatic stress disorder (PTSD) can manifest later in life as delayed-onset PTSD, which is associated with psychiatric symptoms, somatic complaints, and hyperarousal [4,5,11–16]. In Nigeria, where one in four individuals are affected by mental health conditions [17,18], mental health services for older adults remain underdeveloped. Understanding the role of perceived social support (PSS), life satisfaction, and the Selection, Optimization, and Compensation (SOC) model in mitigating PTSD symptoms and enhancing well-being is critical.

PTSD can create a bidirectional cycle in which avoidance and isolation diminish PSS, leading to lower life satisfaction [19]. Conversely, higher PSS serves as a protective factor against PTSD [20]. Given this interplay, it is essential to explore how older adults can leverage adaptive mechanisms to mitigate PTSD symptoms. The SOC model, which focuses on optimizing resources and compensating for losses by prioritizing achievable goals, offers a valuable framework for understanding how older adults manage the effects of trauma [7].

Life satisfaction and PSS may mediate the relationship between PTSD and SOC, as stronger social support and higher life satisfaction help older adults adapt to trauma-related losses [2]. Empirical evidence supports the protective role of SOC strategies in mental health; for instance, Cruz & Ribeiro [21] found that SOC strategies significantly reduced depressive symptoms among older adults. Given the high comorbidity of PTSD and depression [22,23,24], these findings suggest that SOC may also buffer against PTSD by improving emotional well-being and coping capacity. Despite these promising results, the protective role of SOC in PTSD remains underexplored in research on older adults.

Selection, Optimization, and Compensation (SOC), introduced by Baltes and Baltes [7], describes strategies older adults use to manage aging-related challenges. SOC consists of selection (prioritizing goals amid cognitive and physical decline), optimization (investing resources to enhance functioning), and compensation (adopting alternative strategies when usual means fail) [25]. Given its role in promoting successful aging, SOC may help mitigate PTSD symptoms by fostering adaptive coping and maintaining well-being.

PTSD in older adults often coexists with depression, worsening mental health and functional impairment. This comorbidity is linked to recurrent depressive episodes, chronic illness, and lower life satisfaction [26,27]. Older adults frequently experience psychological distress through physical symptoms such as pain, sleep disturbances, and gastrointestinal issues—rather than explicitly identifying PTSD or depression [1,15]. These factors contribute to underdiagnosis and inadequate treatment. Exploring the role of SOC, perceived social support, and life satisfaction in PTSD management could inform targeted interventions for older adults in Ekiti State.

PTSD in older Nigerian adults remains largely understudied, with no data on its prevalence or trauma exposure. Research shows that older adults are difficult to study due to recruitment challenges, high dropout rates, and variations in symptom expression [28,29]. As a result, many studies exclude them or have insufficient sample sizes, limiting understanding of PTSD in this demographic [30,31].

Cultural factors also shape how PTSD symptoms manifest in older Nigerians. Often, distress is expressed through somatic complaints—such as burning or tingling sensations, headaches, or unexplained pains—rather than psychological terms, which can lead to misdiagnosis [32,33]. This somatization reflects a cultural tendency to externalize psychological distress, where mental health symptoms may not be openly acknowledged or discussed. Trauma-related cues, like loud noises, may trigger avoidance behaviors or physical reactions, further limiting daily functioning [1]. This cultural pattern complicates both the diagnosis and treatment of PTSD, as clinicians may focus on addressing physical symptoms without considering the underlying psychological trauma. Moreover, older Nigerians may seek help from traditional healers or community support systems rather than formal mental health services, which are often underutilized. These factors accentuates the need for culturally sensitive PTSD assessments tailored to Nigeria’s elderly population, ensuring that mental health professionals account for cultural norms and expressions of distress.

While there is limited research specifically focusing on PTSD among senior citizens in Nigeria, existing studies indicate that the disorder is prevalent across various groups, including displaced individuals, urban residents, and victims of road traffic accidents. Reported prevalence rates vary widely, ranging from 2.7% to 66.7%, depending on the population and methodology used [34,35,36,37]. This variation underscores the complexity of PTSD, which is influenced by a variety of social and environmental factors. Additionally, sociodemographic factors such as age, marital status, and socioeconomic status have been shown to correlate with the occurrence of PTSD, suggesting that these elements may also play a significant role in shaping the experiences of senior citizens with the disorder [34,37].

PTSD prevalence varies globally, with developed nations reporting rates in the single digits and developing nations often lacking comprehensive data. The World Health Organization (WHO), [38] estimates PTSD prevalence at 3.9% worldwide, though rates fluctuate based on diagnostic criteria, cultural perceptions, and trauma exposure. In Western nations, PTSD prevalence ranges from 2% to 7%, with lower rates among older adults [39,40]. In Asia, prevalence is typically lower, with China (0.3%) and Japan (1.3%) reporting reduced rates, potentially due to cultural stigma and differing symptom presentations [41,42]. These disparities underscore the need for region-specific research, particularly in underrepresented populations such as Nigeria’s elderly.

PTSD prevalence in Africa is significantly higher than in many other regions, particularly in conflict-affected areas. While data on older adults remain scarce, research indicates high PTSD rates in the general population. A meta-analysis found PTSD rates of 56.35% among internally displaced persons (IDPs) and 54.04% among refugees in Africa [43]. In Ethiopia, Uganda, and Kenya, prevalence rates range from 10.6% to 39.28% [44,45,46]. Post-conflict nations such as Sierra Leone and Liberia report even higher rates of 39.5% and 48.3%, respectively [47,48]. Among elderly individuals displaced by violence in Northern Nigeria, PTSD prevalence reaches 59.0%–61.5% [37], while Liberian refugees in Nigeria show rates as high as 76.4% [49].

The high burden of PTSD in Africa is driven by recurrent violence, weak mental health infrastructure, and socio-economic instability [50,51]. Additional contributing factors include road traffic fatalities [52], intimate partner violence [53], and political unrest. Economic hardship further restricts access to mental health care, particularly for older adults in under-resourced regions like Ekiti State, underscoring the urgent need for targeted interventions.

Ekiti State faces significant resource constraints shaped by a variety of contextual factors. With a poverty rate of 28%—substantially higher than Lagos State’s 4% [8]—the state struggles with economic insecurity, particularly among its older population. This challenge is compounded by a 54 billion Naira shortfall in unpaid retirement benefits, while the state only collects 43 billion Naira in PAYE taxes. Such financial strain hampers Ekiti’s ability to support retirees, leading to increased financial insecurity [54]. Additionally, the state has the highest fertility rate (4.7), dependency ratio (0.8), and life expectancy (61.9 years) in southwestern Nigeria [55]. These socio-demographic pressures intensify the demand on limited resources, making older adults particularly vulnerable to economic hardship and mental health challenges like PTSD. These economic constraints contribute to a lack of social support, a well-documented risk factor for PTSD.

Inadequate social support is a major risk factor for PTSD persistence [56,57,58]. Poverty exacerbates this issue by restricting access to resources and services [59]. In Nigeria, 63% of the population (133 million people) experience multidimensional poverty, affecting education, infrastructure, and financial stability [60,61]. With the elderly population projected to rise from 6% in 2030 to 9.8% by 2050 [62], the scarcity of PTSD research among older Nigerians is particularly concerning.

Despite Nigeria’s growing elderly population and their increasing mental health risks, epidemiological research on PTSD has often excluded older adults or failed to include them in sufficient numbers [31]. Scholars argue that senior citizens receive insufficient attention from policymakers, possibly due to perceptions of their lesser economic contributions [29]. To the best of our knowledge, there is a dearth of literature specifically addressing PTSD among older adults in Nigeria, leaving a critical gap in understanding the mental health challenges faced by this vulnerable population.

This lack of research is particularly concerning given Nigeria’s rapidly expanding elderly population, which is projected to reach 11.5 million by 2025, 16.5 million by 2030, and 25.5 million by 2050, making it the largest in Africa and the 11th largest globally [63]. Despite this demographic shift, research on PTSD among older adults in Ekiti State remains scarce, even though studies in Western countries highlight its prevalence in this age group [15,16,29,64]. While Nigeria approved the National Policy on Ageing in 2021, mental health policies addressing the needs of this growing population remain limited, underscoring the urgent need for research and targeted interventions.

As Nigeria’s elderly population grows, mental health challenges, particularly declining life satisfaction (LS), become more pressing. Reports indicate a decline in LS among senior citizens in Ekiti State [9,10], coinciding with a shrinking number of mental health facilities [65]. In contrast, older adults in Western countries report stable or improved mental well-being, experiencing fewer negative emotions, reduced stressors, and a more positive emotional balance [66, 67, 68]. This contrast underscores the need to understand factors influencing LS among Nigerian older adults. By examining the interplay between perceived social support, PTSD, and LS, this study aims to uncover key determinants of well-being and inform targeted interventions. However, systemic barriers, including a severe shortage of mental health facilities and professionals, further hinder older adults’ ability to maintain LS.

Nigeria’s shortage of mental health facilities and professionals severely limits access to care, especially for older adults [65,69]. With only 8 federal and 15 state mental health services, resources are inadequate, particularly in rural areas. The mental health workforce also faces critical shortages, hindering support for PTSD management and life satisfaction [70]. Given the growing elderly population and increasing strain on mental health services, understanding the link between PTSD and life satisfaction is essential.

Mental health data in Nigeria remains inconsistent, but estimates suggest 20–30% of the population—approximately 50 million people—experience mental illness, including PTSD [71,72]. Although the African Union recommended allocating 15% of the national budget to healthcare, Nigeria’s actual allocations declined from 5.95% in 2012 to 3.9% in 2018, with no dedicated budget for mental health services [73,74]. This lack of funding leaves older adults particularly vulnerable to psychological distress. The Selection, Optimization, and Compensation (SOC) model offers a framework for adapting to age-related challenges, but its potential to address PTSD and improve well-being in Nigeria remains largely unexplored.

In regions like Ekiti State, one of Nigeria’s poorest, socio-economic stressors—such as high poverty rates and resource depletion—add to the mental health burden [8,75]. Given the lack of mental health services and the vulnerability of the elderly population, exploring targeted interventions like SOC becomes essential in improving psychological outcomes for older adults in these areas.

While the SOC model has been linked to improved mental health outcomes in reducing depression and managing aging-related challenges [76], its application remains underexplored in Nigerian contexts. This study aims to investigate SOC as a potential intervention for PTSD among older adults in Ekiti State, shedding light on its capacity to enhance mental well-being in underserved populations.

Additionally, perceived social support (PSS) plays a crucial role in mitigating mental health challenges, influencing life satisfaction (LS) and overall well-being [77,78]. Although SOC has been studied in relation to life satisfaction, its interaction with PSS remains unexplored. Since both LS and PSS are known to mediate trauma’s effects, understanding their relationship with SOC could offer deeper insights into how older adults navigate PTSD and mental health challenges [3,78].

This study bridges this gap by exploring the intersections between SOC, PSS, and PTSD among older adults in Ekiti State. The findings aim to inform mental health interventions and policies tailored to the unique needs of Nigeria’s aging population.

The elderly population in Ekiti State consists mainly of the young-old (65–74 years, 54.7%), followed by the old-old (75–84 years, 35.9%) and the oldest-old (85 + years, 19.8%) [79]. While Selection, Optimization, and Compensation (SOC) strategies are widely studied elsewhere, they remain largely unknown and underutilized among older adults in Nigeria. Furthermore, research on SOC’s effectiveness is inconclusive—some argue it declines with age and resource deficits [80], while others suggest it persists across all age groups [81]. Given these gaps in the literature, this study seeks to examine the relationship between SOC strategies and PTSD using a moderated mediation model. In this model, perceived social support and life satisfaction are examined as mediators, while age groups serve as moderators.

PTSD in older adults is often under-recognized and under-treated, with crucial clinical details frequently overlooked or undisclosed by elderly patients [82,83]. Given the significant impact of PTSD on well-being, exploring effective management strategies is essential. While no direct studies have linked the Selection, Optimization, and Compensation (SOC) framework to PTSD, this model—focusing on optimizing resources and compensating for limitations [81]—shows promise in enhancing life satisfaction and mental health among older adults, particularly those with fewer resources.

With limited mental health funding and a high prevalence of PTSD among older adults [73,74], this study aims to examine how SOC strategies influence PTSD, perceived social support, and life satisfaction across different age groups (65–74, 75–84, and 85 + years). Specifically, it investigates the moderating role of age and the mediating effects of life satisfaction and perceived social support on PTSD outcomes. By identifying patterns across these groups, this research seeks to provide insights that can inform mental health interventions and policy decisions tailored to Nigeria’s aging population.

### Specific research questions

1. To what extent do SOC strategies influence perceived social support among senior citizens in Ekiti state?
2. How do SOC strategies relate to life satisfaction among senior citizens in Ekiti State?
3. What is the association between SOC strategies and PTSD among older adult senior citizens in Ekiti State?
4. How do perceived social support impact PTSD among senior citizens in Ekiti State?
5. To what extent does life satisfaction influence PTSD among senior citizens in Ekiti State?
6. How do the age groups (young-old, old-old, and oldest-old) moderate the indirect relationships between SOC strategies and PTSD?
7. How would the mediating roles of life satisfaction and perceived social support influence SOC and PTSD
8. How do age-related differences influence the overall mental health and well-being of individuals, considering variations in life satisfaction, perceived social support, PTSD, and SOC across different age groups?

### The significance of the study

This study is significant for several reasons, particularly in addressing the mental health challenges faced by senior citizens in Ekiti State, Nigeria. The unique demographic pressures in this region, including high fertility rates and dependency ratios, exacerbate the strain on perceived social support and life satisfaction, increasing the vulnerability of older adults to mental health issues such as PTSD. The research pioneers the exploration of Selection, Optimization, and Compensation (SOC) strategies in Nigeria, providing valuable insights into their potential to alleviate mental health challenges in resource-constrained settings. By investigating the previously unexplored relationships between SOC, PTSD, perceived social support and life satisfaction, the study aims to influence mental health support policies and interventions tailored to the needs of older adults. Moreover, by identifying significant age-related differences in these mental health variables, the study will inform targeted interventions, offering practical guidance to policymakers and healthcare professionals working to improve the well-being of seniors.

### Life satisfaction (LS) and Perceived Social Support (PSS) on PTSD

A key mechanism explaining the relationship between PTSD and mental health outcomes in older adults is the role of life satisfaction (LS) and perceived social support (PSS). Life satisfaction has been found to play a crucial role in psychological well-being, and social isolation has been linked to increased mortality risk (HR = 1.33, 95% CI: 1.26–1.41) [84]. Among elderly populations, social isolation may reduce life satisfaction, thereby increasing PTSD symptom severity. Strengthening social networks could therefore serve as an important intervention target to improve life satisfaction and mental health outcomes in senior citizens

Research suggests that senior citizens’ levels of PTSD are influenced by both their life satisfaction and perceived social support [3]. PSS, a cognitive construct embedded in personality traits, refers to the belief that family, friends, and significant others are available to provide help, advice, or comfort when needed [85]. According to the stress-buffering model, social support mitigates the psychological effects of stress by reducing the perception of threat in response to a stressful event [86]. After such an event, PSS can facilitate cognitive and emotional processing, allowing individuals to reframe the situation and adapt more effectively [87,88]. When individuals believe that they have access to the necessary support resources, they are more likely to perceive stressful events as manageable, which in turn can reduce the likelihood or severity of PTSD symptoms. Understanding the impact of PSS and LS on PTSD in older adults can provide valuable insights for developing targeted mental health interventions.

While research highlights the role of life satisfaction (LS) and perceived social support (PSS) in reducing PTSD symptoms among senior citizens, the concept of PSS remains underappreciated in the Nigerian context. Despite its significance, the concept of perceived social support (PSS) remains underappreciated in Nigeria, where scholars often conflate it with received social support, neglecting PSS’s critical role in mental health outcomes [89, 90, 91]. Perceived social support refers to the belief that one is part of a social network of mutual obligation [92], while received social support is the tangible emotional or instrumental support given by others [93]. Although received support has shown mixed results in research, PSS consistently correlates with lower stress and better mental and physical health [94]. This gap in understanding and appreciating PSS in Nigeria underscores the importance of this study in exploring its impact on PTSD among older adults.

Given this lack of clarity and understanding regarding the distinction between perceived and received support, it is essential to note that previous studies have shown that, in general, PSS is correlated with improved physical and mental health [95,96]; and that PSS is more critical than received support in predicting adjustment to life stress [97]. On the other hand, researchers have found that in stressful situations, received social support is positively linked to negative affect [98] and depression [99]. However, the relationship between received social support and health outcomes is often negligible and inconsistent [96,100]. In contrast, the association between perceived social support (PSS) and health is consistently positive [96,101,102].

Building on the understanding that perceived social support is critical for mental health outcomes, it is particularly vital to recognize its role as a determinant of life satisfaction among older individuals. Indeed, PSS is an essential determinant for improving the LS of older people [103,104]. In recent years, PSS has been confirmed to be a significant factor influencing LS across various population groups, including older people [105]. Studies have shown that perceived social support significantly predicts both life satisfaction and negative psychological outcomes, such as anxiety, depression, and distress [99,106]. Conversely, the absence of PSS from family, friends, and significant others has been widely proven to decrease one’s life satisfaction [107]. Although the impact of PSS on life satisfaction is well established, the underlying mechanisms that explain this relationship remain complex and not fully understood [108]. In this context, little attention has been given to the specific relationship between PSS and LS among elderly people, especially in Ekiti State.

As established, perceived social support plays a vital role in influencing mental health outcomes and life satisfaction among older adults. Neugarten, Havighurst, and Tobin [109] define life satisfaction as the extent to which an individual has a positive self-concept and feels their past goals align with their current living conditions. For older adults, life satisfaction is closely related to various behavioral, psychological, and social outcomes, such as reduced stress and decreased externalizing behaviors [110]. Research indicates that individuals with higher levels of perceived social support are less likely to experience negative mental health outcomes, such as anxiety, psychological distress, and depression, leading to greater overall life satisfaction

In addition, post-traumatic stress disorder (PTSD) can significantly impact an individual’s well-being, particularly among older adults who have experienced life-threatening events. According to the Diagnostic and Statistical Manual of Mental Disorders (5th ed., text rev.; American Psychiatric Association, [111], PTSD can lead to severe distress and impair daily functioning in family, social and occupation. Numerous studies have indicated that common PTSD symptoms among senior citizens include hypervigilance, sleep disturbances, anger, difficulty concentrating, fatigue, muscle tension, and pain [15,16,64]. Furthermore, some researchers suggest that PTSD symptoms in older adults are often accompanied by somatic complaints and hyperarousal symptoms [15,16].

Recent findings indicate that as individuals with PTSD age, their avoidance and arousal symptoms may persist or worsen, while re-experiencing symptoms tend to decrease [28]. This shift in symptom profile can profoundly impact social interactions and overall life satisfaction. Moreover, research shows that being socially isolated increases the risk of early death by about 33%—with the true risk rising between 26% and 41% [84], underscoring the critical role of perceived social support in overall health. Given that strong social support is a well-established protective factor against PTSD, enhancing social support networks may be vital for mitigating PTSD symptoms among senior citizens in Ekiti State. For these older adults, the combination of limited social support and persistent PTSD symptoms can further challenge their quality of life. Therefore, understanding and addressing the specific needs of this population is crucial for developing targeted interventions aimed at improving their mental health and well-being.

However, studies examining the relationships between perceived social support (PSS) scores and PTSD incidence are notably inconclusive, with limited research correlating these two variables. Nevertheless, PSS plays a crucial role in moderating stress and depression and is associated with positive health behaviors and a sense of security among senior citizens [112]. Indeed, poor perceived social support has been identified as one of the most significant risk factors for the maintenance of PTSD and the development of its symptoms [56–58,113,114], as well as a lack of life satisfaction ( [59].

In light of these factors, it is essential to consider the broader socio-economic context. A recent report from the World Bank [61] forecasts that approximately 13 million Nigerians fall below the poverty level, while the National Bureau of Statistics [60] indicates that 63% (133 million people) of Nigerians are multidimensionally poor, encompassing monetary poverty, education, and access to basic infrastructure services. Notably, Ekiti State in southwestern Nigeria stands out as the poorest state, with a poverty headcount rate of 28%, in stark contrast to Lagos, which is the wealthiest state in the country [8]. Furthermore, Ekiti State exhibits the highest dependency ratio and total fertility rate in Southwest Nigeria, at 0.8% and 4.37%, respectively [75].

These demographic factors make Ekiti State a resource-depleting area, potentially leading to poor perceived social support and diminished life satisfaction, which may exacerbate PTSD among elderly individuals. However, this situation has yet to be thoroughly explored by scholars. Conversely, several researchers have characterized perceived social support (PSS) as a relatively stable trait [115,116], sharing commonalities with personality dispositions [117]. Nevertheless, PSS can also fluctuate over time, depending on an individual’s living circumstances, current mental health status, and exposure to positive or negative life events [118,119].

Moreover, exposure to severe stressful events can significantly impact perceived social support [120]. Individuals coping with such stressful experiences may struggle to engage in trusting relationships [118,119]. Interestingly, research presents mixed results regarding whether PSS mitigates, elevates, or remains constant during stressful situations [118,119,121,122]. Additionally, evidence suggests that greater psychological symptoms lead to lower levels of PSS [119,123,124], often due to the avoidance of PTSD symptoms among seniors [125]. Psychological symptoms, including PTSD and depression, can alter individuals’ thought processes and negatively affect their sense of social support [126,127]. Another potential common cause of lower PSS scores could be a history of trauma, which can be emotionally distressing. Exposure to traumatic events often contributes to the development of psychopathology [128] and is also associated with lower perceived social support [118].

Indeed, Kaniasty and Norris [119] examined individuals exposed to natural disasters and showed that more PTSD symptoms were associated with less perceived social support (PSS) 18 and 24 months post-trauma, supporting the notion of social selection, which posits that PTSD can influence social support. This highlights the critical role of PSS in the context of trauma. In a previous study, PSS was shown to mediate the relationship between challenging life events and psychological distress, such as anxiety, depression, and behavioral issues [129]. Additionally, research has demonstrated that higher levels of perceived social support are associated with more positive PTSD recovery outcomes [130].

Building on the understanding of perceived social support (PSS) and its relationship with post-traumatic stress disorder (PTSD), it is essential to consider social causation or social deterioration theory, which explains how PSS can negatively impact PTSD. This theory posits that PTSD erodes social support because individuals with PTSD often develop an increased belief that others are dangerous and unsafe [131]. Such perceptions suggest that poor mental health diminishes social support [132,133]. Consequently, individuals with PTSD may find it challenging to establish trust, leading to increased social isolation [132]. From this perspective, researchers have proposed that PTSD symptoms such as insecurity, skepticism, social avoidance, and social isolation—can contribute to social rejection and reduced PSS and life satisfaction (LS) from others [19]. Additionally, a study of adults exposed to physical assault in Norway found that higher levels of PSS protected against the development of PTSD symptoms, while diminished PSS increased the risk of developing such symptoms [20]. Many scholars have built upon this perspective, suggesting that due to the avoidance and re-experiencing of PTSD symptoms, PSS could have a negligible effect on the disorder [132,133].

In light of the existing literature, it is important to note that while previous research has explored various aspects of life satisfaction (LS) among senior citizens in Nigeria, particularly in Ekiti State [134–136], none have examined the influence of LS on the incidence of posttraumatic stress disorder (PTSD). For instance, Odetola et al. [134] conducted a study on LS among senior citizens living in geriatric homes in Lagos, finding that most participants reported general satisfaction with their lives. However, loneliness, childlessness, and illness were identified as significant contributors to life dissatisfaction. In Ekiti State, Arogundade and Adebayo [9] highlighted that LS decreases with age and that family relationships are a key predictor of life satisfaction. More recently, a cross-sectional study of hospitalized patients aimed to determine the association between social support and LS among older adults in Ekiti State. The results revealed that 73.8% of senior citizens reported dissatisfaction with their lives, primarily due to a lack of government support, with 81% relying on their relationships for assistance [10]. Collectively, these findings suggest low levels of life satisfaction in Ekiti State, with contributing factors including loneliness, childlessness, and inadequate government assistance. This raises concerns about potential mental health challenges, such as PTSD, among senior citizens in Nigeria; however, no studies have yet investigated this area.

While studies in Nigeria have not explicitly linked life satisfaction (LS) with post-traumatic stress disorder (PTSD), some limited research elsewhere has identified associations between the two. For instance, a study assessing community samples found that individuals with severe PTSD reported lower levels of life satisfaction compared to those with other mental health conditions or no diagnoses at all [137]. Furthermore, a cross-sectional study among police officers following Hurricane Katrina in New Orleans examined the relationships among resilience, gratitude, life satisfaction, and PTSD. The results indicated that positive factors such as resilience and life satisfaction could help mitigate PTSD symptoms [11]. This finding aligns with research suggesting that older adults generally report higher life satisfaction and positive affect, alongside lower negative affect, compared to younger adults [138–140]. Additionally, another study indicated that individuals with moderate or severe PTSD are more likely to experience low levels of life satisfaction [137]. Among military veterans in the United States, meaning in life was found to correlate with lower levels of PTSD symptoms [2]. Another study suggested that individuals with low life satisfaction may experience worse PTSD symptoms than those who report high life satisfaction [11]. While these studies are correlational and do not establish a causal relationship between PTSD and LS, they highlight the important roles that perceived social support (PSS) and LS play in the physical and mental health of senior citizens [77,78]. To resolve this ambiguity, we propose that perceived social support (PSS) and life satisfaction (LS) serve as explanatory variables for PTSD in this study.

Understanding how PSS and LS interact with the mental health challenges faced by this population can provide valuable insights into developing effective interventions aimed at enhancing their quality of life and mental resilience. In transition, understanding the interplay among SOC, PSS on PTSD can offer valuable insights into the mental health and well-being of older adults in Nigeria, ultimately informing the development of effective interventions to enhance their quality of life.

### SOC, PSS, ON PTSD

Selection, optimization, and compensation (SOC) is a model developed by Baltes and Baltes [7] as a process that older adults can use to cope with life stressors associated with aging [25]. The meta-theory posits that three life management processes—selection (a categorical choice for gain), optimization (a method for maximizing gain), and compensation (a strategy for minimizing losses)—lead to successful aging [7]. This theory serves as a life management model for adjusting to physical and behavioral changes [25]. According to the theory, individuals expend energy to maintain physical and emotional stability while seeking ways to compensate for losses and become more proficient in activities they already excel in [141]. Adult life experiences are characterized by a shift toward fewer resources gained and more resources lost, signifying that aging involves both growth and decline [142]. Additionally, Baltes et al. [141] noted that aging is primarily associated with gains for young people but predominantly with losses for older adults, as adult development increasingly shifts toward resource depletion [142]. Similarly, Blanchard-Fields et al. [143]suggested that SOC could serve as a model of coping [144], problem-solving [143], self-development, and goal adjustment [145].

Expanding on the SOC model’s theoretical foundations and its potential role in managing the aging process, we also consider how SOC strategies might intersect with mental health outcomes, particularly PTSD. However, we perceive SOC awareness and usage as emotional constraints that could exacerbate PTSD symptoms in elderly individuals. We therefore assume that SOC is significantly related to PTSD, potentially mediated by perceived social support (PSS). This assumption is supported by a study suggesting that greater adherence to SOC strategies was correlated with higher levels of physical health and stress among older adults with multimorbidity, where greater disability led to greater use of SOC strategies [146].

To our knowledge, there is limited literature exploring the relationship between SOC and PTSD, with only a few studies examining the connection between PSS and SOC. Furthermore, proactive strategies promoting SOC have been integrated into optimal psychotherapy approaches, particularly in cases of poststroke depression [147–149]. The SOC model has also been extended to encompass social aging, acknowledging the pressures imposed by social relationships [150], which may have implications for the relationship between PSS and the development of PTSD, particularly in older adults [151]. Additionally, differing perspectives on this concept exist [152]. For instance, Shirzadifard, Shahghasemi, & Hejazi [152] found that the implementation of SOC strategies led to significant variability in life satisfaction and was associated with increased depressive symptoms [81].

In addition to the theoretical considerations, some studies suggest that SOC is mood-dependent and tends to diminish with age, as resource deficits in advanced age may limit the effectiveness of SOC strategies [80,81,150]. Moreover, SOC has been viewed as a trait variable, potentially influencing the risk of depression and, in some cases, contributing to psychological deterioration [76]. However, this claim has been contested by scholars who argue that SOC should be considered a therapeutic variable, adaptable and beneficial in clinical interventions [153,154].

Alternatively, the use of SOC strategies has been found to significantly exacerbate depressive symptoms, as well as behaviors such as drinking and smoking [155]. However, more recent studies have contradicted these findings [150]. As such, it appears that SOC may function as both a state and trait variable, with the potential to contribute to dysfunctional behavior over short and long time frames, which could increase the risk of developing PTSD among senior citizens.

Indeed, previous studies have overlooked the potential association between SOC and PTSD through the mechanism of PSS. To address this gap, we propose that the relationship between SOC and PTSD could be mediated by PSS and LS. While existing research has not demonstrated a direct link between SOC and PTSD through LS or PSS, we suggest that SOC’s relationship with PTSD may be aligned with its connection to depression. Studies have shown significant overlap in the symptoms of depression and PTSD, including insomnia, decreased concentration, avoidance, withdrawal, lack of interest, social distancing, and anhedonia [22,156]. Additionally, the comorbidity rate of PTSD and depression ranges from 54.41% to 54.72% [22]. Further, depression has been shown to elevate the risk of developing PTSD after trauma exposure [157], while PTSD increases the likelihood of first-onset depression following trauma [23,24]. These correlations suggest that SOC may influence PTSD among elderly people, yet to date, no study has thoroughly examined this relationship.

Given the key aspects of SOC, such as resource loss, social convoy selection, adjusting to new environments, seeking help from significant others, and modifying relationships, it is plausible to suggest that PSS could help explain the relationship between SOC and PTSD for the first time in the literature. PSS is a vital social resource that plays an essential role in managing or coping with stress and has been strongly associated with psychological well-being during stressful times [158]. Compared to received social support, self-compassion has also been closely linked to an individual’s ability to adapt, regulate emotions, adjust, and cope with stress [158]. Specifically, one study showed that after leaving a war zone, veterans who perceived higher levels of support upon returning from the battlefield experienced less intense PTSD symptoms and reported better mental health and quality of life compared to those who felt less supported [159].

A meta-analysis indicated that lacking a network of meaningful relationships in life is a stronger predictor of mortality than certain unhealthy behaviors, such as smoking or physical inactivity [160]. Furthermore, cross-sectional research has consistently shown a negative relationship between social support and PTSD. Individuals who survive disasters but have higher levels of social support exhibit less severe PTSD symptoms, while those with more severe PTSD symptoms report lower levels of social support [56,58,132]. Conversely, perceived social support plays a more critical role in emotional and psychological well-being than the mere presence of a large social network or the type of support offered [119]. Research also suggests that perceived social support mediates the relationship between overwhelming disasters and psychological outcomes such as stress, emotional distress, and behavioral issues [129]. However, it is widely recognized that poor perceived social support is one of the most significant risk factors for both the development and persistence of PTSD symptoms [56,57,113].

Although there is a research gap regarding the relationship between SOC and PSS, Hobfoll’s Conservation of Resources (COR) theory [161] provides a valuable framework for bridging this gap by explaining the mechanisms and interactions between these two concepts. COR theory is particularly useful for understanding how individuals manage and accumulate primary, secondary, and tertiary resources to cope with stressors and challenges. In the context of this study, COR theory is especially relevant because it extends beyond simply correlating resources with performance and mental health issues among elderly individuals. Emerging from resource and psychosocial stress theories, COR theory [161,162] highlights how people mobilize and invest resources to mitigate stress and enhance well-being. Applied to the relationship between perceived social support and SOC strategies, COR theory provides insight into how individuals strategically allocate their resources to maintain and improve their well-being. Perceived social support, as a subjective psychological resource, can be conceptualized as a critical asset that elderly people can draw upon when facing stressors or adversity. Through the perception of support from their social network, they can gain emotional comfort, validation, and practical assistance in coping with challenges.

Meanwhile, SOC strategies involve actively managing resources to adapt to changing circumstances and demands [25]. These strategies include selecting the most relevant goals and relationships, optimizing the use of available resources, and compensating for potential deficits. This approach aligns with Hobfoll’s COR theory, which posits that individuals strive to acquire, retain, and protect resources, as losses or threats to resources can lead to adverse outcomes such as stress and psychological distress [162,163]. In the context of the interplay between perceived social support and SOC strategies, COR theory suggests that individuals who perceive greater social support may have access to a larger resource pool [163]. This expanded resource pool could enhance the effectiveness of SOC strategies, as individuals with a strong support network are likely to experience lower stress levels, providing them with more energy and capacity to engage in adaptive coping behaviors.

Building on this understanding of SOC strategies as adaptive mechanisms for managing resources and stress, the role of perceived social support becomes particularly relevant. The perceived availability of social support may act as a buffer against resource loss, thereby reducing the impact of stressors on an individual’s well-being. When faced with challenges, individuals can rely on perceived social support to alleviate stress and reduce the need for extensive resource compensation [86]. This concept aligns with the core principle of COR theory, which emphasizes the preservation of resources. However, SOC, as a psychological and behavioral management model for adapting to change [164], offers additional insights into this dynamic. Therefore, we speculated that SOC will significantly influence PSS, potentially bridging this knowledge gap. SOC strategies encompass various techniques for managing the multitude of changes that occur throughout the lifespan [7]. The model also explains how loss-based selection redirecting energy in response to the loss of previously available resources can lead to psychological and physical distress [25].

This challenge is compounded by the fact that optimization and compensation, which are central to SOC strategies, become increasingly effortful and demanding with age, often exceeding the individual’s social and technical resources [25]. In this context, perceived social support (PSS) could mediate the relationship between SOC and well-being, as SOC compensation often involves seeking help from others, developing new skills, and devoting more energy and time to manage changing circumstances [165]. Research has shown that PSS is significantly associated with personality traits [166,167], and cognitive and personality factors are considered the most stable predictors of SOC management strategies [25]. Furthermore, Baltes [168] demonstrated that SOC strategies predict unique outcomes in intellectual functioning, thinking styles [169], and adaptive coping behaviors, such as flexible goal adjustment and tenacious goal pursuit [170]. On a behavioral level, Lang, Rieckmann, and Baltes [171] found that individuals with rich social–personality resources (e.g., those who enjoy social relationships, seek help, and maintain physical closeness) exhibited more frequent use of SOC strategies compared to resource-poor older adults. The SOC model complements various coping models [144], problem-solving approaches [143], life satisfaction development, and goal adjustment frameworks [164,172]. A recent line of inquiry has suggested that SOC might influence PTSD through PSS, though no study has yet specifically examined this relationship. Given the prevalence of PTSD among elderly individuals, addressing this gap in the literature is essential. SOC could be viewed as a trait variable that either increases or decreases the risk of developing PTSD [76]. Therefore, understanding how SOC and life satisfaction interact with PTSD is crucial for developing effective interventions to enhance the psychological well-being of older adults. Exploring the interplay between SOC and PSS is equally important for understanding their potential role in mitigating PTSD symptoms and ultimately enhancing the psychological resilience of older adults.

### Selection Optimization Compensation (SOC),  LS, and PTSD

Two theories explain the use of SOC (selection, optimization, and compensation) strategies. The first theory posits that as individuals age, they become more adept at utilizing SOC strategies due to the accumulation of life experiences. In contrast, the second theory suggests that biological and physical limitations associated with aging lead to a loss of resources, which may restrict the use of SOC strategies [7]. This second perspective illustrates how resource depletion can exacerbate depression in older adults and increase the likelihood of developing PTSD. As a result, aging becomes associated with SOC-related declines, where behavioral adaptation may diminish [25].

This connection between resource depletion and SOC-related declines further highlights how the limitations in adaptive behavior may contribute to psychological health challenges, such as PTSD, which could be influenced by life satisfaction (LS). The SOC model emphasizes that an individual’s resources such as mental, physical, and social are finite, and as people age, these resources are spread thinner across multiple domains. This decline in physical activity and adaptive behavior may contribute to psychological health challenges, such as PTSD, which could be influenced by life satisfaction (LS). The notion that life-management strategies (SOC) become more intricate with diminishing resources is supported by research [173]. However, studies examining the relationship between SOC, life satisfaction, and PTSD remain scarce in the literature.

Life satisfaction refers to a person’s overall evaluation of their subjective quality of life or general well-being [174]. It is often considered a key predictor of successful biopsychosocial adaptation in later life when individuals face losses in health, physical and cognitive functioning, social networks, and productive activities [175–177]. Interestingly, despite these age-related losses, many older adults do not experience a corresponding decline in life satisfaction, a phenomenon known as the “paradox of well-being.” Despite natural decreases in health, income, and physical or cognitive abilities, older adults often maintain high levels of life satisfaction by making accommodative shifts aligning their goals and aspirations with what is realistically achievable [178,179].

Furthermore, older individuals’ ability to sustain life satisfaction in later life may be attributed to an enhanced capacity for emotional regulation and proactive engagement in positive emotional experiences [139,180]. This framework sets the stage for exploring how SOC strategies and life satisfaction interact and potentially influence PTSD outcomes in older populations.

Similarly, socioemotional selectivity theory posits that older adults use their accumulated life experiences to effectively manage and avoid negative encounters by selectively choosing social partners—such as family and friends—who offer positive emotional feedback and support [181,182]. This theory aligns with research suggesting that older adults generally report higher levels of life satisfaction compared to younger adults [183]. Additionally, older adults tend to experience and engage more with positive emotions, paying closer attention to and recalling positive stimuli more readily than younger adults [184]. Supporting this notion, Inglehart’s [185] analysis of Eurobarometer surveys (1980–1986) and the World Values Survey found that, when controlling for factors like income, education, and marital status, people aged 65 and older demonstrated higher levels of life satisfaction and happiness compared to younger age groups.

However, most of these studies have been conducted in developed countries with high GDPs, advanced healthcare systems, higher income levels, and longer life expectancies, while fewer studies have focused on developing nations or Africa. For instance, Lucas and Gohm [186] found that the relationship between age and life satisfaction varied across countries—being positively related in some and negatively related in others. Similarly, happiness, as a measure of subjective well-being, tends to be greater in more economically developed countries, as indicated by higher GDP per capita [187]. Deaton’s [188] research using the Gallup World Poll revealed a linear decline in life satisfaction with age in most countries, with the most significant declines observed in lower-GDP nations, while mid-GDP countries showed a smaller decline. Moreover, socio-demographic factors, including marital status, income, education, number of children or wives, age, and religion, have been identified as determinants of life satisfaction in Africa, particularly in Nigeria [136]. Numerous studies suggest that being married, employed, religious, having better health, higher income, receiving social support, and achieving higher levels of education are all positively linked to various aspects of well-being [189–192]. Additionally, in Nigeria, having children and higher educational attainment are positively associated with life satisfaction [136].

Building on the discussion of socio-demographic factors and their impact on life satisfaction, the SOC framework offers a valuable lens for understanding how older adults adapt to aging-related challenges. From this perspective, life satisfaction (LS) is seen as a key indicator of psychological adaptation, with SOC strategies acting as a means of managing resources [193]. According to this framework, older adults amplify the positive effects of gains and minimize the negative effects of losses by selectively investing in achievable, optimal goals. In doing so, they compensate for their losses and limitations [193]. By focusing on growth-oriented goals, they maintain or even enhance their life satisfaction, despite age-related declines [193]. Research has consistently shown that greater use of SOC strategies at advanced ages leads to higher levels of well-being [173,194,195]. For instance, Jopp and Smith [173] demonstrated that SOC strategies—whether applied individually or in combination—mitigate the adverse effects of lower demographic (education), cognitive (perceptual speed), health (balance), and social resources (number of social partners) on well-being and life satisfaction. Moreover, recent studies indicate that SOC-based interventions may have therapeutic benefits, reducing depression and enhancing the well-being of older adults in clinical settings [154]. This growing body of research suggests that life satisfaction can act as a protective mechanism, preserving health, reducing vulnerability to serious illnesses, and promoting longevity through positive emotions that foster a more active lifestyle and greater self-care motivation [196].

Expanding on the relationship between SOC strategies and well-being, it is evident that life satisfaction can be achieved through the intentional setting of goals and effortful behaviors, as proposed by the SOC model. Several studies have confirmed the connection between SOC and mental health, though the findings have sometimes been ambiguous [173,197–199]. For instance, a study examining the daily use of SOC strategies across different age groups found that older individuals who applied more selection, optimization, and compensation strategies than their weekly average reported increased happiness. Specifically, both older and middle-aged adults demonstrated a positive association between daily SOC usage and happiness [173]. Additionally, Freund [197] reviewed evidence suggesting that SOC strategies enable individuals to use their resources more effectively, thereby enhancing life satisfaction. Further, Jopp and Smith’s [173] research, which compared young and older adults, revealed that SOC was significantly related to available resources and aging satisfaction. Notably, the association between resources and life satisfaction persisted when comparing younger-old and older-old individuals separately, reinforcing the role of SOC in maintaining well-being across different stages of aging.

Elaborating on these findings, it was observed that the relationships between SOC strategies and available resources were weaker in old-old individuals compared to young-old individuals. Additionally, SOC strategies were significantly correlated with aging satisfaction only among the young-old, but not in the old-old population [173]. This indicates that the concurrent effects of resources and SOC usage, which have shown strong correlations with well-being indicators in younger adults [198], may benefit individuals across adulthood and thus warrant further examination [199]. Furthermore, a study examining the impact of SOC-based psychotherapy on depression and well-being in older adults revealed that SOC interventions had significant therapeutic effects, reducing depression severity and increasing the overall well-being of senior citizens [199].

In contrast, PTSD has been associated with delayed onset in older adults [4,5,11] and other psychiatric symptoms [12, 13, 14], which may lead to lower life satisfaction. While there is evidence of a relationship between life satisfaction (LS) and PTSD [2,137], individuals who are dissatisfied with life are more likely to suffer from psychiatric conditions, including psychological distress, have an increased risk of suicide, and experience higher mortality rates [12, 13, 14]—all of which can be comorbid with PTSD [200]. Furthermore, one study found that people with moderate to severe PTSD are more likely to report low levels of life satisfaction [137], while meaning in life was shown to correlate with reduced PTSD symptoms [2]. These findings suggest, for the first time, that life satisfaction could be a plausible explanatory variable for the link between SOC and PTSD.

Despite the absence of studies explicitly linking SOC to PTSD through life satisfaction, we aim to address this knowledge gap. Existing research indicates that a significant decrease in SOC competencies is associated with depressive symptoms [150]. Conversely, some studies have shown that an over-reliance on SOC strategies can lead to psychological distress. It appears that individuals facing physical and emotional challenges may be resistant to using SOC strategies, which can exacerbate their distress [146,171]. Yuen and Vogtle [146] explained that a rigid adherence to SOC methods is correlated with heightened physical health stressors. Similarly, in a study involving older adults with multimorbidity, greater disability resulted in increased utilization of SOC strategies [146]. However, the authors found that SOC strategies did not correlate with loneliness or depressive symptoms in women. In a longitudinal study, Lang, Rieckmann, and Baltes [171] reported that resource-rich participants were more likely to employ adaptive SOC strategies compared to resource-poor participants. Considering the mixed findings, it is crucial to investigate how age moderates the relationship between SOC strategies and PTSD. Age-related factors influence resource availability and adaptive behavior, making it important to explore whether older adults are more vulnerable to PTSD or if age helps buffer the impact of SOC strategies on psychological distress.

### Age groups as moderators of SOC and PTSD

Gorman [201]) posits that aging is a biological reality and process that no human can control. According to Shock [202], when aging occurs in humans, physiological development is usually accompanied by psychological and behavioral changes and other changes involving social and economic factors. In their quest to find solutions to some of these challenges imposed by aging, scholars from the beginning of the mid-1980s developed considerable models that proposed that aging individuals can boost their health [197,203,204] while avoiding decreases and losses by taking responsibility for their health [205] and adapting their health behaviors [206].

Studies focusing on the use of the SOC procedure in very old adults could help researchers investigate the moderating effects of age groups on the association between SOC and PTSD. Age stratification and age groups (young-old, old-old, oldest-old) led to considerable content among scholars, policymakers, and social gerontologists. To this extent, there has yet to be a consensus among educators as to what constitutes age groups. Furthermore, many scholars have said that such conceptualization connotes ageism and should be disregarded [207]. Nevertheless, despite these contentions and misunderstandings, age stratifications have been adopted in policy documents, and today, the plurality of aging studies and policy documents use age bands to define an old population [208]. The most common approaches in the aging literature include 65–74 (year (young old), 75–84 years (old-old), and 85 plus (oldest old) Neugarten [209].

Considering these factors, our reason for adopting this categorization in the present study is that the Age Harmonization Act of 2012 and 2022 of Nigeria put the retirement ages of judicial officers and professors of tertiary institutions at 70 years each and 65 years, respectively, for secondary and primary schoolteachers and judicial staff. People aged 65–74 years, both young and old, are among the growing senior citizens in the Ekiti state and Nigeria. According to the National Population Commission [79], young old constitute two-thirds (63%) of senior citizens nationwide, and in Ekiti State, this demographic sector comprises approximately 54.7%. Again, 35.9% of the elderly populations are aged 75–84 years, and this demographic population is also on the rise [79]. Similarly, the oldest individuals (85+) in the state are few but increase in number. Recent data show that the oldest old people constitute 19.8% of the total population in Nigeria [210].

The inclusion age groups as a moderator is theoretically grounded in the Selection, Optimization, and Compensation (SOC) model [80], which explains that aging involves a progressive shift from resource acquisition to resource depletion. This shift may influence an individual’s ability to effectively utilize SOC strategies, potentially affecting psychological resilience, particularly in response to PTSD symptoms. While SOC theory has been widely applied in aging, well-being, and cognitive adaptation research, no study to date has examined its role in PTSD outcomes. Given that PTSD symptoms often involve disruptions in coping mechanisms, it is critical to explore whether SOC—an adaptive strategy for managing resource constraints—can buffer against PTSD symptoms, and whether this effect varies across different age groups.

Empirical findings on SOC usage across the lifespan remain inconsistent. Freund and Baltes [81] found a negative age association with SOC strategy use in older adults (73–103 years), suggesting that as age increases, the ability to optimize and compensate declines due to diminishing cognitive and physical resources. In contrast, Baltes [211] reported that SOC behaviors are observed across all age groups, with middle-aged adults demonstrating the highest engagement, likely due to an optimal balance of available resources.

Given these contradictory findings and the lack of research on SOC in PTSD, a moderation analysis is necessary to examine whether age differentially influences the effectiveness of SOC strategies in reducing PTSD symptoms. A significant moderation effect would suggest that the protective role of SOC in PTSD varies across age groups, offering insights into age-specific interventions. Thus, this study fills an important gap in the literature by being the first to apply SOC theory to PTSD symptoms, while also addressing whether age influences the SOC-PTSD relationship. By clarifying this relationship, the findings could inform more tailored intervention approaches for individuals at different life stages.

In general, SOC strategies appear to be particularly beneficial for older individuals with fewer available resources [171,173] and for younger adults [212]. Freund and Baltes [81] found that SOC strategies are linked to positive outcomes even in advanced old age, as demonstrated in a study involving participants aged 72–102 years. Research comparing young-old individuals (approximately 70–80 years) and oldest-old individuals (80–90 years) showed that satisfaction with aging was higher when sufficient resources were available. Moreover, the use of SOC strategies independently increased satisfaction, but only in the young-old group [173]. For old-old individuals, greater resource availability significantly contributed to aging satisfaction, whereas the use of SOC strategies only made a difference when resources were insufficient. Thus, for the oldest individuals, having sufficient resources is the key factor in life satisfaction [173].

On the other hand, resource deficits especially in areas such as physical and functional health, social networks, and cognitive capacity are well-documented across many cultures for young-old, old-old, and oldest-old individuals, becoming more pronounced as people reach the oldest age groups [103,213]. It has been suggested that the decline in the use of the three SOC components (selection, optimization, and compensation) from middle to late adulthood is linked to the depletion of these resources. However, older adults can still effectively compensate for these deficits by utilizing SOC strategies more efficiently [214,215].

However, some suggest that the use of SOC strategies across different age groups may be linked to adverse mental health outcomes, particularly depression [173]. For instance, a study on hospitalized patients found no significant differences in SOC competencies between younger and older adults in terms of depression severity, both at admission and during remission [150]. SOC is considered a trait variable that may either increase or reduce the risk of depression and relapse [76], while depression itself can impact SOC competencies [150]. Interestingly, when participants were treated using SOC strategies, the middle-aged group (50–66 years) showed the best outcomes compared to younger and older groups [150]. A separate study on workers also found no evidence of a decline in SOC competencies with age, suggesting that these strategies may be maintained throughout the lifespan [216]. Although SOC strategies help counter resource deficits and promote well-being into old age, their impact on other aspects of mental health, particularly PTSD, remains largely unexplored. It is essential to investigate how age may moderate the effectiveness of SOC strategies in specific contexts, such as PTSD, as age-related changes in coping resources and health can potentially influence how older adults experience and manage PTSD symptoms.

According to Kaiser et al. [217], it is common in the aging literature to conceptualize older adults into subgroups: young-old (ages 65–74), middle-old (75–84), and old-old (85 and older) [209]. However, the PTSD literature on older adults is relatively limited and has not fully adopted this type of categorization [217]. This gap highlights the importance of examining whether age-related differences influence mental health outcomes in older adults, particularly in how they experience and manage PTSD. Given the potential variability in both coping resources and PTSD symptoms across age groups, studying the moderating role of age in the effectiveness of SOC strategies is crucial to understanding how older adults across different PTSD is not a static condition; its symptoms fluctuate throughout the lifespan, influenced by life stressors, significant events, and physiological changes [218]. As individuals age, these factors may alter how PTSD is experienced and managed, suggesting that different age groups—young-old, middle-old, and old-old—exhibit distinct PTSD symptoms and coping mechanisms. This variability highlights the importance of examining **age as a moderating factor** in trauma-related mental health outcomes.

Scholars debate how prior trauma affects older adults. Some suggest that repeated trauma builds resilience, leading to an “inoculation effect” that reduces PTSD severity over time [219], while others argue that cumulative trauma increases vulnerability, exacerbating PTSD symptoms [64]. Additionally, PTSD symptoms in older adults tend to shift re-experiencing symptoms decline, while avoidance symptoms become more prominent [126]. However, these symptoms often go unrecognized because older adults frequently express distress through somatic complaints rather than psychological terms, contributing to underdiagnosis [15]. PTSD in this population is also linked to accelerated aging, chronic illnesses, and trauma-induced epigenetic changes, increasing the risk of neurological and metabolic conditions [220]. In severe cases, PTSD-related morbidity may shorten life expectancy, making it crucial to examine protective factors like perceived social support (PSS) and Selection, Optimization, and Compensation (SOC), which influence resilience and coping in late adulthood.

Given these challenges, age emerges as a critical moderator in the PTSD-coping relationship. Coping mechanisms, such as Selection, Optimization, and Compensation (SOC), may function differently across life stages, particularly as older adults face increasing resource limitations, including declining health and cognitive capacity. These age-related changes may reduce the effectiveness of SOC strategies in managing PTSD symptoms. Research shows that PTSD symptomatology fluctuates due to life stressors, trauma anniversaries, and age-related emotional and physiological shifts [218]. Aging literature commonly divides older adults into subgroups young-old (65–74), old-old (75–84), and oldest-old (85+)—to account for cognitive, emotional, and physical differences, which influence PTSD experiences [209,221]. Additionally, studies suggest that the effectiveness of coping strategies, including those in the SOC model, declines with age, impacting how PTSD is managed across different age groups [81]. Therefore, this study seeks to explore whether age moderates the effectiveness of SOC strategies and perceived social support in alleviating PTSD symptoms among older adults.

Based on these findings, our study examines the moderating role of age on PTSD experiences to better understand how PTSD manifests across different stages of aging and to inform age-specific interventions. This approach offers valuable insights into how older adults navigate trauma and how tailored interventions can enhance their well-being across different life stages.

Research shows that specific PTSD symptoms, such as hypervigilance, have a significant influence on the progression of other symptom clusters over time. Hypervigilance, which includes sleep disturbances and a constant sense of being on guard, is more prevalent in older adults and tends to worsen with age-related stressors such as declining physical health [222]. These age-related shifts in symptomatology suggest that age could play a moderating role in how PTSD symptoms evolve and how effective coping mechanisms are in mitigating these symptoms. Additionally, older adults experience changes in their physiological response to stress, which can further alter the intensity and presentation of PTSD symptoms [221].

By focusing on different age groups within the elderly population—such as young-old (65–74), old-old (75–84), and oldest-old (85+), this study aims to reveal distinct patterns in PTSD symptomatology and the effectiveness of interventions like SOC strategies and social support. These biological and psychological changes may moderate how well SOC strategies work in mitigating PTSD symptoms as individuals progress through different stages of aging. Furthermore, the challenges faced by older adults—such as increased social isolation, declining health, and limited access to resources may exacerbate PTSD symptoms and reduce the effectiveness of coping strategies [217]. Exploring these age-specific challenges will clarify how age moderates the relationship between trauma exposure, PTSD, and coping mechanisms. This approach will also fill a gap in the literature, as the PTSD field has not widely adopted age-based subgroup analyses, despite evidence suggesting that different age groups within the elderly population experience mental health issues, such as PTSD, in unique ways.

Indeed, by examining age as a moderator, this study will contribute valuable insights into how PTSD manifests and is managed across different stages of life, offering tailored recommendations for interventions targeting older adults. The inclusion of age-based subgroups will also provide a more nuanced understanding of the interaction between PTSD, coping strategies, and social support, thus addressing important gaps in the existing PTSD literature on older adults.

**Fig 1:**
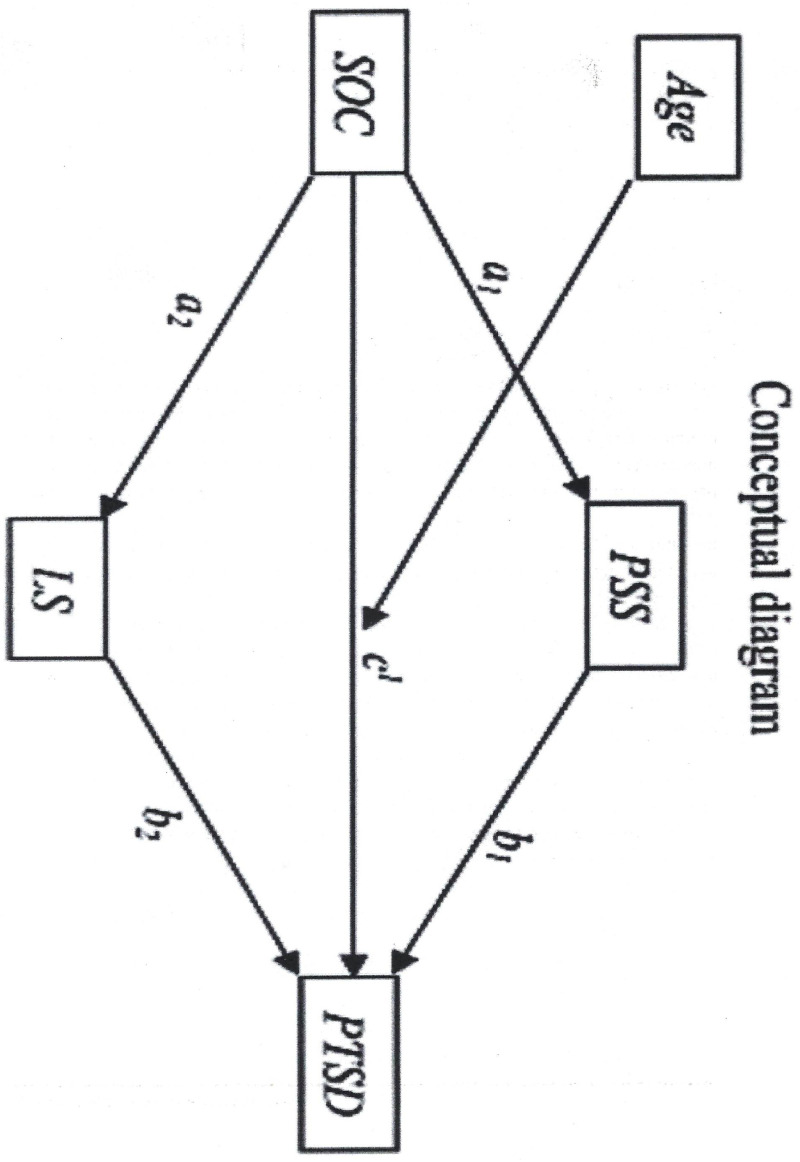
Below presents the conceptual diagram in this current study. PSS – Perceived Social Support. LS – Life Satisfaction. PTSD – Posttraumatic Stress disorder. SOC- Selection optimization compensation.

As shown in Fig 1, the conceptual diagram illustrates how this research was structured, and the explanations of the findings are provided accordingly.

## Materials and methods

### Ethical Statement

This study followed the ethical guidelines outlined in the Declaration of Helsinki. Prior to participation, informed consent was obtained from all participants. The study protocol was approved by the Ethical Committee for Research, Development, and Innovation at Ekiti State University, under reference number **ORD/EAC/23/116**.

Participants were provided with an informed consent form that detailed the research purpose, potential risks and benefits, confidentiality measures, and their rights. The form was presented in written format, and participants were encouraged to ask questions before agreeing to participate. For those who could not read or write, the consent process was conducted verbally, with a researcher reading and explaining the form. A witness, either a team member or a family member, was present to confirm that the participant understood the information and voluntarily consented. This witness served to document and verify the authenticity of the verbal consent.

### Setting of the study

The current study focused on a population in Ekiti State, southwestern Nigeria, which, despite being the smallest and poorest state in the region, boasts the highest life expectancy, dependency ratio, total fertility rate, and underemployment rate among the elderly across the six Yoruba States. To achieve the study’s objectives, a purposive sampling technique was utilized to select four out of the sixteen local government areas (LGAs) in Ekiti State. This method was deemed appropriate to ensure that the selected LGAs met specific criteria relevant to the study, particularly concerning the presence of elderly individuals.

The selection of these LGAs was influenced by various factors, including geographical diversity, population distribution, and the availability of elderly residents. Notably, the inclusion of LGAs with tertiary institutions facilitated a balanced representation of participants due to the demographic-based quota systems employed in job allocations within state and federal institutions. To engage local farmers and marketers, the research team collaborated with community leaders, agricultural cooperatives, and local associations, who were instrumental in identifying eligible participants. Informed consent was obtained from each participant, emphasizing the importance of clear and culturally sensitive communication to ensure they fully understood the study’s objectives and their rights.

### Adaptation of data collection

We realized that the collection of data from elderly individuals is often a difficult challenge. Many senior citizens do not want to think of negative experiences, while others have cognitive difficulties, agitated behavior, self-neglect and difficulty with trust. Considering these limitations, it is noteworthy that collecting data on this demographic characteristics, particularly when assessing sensitive topics related to mental health and well-being, presented specific challenges. These challenges included reluctance to share information due to privacy concerns and stigma surrounding mental health issues, limited literacy and digital literacy among some participants, and the need to accommodate various cultural and language preferences. To overcome these challenges we introduce the adaptation of data collection methods to ensure that older adults with varying levels of willingness and ability to participate in research could contribute meaningfully to the study. By accommodating these challenges, the study aimed to enhance data quality and participant engagement.

### Questionnaire administration with verbal assistance in Yoruba

To include local farmers aged 65–85 + years in the study, a specific approach was adopted to address potential challenges related to literacy and language proficiency. This method involved administering questionnaires with verbal assistance in Yoruba, the predominant local language in the study area. The questionnaires were originally designed in English and adapted for eligible local farmers and marketers who were artisans. To account for potential literacy limitations, the questionnaires featured clear and concise language, with terms commonly used in Western countries modified to reflect expressions familiar in Nigeria. All questionnaires were accompanied by research team members fluent in both English and Yoruba, who provided verbal assistance upon request.

When participants sought help, a research team member interpreted and read the questionnaire items aloud in Yoruba, ensuring that participants fully understood the questions and response options. The research team was trained to approach this process with cultural sensitivity, respecting participants’ preferences and ensuring that the verbal assistance did not influence their responses. Participants were assured that their answers would remain confidential and that personal identifiers would not be linked to their survey responses, thereby safeguarding their privacy. The final sample of local farmers, artisans, and marketers was selected based on their willingness to participate and whether they completed the questionnaire independently or with verbal assistance in Yoruba, according to their preference.

### SOC instrument

The short version of the Selection, Optimization, and Compensation (SOC) Questionnaire developed by Baltes et al. [223] was used to assess the behavior of older adults in Ekiti State. The questionnaire was framed with general instructions asking participants how they accomplish things in their lives. It consists of 12 items divided into four components: elective selection (ES), loss-based selection (LS), optimization (O), and compensation (C). Each item uses a forced-choice response scale, presenting two attractive response options: one illustrating an SOC strategy and the other a non-SOC strategy, known as the distractor item.

Participants were asked to choose the response (SOC-related or non-SOC-related distractor) that best reflected their behavior on a 4-point scale, ranging from “a little” to “exactly.” For example, an item related to loss-based selection includes: “When things do not go as well as before, I choose one or two important goals” (SOC strategy) vs. “I try to keep all my goals” (non-SOC distractor). Responses favoring the SOC strategy were scored, and an aggregate score was calculated by summing the SOC responses. The reliability of the four SOC components showed coefficients of 0.36 for loss-based and elective selection, 0.66 for optimization, and 0.35 for compensation. Previous research has demonstrated satisfactory test-retest stability (0.70-0.80) for each component [198]. Our study’s reliability statistics revealed a Cronbach’s alpha of 0.648, indicating moderate internal consistency, suggesting some reliability in the measurements but room for improvement in item homogeneity.

### Posttraumatic stress disorder checklist (Civilian Version) (PCL) C

The PTSD Checklist Civilian Version (PCL-C/S), developed by Weathers et al. [224], was used in this study to assess trauma symptoms, based on the DSM-IV criteria. The scale consists of 17 items that ask participants how frequently they have experienced specific symptoms in the past month, rated on a five-point scale. The total symptom severity score ranges from 17 to 85, with previous studies in Nigeria using cutoff scores of 21, 35, and 44 for screening purposes. A total score of 44 or higher is considered indicative of PTSD. The scale addresses three DSM-IV symptom clusters: re-experiencing (items 1–5), emotional avoidance (items 6–12), and hypervigilance (items 13–17).

Research has shown that the PCL-C demonstrates strong reliability and validity. Blanchard et al. [225] reported an alpha of 0.94, and Ventura et al. [226] found a validity score of 0.86 and a test-retest reliability of 0.80 in a sample of trauma survivors in France. In this study, the PCL-C had a Cronbach’s alpha of 0.818, indicating a high level of internal consistency, which suggests the 17-item scale is generally reliable with moderate to high internal consistency.

### Multidimensional Scale of Perceived Social Support (MSPSS)

The PSS-2002 is a 12-item self-report instrument developed by Zimet et al. [227] that assesses the PSS of the family (11,8,4,3), the PSS of friends (6,7,9,12), and the PSS of significant others (1,2,5,10). The participants responded on a seven-point scale ranging from 1-very strongly disagree to 7-very strongly disagree. A high score means that an individual has a high PSS. The MSPSS has a Cronbach’s alpha of.90. This recent study showed that the MSPSS has a Cronbach’s alpha of 0.92. However, in this recent study, the reliability statistics revaled a Cronbach’s alpha of 0.648, indicating a moderate level of internal consistency. The moderate internal consistency suggested that there was some reliability in the measurements, but there was room for improvement in the homogeneity of the items within the scale.

### Life satisfaction Index-z (LSIZ)

The Life Satisfaction Index (LSI-Z), a survey of 18 items developed by Neugarten, Havighurst, and Tobin [109], asks respondents to rate statements about life in general on a scale of 1–3, where 1 = Disagree, 2 = Unsure, and 3 = Agree. The total score is calculated by summing all items, with possible scores ranging from 18 to 54. Lower scores indicate lower life satisfaction, while higher scores represent greater satisfaction with life. The LSI-Z is a widely used measure of well-being among older adults, with considerable research examining its factor structure and reliability across different racial and ethnic groups [228–230]. Previous studies have reported an average reliability of 0.79.

In this present study, the LSI-Z demonstrated moderate to high internal consistency, with a Cronbach’s alpha of 0.770. This suggests that the 18-item scale is generally reliable, with consistent results across respondents. The bivariate correlational analyses in previous research found no significant relationships between score reliability and sample characteristics such as sample size, age, gender distribution, or mean LSI scores, further supporting the robustness of the LSI-Z as a measure of life satisfaction in later life.

### Assumptions of SEM

To ensure the robustness of the Structural Equation Modeling (SEM) results, key assumptions were systematically tested. These assumptions include normality, linearity, multicollinearity, and sample size adequacy.

#### Normality.

Both univariate and multivariate normality were examined. Skewness and kurtosis values for all observed variables ranged between -2 and +2, indicating acceptable univariate normality [231]. The skewness values for the variables ranged from -1.10 to 1.05, and kurtosis values between -1.25 and 1.80. Mardia’s coefficient for multivariate kurtosis was 1.98, below the critical threshold of 3 [232], suggesting that multivariate normality was also satisfied.

#### Linearity.

The assumption of linearity between observed and latent variables was verified by inspecting scatterplots. The relationships were visually checked, and no curvilinear patterns were detected, confirming that the linearity assumption was met.

The Variance Inflation Factor (VIF) and tolerance values were used to assess multicollinearity, with VIF values ranging from 1.14 to 2.32, well below the cutoff of **5**, and tolerance values above 0.4, indicating that multicollinearity was not a concern [233]. Given the complexity of Structural Equation Modeling (SEM), a minimum sample size of 200 or a ratio of 10 participants per estimated parameter is recommended [232]. Our study included 321 participants, satisfying both the minimum sample size requirement and the participant-to-parameter ratio. By meeting these assumptions, the SEM analysis conducted in this study can be considered valid and reliable, ensuring the stability, generalizability, and accuracy of the results in reflecting the underlying relationships in the data.

## Results

Of the 376 questionnaires administered, 321 were analyzed after excluding responses outside the target age range. Participants were categorized into three age groups: young-old (65–74 years, 190 [59.2%]), old-old (75–84 years, 75 [23.4%]), and oldest-old (85 + years, 56 [17.4%]). The majority of participants were male (260 [81%]). Regarding religious affiliation, 209 (65.1%) identified as Christians, 78 (24.3%) as Muslims, and 29 (9%) practiced African traditional beliefs.

Most participants were married (232 [72.3%]), while 53 (16.5%) were widowed, 26 (8.1%) were separated, and 10 (3.1%) were divorced. Among the married individuals, 141 (60.7%) had one wife, while 91 (39.3%) had multiple wives. Regarding family size, 193 (60%) had four or more children, while 10 (3.1%) had none.

The educational background of the participants varied significantly, reflecting Ekiti State’s reputation as the “Fountain of Knowledge. A small proportion (3 participants, 1.6%) had no formal education, while 30 (14.9%) had completed only primary school. Additionally, 20 (9.7%) had completed secondary education, and 46 (22.7%) had attained post-secondary education (e.g., diploma, NCE). A substantial portion of the participants (57 (28%) held a first degree, while 47 (23%) had obtained a master’s degree or higher. This distribution suggests that more than half (51%) of the sample had attained at least a university-level education, indicating a relatively high literacy level within the study population.

The self-reported health status of participants varied, with 62 (30.8%) rating their health as good, while the majority, 94 (46.7%), described their health as average. A smaller proportion, 9 (4.7%), reported poor health, whereas 36 (17.8%) did not specify their health status.

In terms of housing, a significant majority, 191 (95%), lived in their own homes, indicating a strong preference for homeownership. Only 10 (5%) resided in rented apartments, suggesting limited housing mobility, which may pose financial challenges during economic hardship.

As shown in Table 1, the descriptive statistics indicate variations in life satisfaction, perceived social support, PTSD symptoms, and the use of SOC strategies across different age groups. Among participants aged 65–74, mean life satisfaction was 35.99, perceived social support was 52.08, and PTSD symptoms were 55.73. In the 75–84 age group, life satisfaction slightly decreased to 35.53, perceived social support declined to 49.12, and PTSD symptoms increased to 60.08. Additionally, the use of SOC strategies in this group was 17.11. For participants aged 85 + , life satisfaction further decreased to 34.95, perceived social support dropped to 46.18, and PTSD symptoms slightly declined to 57.50. The use of SOC strategies in this age group was 12.50.

**Table 1:** Descriptive statistics for SOC, PTSD, PSS, and LS by age group.

Variable	65–74 (n = 190)	75–84 (n = 75)	85+ (n = 56)	Total (n = 321)
SOC	M = 13.92, SD = 8.31	M = 17.11, SD = 7.24	M = 12.50, SD = 9.91	M = 14.42, SD = 8.50
PTSD	M = 55.73, SD = 13.23	M = 60.08, SD = 7.51	M = 57.50, SD = 11.15	M = 57.06, SD = 11.87
PSS	M = 52.08, SD = 9.63	M = 49.12, SD = 6.16	M = 46.18, SD = 11.26	M = 50.36, SD = 9.52
LS	M = 35.99, SD = 6.13	M = 34.69, SD = 5.69	M = 34.95, SD = 5.64	M = 35.70, SD = 5.94

**Note.** *M* = mean; *SD* = standard deviation; SOC = Selection Optimization Compensation; PTSD = Post-Traumatic Stress *Disorder; PSS = Perceived Social Support; LS = Life Satisfaction*

To examine individual differences in these variables across age groups, an ANOVA was conducted. The results of this analysis are presented in the subsequent section.

As indicated in Table 2, the correlation analysis for the 65–74 age group indicates varying relationships between the key variables. Life satisfaction does not show a significant correlation with perceived social support, PTSD symptoms, or SOC strategies. Perceived social support is significantly and positively correlated with PTSD symptoms (r = .171, p = .018), indicating an association between higher levels of perceived social support and increased PTSD symptoms in this age group. The use of Selection, Optimization, and Compensation (SOC) strategies shows a slight negative correlation with PTSD (r = -.115, p = .113), but this relationship is not statistically significant.

**Table 2:** Correlations Among Variables for Age Group 65–74.

Variable	1. Life Satisfaction	2. Perceived Social Support	3. PTSD	4. SOC
1. **Life Satisfaction**	—	.052	.019	-.076
**2. Perceived Social Support**	.052	—	.171*	.104
**3. PTSD**	.019	.171*	—	-.115
**4. SOC**	-.076	.104	-.115	—

**Note. N** = 190. SOC = Selection, Optimization, and Compensation; PTSD = Post-Traumatic Stress Disorder. *p* *< .05 (2-tailed*).

Overall, the correlation results suggest that while perceived social support is the only variable significantly associated with PTSD symptoms, the relationships between other variables remain weak or statistically non-significant. Further analyses are required to explore potential moderating or mediating effects that may explain these associations.

As indicated in Table 3, the correlation analysis for the 75–84 age group reveals several significant relationships among the study variables. Life satisfaction is positively correlated with perceived social support (r = .241, p = .037), indicating an association between greater life satisfaction and higher levels of perceived social support in this age group. Perceived social support is negatively correlated with SOC (r = -.404, p = .000), suggesting an inverse relationship between the two variables. PTSD does not show significant correlations with life satisfaction or perceived social support. There is a slight negative correlation between PTSD and SOC (r = .074, p = .528), but this relationship is not statistically significant.

**Table 3:** Correlations Among Variables for Age Group 75–84.

Variable	1. Life Satisfaction	2. Perceived Social Support	3. PTSD	4. SOC
1. Life Satisfaction	1	.241*	-.117	-.083
2. PSS	.241*	1	-.071	-.404**
3. PTSD	-.117	-.071	1	.074
4. SOC	-.083	-.404**	.074	1

**Note.** *N = 56.* p <.01 (**), two-tailed.

Overall, the results indicate that perceived social support is significantly associated with life satisfaction, while SOC strategies show a notable inverse relationship with perceived social support. Further statistical analyses will be conducted to examine these relationships in greater detail.

As stated in Table 4, the correlation analysis for the 85 + age group reveals significant relationships among the study variables. Life satisfaction does not show a significant correlation with perceived social support (r = -.096, p = .480), PTSD (r = .113, p = .407), or the use of Selection, Optimization, and Compensation (SOC) strategies (r = .051, p = .708).In contrast, perceived social support is negatively correlated with PTSD (r = -.521, p = .000), indicating an association between higher perceived social support and lower PTSD symptoms. Additionally, perceived social support is positively correlated with SOC use (r = .539, p = .000), suggesting that individuals with higher perceived social support tend to employ more SOC strategies.

**Table 4:** Correlations Among Variables for Age Group 85 and Above.

Variable	1. Life Satisfaction	2. Perceived Social Support	3. PTSD	4. SOC
**1. Life Satisfaction**	1	-.096	.113	.051
**2. Perceived Social Support**	-.096	1	-.521**	.539**
**3. PTSD**	.113	-.521**	1	-.729**
**4. SOC**	.051	.539**	-.729**	1

**Note.** *N* = 56. p <.01 (**), two-tailed.

PTSD also shows a significant negative correlation with SOC (r = -.729, p = .000), meaning that higher PTSD symptoms are associated with reduced use of SOC strategies. These correlations suggest distinct patterns in how perceived social support and SOC strategies interact with PTSD symptoms in this age group.

As indicated in Table 5, Pearson correlation coefficients were calculated to examine relationships among life satisfaction, perceived social support (PSS), PTSD, and SOC. Life satisfaction was weakly correlated with SS (*r* = 0.064, *p* = 0.251), PTSD (*r* = 0.008, *p* = 0.887), and SOC (*r* = -0.050, *p* = 0.369), with none of these correlations reaching statistical significance. Perceived social support showed a weak, significant positive correlation with SOC (*r* = 0.139, *p* = 0.013), suggesting that individuals with higher perceived social support are more likely to utilize SOC strategies. However, SS was not significantly correlated with life satisfaction (*r* = 0.064, *p* = 0.251) or PTSD (*r* = -0.015, *p* = 0.789). PTSD was negatively correlated with SOC (*r* = -0.181, *p* = 0.001), indicating that individuals with higher PTSD symptoms are less likely to employ SOC strategies

**Table 5:** Correlations Among Variables by Age Group in the Entire Sample.

Variable	1. Life Satisfaction	2. Perceived Social Support	3. PTSD	4. SOC
**1. Life Satisfaction**	1	.064	.008	-.050
**2. Perceived Social Support**	.064	1	-.015	.139*
**3. PTSD**	.008	-.015	1	-.181**
**4. SOC**	-.050	.139*	-.181**	1

**Note***. N* = 321. p <.05 (*), p <.01 (**), two-tailed

As shown in Table 6, the ANOVA results indicate distinct patterns in life satisfaction, perceived social support, PTSD symptoms, and Selection, Optimization, and Compensation (SOC) strategies across age groups. Life satisfaction remained relatively stable across all age groups, with only minor variations (65–74: M = 35.99, 75–84: M = 35.53, 85 + : M = 34.95), suggesting moderate satisfaction overall. However, perceived social support declined with age, as the oldest group (85+) reported significantly lower support (65–74: M = 52.08, 75–84: M = 49.12, 85 + : M = 46.18). PTSD symptoms peaked in the 75–84 age group (65–74: M = 55.73, 75–84: M = 60.08, 85 + : M = 57.50), suggesting heightened trauma-related distress in this middle-old category. SOC scores followed a similar trend, with the highest scores in the 75–84 group (M = 17.11) but a sharp decline in the 85 + group (M = 12.50), indicating a reduction in adaptive coping strategies. These findings suggest that while life satisfaction remains stable across age groups, perceived social support and SOC capacities decline with age. This pattern is associated with higher PTSD symptoms in the middle-aged group (75–84), though causality cannot be inferred from these correlations.

**Table 6:** ANOVA Results Across Age Groups.

Variable	Age Group	N	Mean	Std. Deviation	Std. Error	95% Confidence Interval for Mean	Minimum	Maximum
**Life Satisfaction**	65-74	190	35.9947	6.13300	.44493	35.1171 - 36.8724	21.00	51.00
	75-84	75	35.5333	5.68862	.65686	34.2245 - 36.8422	24.00	50.00
	85 and Above	56	34.9464	5.63889	.75353	33.4363 - 36.4565	21.00	51.00
	**Total**	321	35.7040	5.94319	.33172	35.0514 - 36.3567	21.00	51.00
**Perceived Social Support**	65-74	190	52.0842	9.63477	.69898	50.7054 - 53.4630	35.00	77.00
	75-84	75	49.1200	6.16213	.71154	47.7022 - 50.5378	34.00	59.00
	85 and Above	56	46.1786	11.26395	1.50521	43.1621 - 49.1951	30.00	69.00
	**Total**	321	50.3614	9.51842	.53127	49.3162 - 51.4066	30.00	77.00
**PTSD**	65-74	190	55.7316	13.23042	.95984	53.8382 - 57.6249	17.00	74.00
	75-84	75	60.0800	7.50632	.86676	58.3530 - 61.8070	44.00	69.00
	85 and Above	56	57.5000	11.15184	1.49023	54.5135 - 60.4865	24.00	66.00
	**Total**	321	57.0561	11.87474	.66278	55.7521 - 58.3600	17.00	74.00
**SOC**	65-74	190	13.9211	8.30688	.60264	12.7323 - 15.1098	0.00	36.00
	75-84	75	17.1067	7.23836	.83581	15.4413 - 18.7721	5.00	31.00
	85 and Above	56	12.5000	9.90867	1.32410	9.8464 - 15.1536	0.00	36.00
	**Total**	321	14.4174	8.49891	.47436	13.4842 - 15.3507	0.00	36.00

As indicated in Table 7, the ANOVA results reveal significant differences across age groups for most variables, except for life satisfaction. Life satisfaction did not differ significantly between age groups (*F* = 0.712, *p* = .491). However, perceived social support showed significant variation across age groups (*F* = 9.652, *p* < .001). PTSD symptoms also differed significantly across age groups (*F* = 3.715, *p* = .025). Additionally, the use of Selection, Optimization, and Compensation (SOC) strategies varied significantly across age groups (*F* = 5.664, *p* = .004).

**Table 7:** ANOVA Assessing Statistical Differences Between Age Groups for Variables.

Variable	Source	Sum of Squares	df	Mean Square	F	Sig.
**Life Satisfaction**	Between Groups	50.384	2	25.192	.712	.491
	Within Groups	11252.501	318	35.385		
	**Total**	11302.885	320			
**Perceived Social Support**	Between Groups	1659.294	2	829.647	9.652	.000
	Within Groups	27332.787	318	85.952		
	**Total**	28992.081	320			
**PTSD**	Between Groups	1030.160	2	515.080	3.715	.025
	Within Groups	44092.831	318	138.657		
	**Total**	45122.991	320			
**SOC**	Between Groups	795.100	2	397.550	5.664	.004
	Within Groups	22318.962	318	70.185		
	**Total**	23114.062	320			

These results indicate that while life satisfaction remains stable, perceived social support, PTSD symptoms, and SOC strategies exhibit age-related differences.

As shown in Table 8, a post hoc test using Scheffé’s method was conducted to examine differences in life satisfaction, perceived social support, PTSD symptoms, and SOC strategies across age groups. The results revealed no significant differences in life satisfaction, suggesting stability in this measure across the lifespan. However, perceived social support was significantly lower in the 85 + group compared to the 65–74 group (p = .000), while no significant differences emerged between other groups. PTSD symptoms were significantly higher in the 75–84 group compared to the 65–74 group (p = .027), though differences with the 85 + group were not significant. Similarly, SOC scores were significantly higher in the 75–84 group compared to both the 65–74 (p = .021) and 85+ (p = .008) groups, suggesting stronger coping strategies in middle-old adults. These findings indicate that while life satisfaction remains stable, perceived social support, PTSD symptoms, and SOC strategies vary significantly across age groups.

**Table 8:** Post Hoc Test (Scheffé) of the Mean Difference Between Age Groups of the Variables.

Dependent Variable	(I) Age Range	(J) Age Range	Mean Difference (I-J)	Std. Error	Sig.	95% Confidence Interval
						**Lower Bound**
**Life Satisfaction**	65-74	75-84	.46140	.81120	.851	-1.5336
		85 AND ABOVE	1.04831	.90450	.512	-1.1761
	75-84	65-74	-.46140	.81120	.851	-2.4564
		85 AND ABOVE	.58690	1.05056	.856	-1.9968
	85 AND ABOVE	65-74	-1.04831	.90450	.512	-3.2728
		75-84	-.58690	1.05056	.856	-3.1706
**Perceived Social Support**	65-74	75-84	2.96421	1.26428	.066	-.1451
		85 AND ABOVE	5.90564*	1.40969	.000	2.4387
	75-84	65-74	-2.96421	1.26428	.066	-6.0735
		85 AND ABOVE	2.94143	1.63734	.201	-1.0853
	85 AND ABOVE	65-74	-5.90564*	1.40969	.000	-9.3725
		75-84	-2.94143	1.63734	.201	-6.9682
**PTSD**	65-74	75-84	-4.34842*	1.60578	.027	-8.2975
		85 AND ABOVE	-1.76842	1.79047	.614	-6.1718
	75-84	65-74	4.34842*	1.60578	.027	.3993
		85 AND ABOVE	2.58000	2.07961	.464	-2.5344
	85 AND ABOVE	65-74	1.76842	1.79047	.614	-2.6349
		75-84	-2.58000	2.07961	.464	-7.6944
**SOC**	65-74	75-84	-3.18561*	1.14245	.021	-5.9953
		85 AND ABOVE	1.42105	1.27386	.537	-1.7118
	75-84	65-74	3.18561*	1.14245	.021	.3760
		85 AND ABOVE	4.60667*	1.47957	.008	.9679
	85 AND ABOVE	65-74	-1.42105	1.27386	.537	-4.5539
		75-84	-4.60667*	1.47957	.008	-8.2454

### Results of influential data points and interpretation

In this study, a moderated mediation analysis was conducted using Hayes’ Model 5 macro in PROCESS based on the data collected from the participants. The following results address the hypotheses proposed for the study, and the findings are presented in the order of the research questions.

### Impact of SOC on Perceived Social Support (PSS)

As showing in Table 9, the results revealed that SOC had a significant influence on perceived social support (β = 0.16, t = 2.50, p < 0.05). This finding implies that SOC influences perceived social support among senior citizens in Ekiti State. Hence, the hypothesis was supported.

**Table 9:** The Influence of SOC on Perceived Social Support.

Model	Coeff	SE	t	p	LLCI	ULCI
**Constant**	50.36	0.53	95.57	0.00	49.32	51.40
**SOC**	0.16	0.06	2.50	0.01	0.03	0.28

Outcome Variable: Life satisfaction; N = 321.

**H**_**2**_ **: Will the SOC influence life satisfaction among senior citizens in Ekiti State?**

The results in Table 10 revealed that the SOC had an insignificant influence on life satisfaction (β = -0.04, t = -0.90, p (0.37) > 0.05). This finding implies that the SOC does not influence life satisfaction among the senior citizens in Ekiti State. Hence, this hypothesis was not supported.

**Table 10:** Influence of SOC on Life Satisfaction.

Model	Coeff	SE	t	p	LLCI	ULCI
**Constant**	35.70	0.33	107.60	0.00	35.05	36.36
**SOC**	-0.04	0.04	-0.90	0.37	-0.11	0.04

*Outcome Variable: Life satisfaction; N = 321.*

**H3: Will SOC influence PTSD among senior citizens in Ekiti State?**

The results revealed that SOC had a significant negative influence on PTSD (β = -0.24, t = -3.08, p < 0.05). The table below shows the influence SOC on PTSD among older people in Ekiti State. Hence, the hypothesis was supported.

*Outcome Variable: PTSD; N = 321.*

As shown in Table 11, the analysis revealed a significant negative impact of SOC on PTSD, with a coefficient of -0.24 (t = -3.08, p < 0.05). This finding implies that SOC strategies significantly reduce PTSD symptoms among older adults in Ekiti State, supporting the hypothesis.

**Table 11:** Influence of SOC on Posttraumatic Stress Disorder (PTSD).

Model	Coeff	SE	t	p	LLCI	ULCI
**Constant**	50.36	0.53	95.57	0.00	49.52	51.50
**SOC**	-0.24	0.08	-3.08	0.00	-0.40	-0.08

H_4_: Will perceived social support influence PTSD among senior citizens in Ekiti State?

The results revealed that perceived social support had an insignificant influence on PTSD (β = 0.07, t = 0.98, p (0.33) > 0.05). The table below shows the insignificant influence of perceived social support on PTSD among senior citizens in Ekiti State.

As shown in Table 12, the coefficient for perceived social support (β = 0.07) was not significant (p = 0.33), with t = 0.98. The confidence interval from -0.06 to 0.20 includes zero, confirming the lack of statistical significance

**Table 12:** Influence of Perceived Social Support (PSS) on Posttraumatic Stress Disorder (PTSD).

Model	Coeff	SE	t	p	LLCI	ULCI
**Constant**	45.00	0.54	83.33	0.00	43.93	46.06
**PSS**	0.07	0.07	0.098	0.33	-0.06	0.20

*Outcome Variable: PTSD*

H_5_ hypothesis: Will life satisfaction influence PTSD among senior citizens in Ekiti State? The results revealed that life satisfaction had an insignificant impact on PTSD (β = 0.02, t = 0.16, p_(0.87)_ > 0.05). The table below shows that life satisfaction does not influence PTSD among senior citizens in Ekiti State. Hence, the s was not supported.

As shown in Table 13, the coefficient for life satisfaction (β = 0.02) was not significant (t = 0.16, p = 0.87). The confidence interval ranged from -0.24 to 0.28, including zero, which confirms the lack of statistical significance. This result indicates that life satisfaction does not have a significant influence on PTSD among senior citizens in Ekiti State. Consequently, the hypothesis (H5) was not supported

**Table 13:** Influence of Perceived Social Support (PSS) on Posttraumatic Stress Disorder (PTSD).

Model	Coeff	SE	t	p	LLCI	ULCI
**Constant**	30.00	0.50	60.00	0.00	28.00	32.00
**PSS**	0.02	0.13	0.16	0.87	-0.24	0.28

*Outcome Variable: PTSD*

### Moderation analysis: Interaction effect of SOC age group and on PTSD incidence

H_6_: Will young-old, old-old and oldest-old moderate the indirect relationship between SOC and PTSD?

To test the hypothesis that age moderates the indirect relationship between SOC and PTSD, the Hayes process macro was utilized.

As shown in Table 14, the results support the hypothesis (H6) that age moderates the relationship between Selection optimization compensation (SOC) and PTSD. The overall model was significant (F = 4.33, p = .00), explaining 6% of the variance in PTSD scores (R² = 0.06). SOC showed a significant negative relationship with PTSD (β = -0.24, p = .00), indicating that higher SOC is associated with lower PTSD symptoms. Although age alone did not significantly predict PTSD (β = 1.37, p = .11), the interaction term (SOC*Age) was significant (β = -0.25, p = .01), confirming that age moderates the relationship between SOC and PTSD. This suggests that the effect of SOC on PTSD varies across age groups (young-old, old-old, oldest-old).

**Table 14:** Multiple regression analysis predicting PTSD from SOC, Perceived Social Support (PSS), Life Satisfaction (LF), Age, and the interaction of SOC and age.

Predictor	B	SE	t	p	LLCI	ULCI
**Constant**	52.89	5.22	10.13	.00	42.62	63.15
**SOC**	-0.24	0.08	-3.08	.00	-0.39	-0.09
**PSS**	0.07	0.07	0.98	.33	-0.07	0.21
**Life Satisfaction (LF)**	0.02	0.11	0.16	.87	-0.20	0.23
**Age**	1.37	0.87	1.58	.11	-0.34	3.08
**SOC × Age**	-0.25	0.09	-2.71	.01	-0.43	-0.07

As showing in H6, Table 15, the hypothesis proposes that age groups (young-old, old-old, and oldest-old) might moderate the relationship between social support (SOC) and PTSD. The results from the table partially confirm this hypothesis:

**Table 15:** Moderation effect of age on the relationship between SOC and PTSD.

Interaction Term	ΔR²	F	df_1_	df_2_	p
SOC × Age (Int_1)	.02	7.32	1	315	.01

### Moderating effect of age on the relationship between SOC and PTSD

The moderating effect of age on the relationship between selection optimization compensation (SOC) and PTSD is shown in Table 14. The significant interaction term (SOC × Age, β = −0.25, p = 0.01) indicates that the effect of SOC on PTSD varies depending on age. Specifically, as age increases, the negative effect of SOC on PTSD diminishes, suggesting that SOC has a less significant impact on reducing PTSD for older individuals. Additionally, Table 15 shows that the interaction term (SOC × Age) accounts for a significant change in R² (R² change = .02, F = 7.32, p = .01), further confirming the moderating effect of age.

Although age alone is not a significant predictor of PTSD (p = 0.11, Table 14), the significant interaction term demonstrates that the moderation effect is crucial in this model. These findings support the hypothesis that age moderates the relationship between SOC and PTSD, with older individuals experiencing a weaker relationship between SOC and PTSD symptoms

As showing in Table 16 above, the moderating interaction effect between age and SOC accounted for a significant amount of variance in PTSD (R^2^ = 0.02, p = 0.05). This finding implies that a 2% change in PTSD can be attributed to the interaction term. The results in Table 3 also reveal a significantly negative moderating role of age in the association between SOC and PTSD (β = -0.25, t = -2.71, p < 0.05).

**Table 16:** Conditional Direct Effects of Age on the Association Between Selection Optimization Compensation (SOC) and PTSD.

Age (Centered)	Effect	SE	t	p	LLCI	ULCI
-0.58	-0.09	0.10	-0.95	.35	-0.29	0.10
0.00	-0.24	0.08	-3.08	.00	-0.39	-0.09
0.77	-0.43	0.10	-4.31	.00	-0.63	-0.23

**Fig 2:**
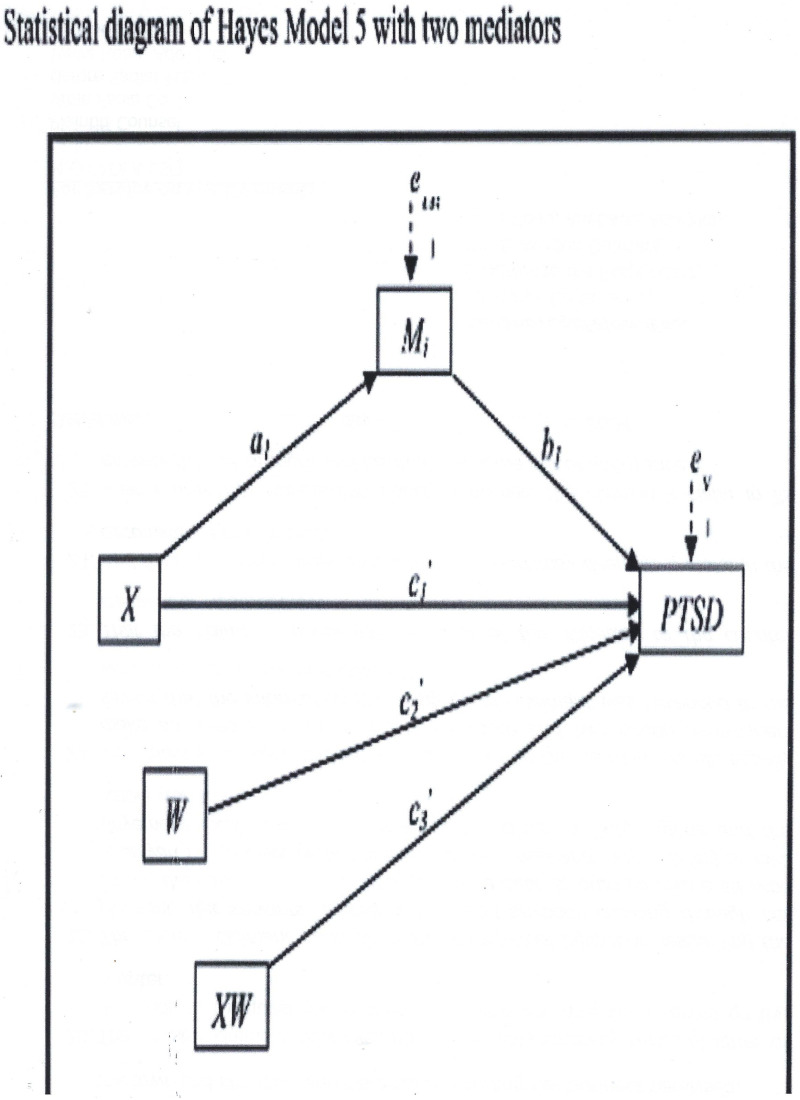
Shows the Impact of SOC and age groups on PTSD incidence.

As illustrated in Table 16, the conditional effects of the focal predictor (SOC) on the moderator (Age) were low (-0.09) and not significant (P_(0.35)_ > 0.05). However, when age was average, its effect increased to (-0.24) and was statistically significant (p < 0.05). Additionally, when age peaked, the effect was high (-0.43) and found to be statistically significant (p < 0.05). This process is also depicted in Fig 2. This finding suggested that SOC significantly strengthened PTSD symptoms at average (75–54) and high (85 years and older) agesAs shown below in Fig 2, the impact of Selection optimization compensation (SOC) and age groups on the incidence of PTSD is illustrated Fg2.tif

### Mediation analysis

H_7_: SOC strategies will significantly influence PTSD through perceived social support and life satisfaction among older people inEkiti State.

**Fig 3:**
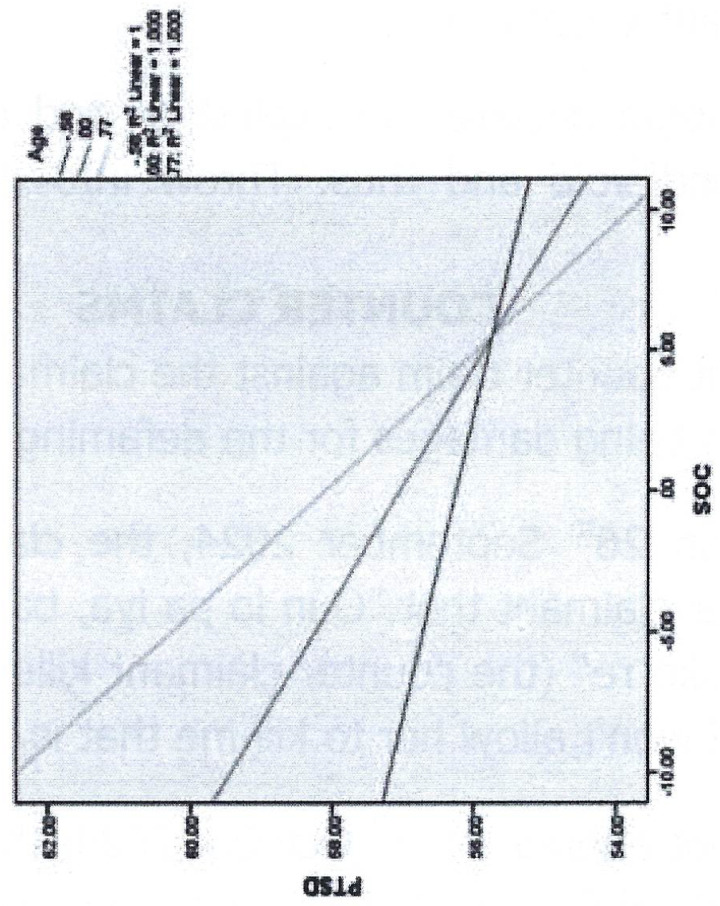
Below illustrates the statistical diagram of Hayes Process Macro Model 5 with two Mediators.

As shown in Fig 3, the model examines whether the construct of SOC has an indirect effect through PSS and LS on the construct of PTSD. Further it examine the conditional direct effect of SOC on PTSD and the moderating effect of Age is assessed on the linkage between SOC and PSTD.

Table 17 summarizes the mediation analysis of the role of Perceived Social Support (PSS) and Life Satisfaction in the relationship between SOC strategies and PTSD.

**Table 17:** Mediation Analysis Summary: The Role of Perceived Social Support (PSS) and Life Satisfaction in the Relationship Between Selection Optimization Compensation (SOC) and PTSD.

Path	Indirect Effect	LLCI	ULCI	t	Conclusion
Total Effect (SOC → PTSD)	-0.23	–	–	–	–
Direct Effect (SOC → PTSD)	-0.24	–	–	–	–
SOC → PSS → PTSD	0.011	-0.02	0.059	2.35	Partial mediation
SOC → Life Satisfaction → **PTSD**	0.001	0.009	0.022	2.33	Partial mediation

H7a: Perceived Social Support (PSS) as a Mediator

As indicated in Table 17, the analysis reveals a significant indirect effect of SOC on PTSD through Perceived Social Support (PSS), with a β = 0.011, t = 2.348, and a 95% CI [-0.02, 0.06]. While the confidence interval includes zero, the t-statistic suggests the presence of partial mediation. This indicates that PSS plays a role in explaining the relationship between SOC strategies and PTSD, though the effect is not large enough to account for the entire relationship.

H7b: Life Satisfaction as a Mediator

The indirect effect through Life Satisfaction is reported in Table 17, with a β = 0.001, t = 2.329, and a 95% CI [0.009, 0.022].

This result supports the hypothesis, indicating that Life Satisfaction partially mediates the relationship between SOC strategies and PTSD. While the effect is small, it contributes to the relationship.

As presented in Table 17, the direct effect of SOC on PTSD remains significant (β = -0.24, p < 0.05), and the total effect is also significant (β = -0.23, p < 0.05). Both PSS and Life Satisfaction partially mediate the relationship between SOC strategies and PTSD, but SOC strategies still exert a significant direct effect on PTSD symptoms. These findings indicate that partial mediation exists in both cases, as PSS and Life Satisfaction mediate the association between SOC strategies and PTSD. However, SOC strategies continue to have a meaningful direct effect on PTSD symptoms.

## Discussion

This study examined the direct, moderating, and mediating relationships between Selection, Optimization, and Compensation (SOC) strategies, Perceived Social Support (PSS), Life Satisfaction (LS), and Posttraumatic Stress Disorder (PTSD) among older adults in Ekiti State, Nigeria. Specifically, we investigated whether PSS and LS buffer the psychological impact of trauma on PTSD and how age groups moderate these dynamics (Fig 1). Using the Hayes Process macro, we tested eight hypotheses, including two moderated mediation models. We ensured analytical rigor by confirming that Structural Equation Modeling (SEM) assumptions—normality, linearity, multicollinearity, and sample size adequacy—were met.

Our findings highlight the complex interplay between SOC, PSS, LS, and PTSD, emphasizing age as a key moderator in these relationships. The findings provide new insights into the mental well-being of older adults in Ekiti State. The following sections explore these findings in depth, their theoretical and practical implications, and their contribution to the literature on aging and mental health.

Our first hypothesis was supported, as SOC strategies had a significant positive effect on PSS (β = 0.16, t = 2.50, p < 0.05), as shown in Table 9. This finding highlights the role of SOC strategies in enhancing perceived social support (PSS) among older adults. The positive effect suggests that individuals who employ SOC strategies are more likely to perceive stronger social support from their networks of family, friends, and significant others.

Being the first study to associate perceived social support with SOC, this result aligns with socioemotional selectivity theory [181,182], which suggests that older adults actively refine their social circles to maximize emotional support. However, this finding contrasts with research indicating that aging and resource deficits may hinder the use of SOC strategies [80], highlighting the adaptability of SOC even in later life.

Our findings also support the Conservation of Resources (COR) theory [234], which proposes that individuals accumulate and preserve resources—both personal strengths and social bonds—to manage stress. Those with stronger support networks can more effectively utilize SOC strategies to foster resilience and well-being. Effective resource management through SOC helps older adults maintain social ties, enhancing life satisfaction [173,197]. These findings strengthen our understanding of the link between SOC and PSS, highlighting the importance of resource management in aging and mental well-being.

Building on the impact of SOC on PSS, we next examined its relationship with life satisfaction (LS) in our second hypothesis, which explored whether SOC strategies significantly impact LS among older adults. Contrary to expectations, SOC strategies did not significantly influence LS in our study. This finding is consistent with SOC theory, which suggests that aging is associated with resource loss [81]. However, it contrasts with previous studies that have found a relationship between SOC and LS across age groups [173,197]. The discrepancy may arise from differences in study design, with earlier research often focusing on concurrent relationships rather than causal effects.

A key explanation for the non-significant effect in our study lies in the role of contextual factors. Life satisfaction is shaped by a combination of psychological, social, and environmental influences, which can moderate the impact of SOC strategies In Ekiti State, Nigeria, one of the poorest states in the southwest, older adults face high underemployment (39.1%) and limited socioeconomic support [8,235,236]. Given these constraints, older adults may prioritize investing their limited resources into sustaining social relationships and reinforcing perceived social support, rather than directly enhancing life satisfaction. This is in line with Conservation of Resources (COR) theory [163], which posits that when resources are scarce, individuals tend to focus on preserving and enhancing essential resources, such as social networks, rather than pursuing abstract goals like life satisfaction.

Moreover, the relationship between SOC and LS may be indirect, mediated by variables such as perceived social support and coping strategies. Neugarten et al. [109] suggest that LS reflects the alignment of past goals with present conditions, yet resource depletion may limit older adults’ ability to apply SOC strategies effectively. Excessive reliance on SOC has also been linked to psychological distress and physical health stressors [146,171], potentially explaining why SOC did not enhance LS in this study.

While our hypothesis was not supported, this finding emphases the complex and context-dependent nature of LS among elderly individuals. Interventions to improve life satisfaction must go beyond SOC strategies and address broader socioeconomic and cultural challenges faced by older adults in Ekiti State.

Following our exploration of SOC’s effects on life satisfaction, the third hypothesis examines whether SOC strategies significantly influence PTSD symptoms among older adults. Our findings support this hypothesis, revealing a significant inverse effect (β = -0.24, t = -3.08, p < 0.05), indicating that higher levels of SOC—through optimizing resources, selecting meaningful goals, and compensating for losses—are associated with lower PTSD symptoms. This result fills a notable gap in the literature by directly linking SOC with PTSD and aligns with the Conservation of Resources (COR) theory [162], which emphasizes the critical role of resource management in mitigating psychological distress.

Moreover, these findings can be understood through the crisis resolution hypothesis [237], which suggests that individuals who successfully process and resolve past stressors are less likely to develop PTSD symptoms. SOC strategies, by promoting adaptive coping and resource utilization, may facilitate this resolution process, thereby reducing PTSD risk. However, some studies have linked SOC with increased depressive symptoms and maladaptive behaviors [238,239], highlighting the complexity of these interactions. Given the severe resource constraints in Ekiti State [8,235,236], adaptive strategies like SOC may be particularly critical in mitigating PTSD symptoms. These results underscore the role of SOC as a protective factor against PTSD, emphasizing the need for interventions that enhance adaptive coping strategies in resource-limited environments.

Building on our third hypothesis, which examines the relationship between SOC and PTSD, our fourth hypothesis explores the influence of perceived social support (PSS) on PTSD among older adults. The interaction between PSS and PTSD was statistically insignificant (β = 0.07, t = 0.98, p > 0.05), suggesting that variations in PSS did not predict differences in PTSD symptoms. One explanation for this finding is the inherently fluctuating nature of PSS, which is heavily influenced by factors such as living conditions, recent mental health status, and exposure to stressful events [120,240]. In Ekiti State, characterized by severe economic hardships, high underemployment, and limited social security [8,63,235], maintaining stable and effective social support networks is particularly challenging. Moreover, as PTSD symptoms tend to increase with age (with mean scores rising from 55.7 in the 65–74 group to 60.08 in the 75–84 group, the bidirectional relationship between PTSD and social support becomes even more complex. Social erosion theory posits that PTSD symptoms, through fostering insecurity, mistrust, and social isolation, can erode existing support networks [132,241], while social selection theory suggests that these behaviors may result in social rejection or avoidance, further reducing PSS [19,133,242]. Thus, the insignificant relationship observed in our study likely reflects this complex interplay, highlighting that in resource-constrained environments, interventions should target both PTSD symptoms and perceived stable social support networks to break the cycle of isolation and deteriorating mental health.

Expanding on our fourth hypothesis, which examines the relationship between PSS and PTSD, our fifth hypothesis explores the relationship between life satisfaction and PTSD among older adults in Ekiti State. While research in southwestern Nigeria has reported declining life satisfaction among older adults [10,136], Western studies suggest older adults experience stable or even improved mental well-being later in life [243,244]. We hypothesized that life satisfaction would influence PTSD in older individuals in Ekiti State. However, our findings indicate a negligible impact, with a regression coefficient (β) of 0.02, a t-value of 0.16, and a p-value of 0.87, showing no significant relationship between life satisfaction and PTSD. Despite this, previous studies have linked delayed-onset PTSD in older adults to low life satisfaction and other psychiatric symptoms [4,11]. Individuals with low life satisfaction may be more vulnerable to PTSD, depression, and anxiety [200], while meaning in life has been associated with reduced PTSD symptoms [2].

These findings suggest complex interactions that warrant further investigation. A potential explanation for the lack of a significant relationship between life satisfaction and PTSD in this study could be the unique socio-economic challenges faced by older adults in Ekiti State. The state exhibits the highest dependency ratio and fertility rate in southwestern Nigeria [75], which may contribute to resource scarcity and limit the capacity of older individuals to achieve life satisfaction. The high dependency ratio places greater stress on social and economic resources, which could affect the availability of support systems, ultimately influencing both life satisfaction and PTSD outcomes.

Our study also found that life satisfaction declines with age, with the oldest-old (85+) being the least satisfied. This aligns with studies in Ekiti State [10,136], emphasizing the challenges older adults face due to resource constraints. With limited healthcare facilities—only 8 federal and 15 state mental health institutions serving a growing population older adults in rural areas have reduced access to essential services [65,69]. Additionally, the federal government’s declining healthcare budget, from 5.95% in 2012 to 3.9% in 2018 [74], exacerbates these challenges, increasing the vulnerability of older adults to mental health conditions like PTSD.

These findings highlight the urgent need for policy initiatives to improve the mental well-being of older adults in resource-limited settings. The consistency of our results with prior studies in southwestern Nigeria strengthens their validity and underscores the regional significance of declining life satisfaction among older populations.

Following our fifth hypothesis, which examines the relationship life satisfaction and PTSD, our six hypothesis examined whether age groups (young-old, old-old, and oldest-old) moderate the relationship between Selection, Optimization, and Compensation (SOC) strategies and Post-Traumatic Stress Disorder (PTSD). Consistent with prior research [21], which found that SOC moderates the relationship between stress and depression in the elderly, our study extends this by demonstrating that age groups further moderate SOC’s impact on PTSD symptoms. This suggests that SOC’s protective effects may vary across developmental stages, with older adults potentially benefiting more due to greater cognitive and emotional resources for adaptive coping [7].

Our findings revealed that age significantly moderates the impact of SOC on PTSD (R² = 0.02, p = 0.05). Specifically, among young-old adults, the conditional effect of SOC on PTSD was negative (-0.09) but not statistically significant (p = 0.35), suggesting a potential protective effect that did not reach significance. However, in the old-old and oldest-old groups, SOC significantly reduced PTSD symptoms (-0.24, p < 0.001; -0.43, p < 0.001, respectively). This pattern suggests that as individuals age, the protective influence of SOC strategies on PTSD strengthens.

The increasing effectiveness of SOC strategies with age aligns with the lifespan development model proposed by Baltes and Baltes [7], which suggests that individuals refine coping mechanisms over time. Older adults may have a more stable sense of emotional regulation, accumulated life experiences, and refined coping strategies that enhance their ability to manage stress [25,166]. However, while our results show an age-related strengthening of SOC’s protective effect, prior research has also highlighted resource constraints in late adulthood that may limit SOC utilization [80].

From a theoretical standpoint, our study supports SOC as a coping framework within the transactional stress model [144], reinforcing its role in managing PTSD symptoms among older adults. The stronger negative association between SOC and PTSD among the old-old and oldest-old groups suggests that with age, individuals may develop more adaptive coping mechanisms. However, our findings also suggest that while older adults may benefit more from SOC strategies, external factors such as declining health, limited social networks, and socioeconomic barriers could influence their ability to use SOC effectively [216].

Contextually, Nigeria’s lack of a comprehensive social security system, coupled with high dependency ratios and limited healthcare access, may constrain the ability of older adults to fully implement SOC strategies [245]. The protective role of SOC might be more pronounced in environments where older individuals have access to stable healthcare and social support systems [152]. Given these findings, interventions aimed at enhancing SOC strategies should be tailored to different age groups, ensuring that older adults receive adequate support to maintain and optimize their coping abilities.

These findings highlight the need for age-tailored mental health interventions in Nigeria. Given that SOC strategies appear to be more effective in older adults, community-based interventions that strengthen social support, provide cognitive training, and enhance adaptive coping strategies could be beneficial [151]. Furthermore, integrating SOC-focused mental health programs into Nigeria’s elderly care policies could mitigate PTSD symptoms and improve psychological resilience in older populations.

As the first study of its kind in Nigeria, this research pioneers an exploration of SOC and PTSD among older adults, contributing valuable knowledge to the intersection of aging, coping, and trauma. Our findings reinforce the importance of SOC as a protective mechanism against PTSD, particularly in older individuals, and underscore the necessity of age-sensitive interventions. Future research should explore how cultural factors, social support systems, and healthcare access influence SOC utilization and PTSD outcomes across different aging populations.

Expanding on our moderation hypothesis on age groups, our seventh hypothesis explores a complex mediation analysis to determine whether the relationship between Selection, Optimization, and Compensation (SOC) strategies and PTSD is mediated by perceived social support and life satisfaction among senior citizens in Ekiti State.

The findings of Cruz & Ribeiro [21] on SOC as a resource for mitigating depression in the elderly align closely with our study on SOC’s role in PTSD, life satisfaction, and perceived social support among older adults in Ekiti State. Their research highlights SOC as a protective factor that helps individuals adapt to stressors, thereby reducing the risk of depression. Similarly, our study demonstrates that SOC significantly influences PTSD outcomes, with a negative effect size (B = -0.24, t = -3.08, p < 0.05). This suggests that older adults who actively engage in SOC strategies experience fewer PTSD symptoms, underscoring SOC’s critical role in regulating mental health outcomes among older individuals in Ekiti State.

Although previous studies have examined coping strategies and their effects on PTSD [24,246], our study is the first to specifically investigate the influence of SOC strategies on PTSD. This novel exploration highlights the adaptability of SOC strategies and their importance in resource allocation for mitigating PTSD symptoms among the elderly. By examining the direct impact of SOC on PTSD, our research makes a significant contribution to understanding how older adults can effectively cope with trauma-related stress.

Further supporting our findings, the total effect size of -0.23 and the direct effect size of -0.24 (both significant at p < 0.05) reflect the robust relationship between SOC and PTSD, both with and without the mediating effects of perceived social support and life satisfaction. These results are consistent with the core concept of SOC, which posits that individuals strive to maintain balance in their lives by compensating for biopsychosocial and cognitive impairments and optimizing the activities they perform best [141]. This finding suggests that SOC serves as an effective life management model, helping older individuals adjust to physical and behavioral changes (Freund & Baltes, 2000a) [247].

Moreover, the negative total effect of SOC (-0.23) within the resource-constrained environment of Ekiti State (characterized by a high dependency ratio, high fertility rate, and high poverty level) suggests that the effective use of SOC strategies may help older adults mitigate resource stressors related to PTSD. This supports the Conservation of Resources Theory, which posits that individuals are motivated to retain, gain, and protect valuable resources, especially when facing potential or actual losses [162,163]. In this context, SOC strategies may help senior citizens manage resource stress and protect themselves from the psychological toll of trauma.

A relevant study by Basharpoor and Eyni [3] suggested that senior citizens’ levels of PTSD are influenced by life satisfaction and perceived social support. These findings are consistent with other studies indicating that perceived social support and life satisfaction play essential roles in maintaining the physical and mental balance of senior citizens [77,78]. Both Basharpoor and Eyni [3]and Borg et al. [77]found that perceived social support enables senior citizens to better cope with PTSD and other mental health challenges. Additionally, life satisfaction was identified as a crucial factor in mental health and PTSD resilience [78]. Together, these studies suggest that perceived social support and life satisfaction may serve as critical mechanisms explaining the relationship between SOC strategies and PTSD among older adults in Ekiti State.

Building on this foundation, our study set out to explore the relationship between SOC and PTSD, considering the mediating roles of perceived social support and life satisfaction. Our findings revealed a substantial mediating effect, which has not been extensively examined in previous studies. These results resonate with established theories that highlight perceived social support as a primary interpersonal resource, essential for coping with stress and closely linked to psychological distress [158]. Moreover, life satisfaction has been shown to play a protective role in promoting health, reducing the risk of chronic illness, and encouraging a more active lifestyle by motivating individuals toward self-care [196].

In our mediation analysis, we identified an indirect effect of SOC on PTSD through perceived social support (SOC > PSS > PTSD) with a coefficient of 0.011, highlighting the mediating role of social support in this relationship. Similarly, the indirect effect of SOC on PTSD through life satisfaction (SOC > Life Satisfaction > PTSD) was -0.001, indicating the extent of the mediated relationship through life satisfaction. These findings provide insight into the magnitude and direction of the relationships between SOC, perceived social support, and life satisfaction.

An unexpected finding in our study was that higher perceived social support was linked to an increased likelihood of experiencing PTSD. This counterintuitive result aligns with Social Deterioration Theory, which posits that under conditions of chronic economic hardship and social instability, support networks can become sources of stress rather than relief [8,75]. In such environments, social interactions may reinforce distress due to shared trauma, financial dependence, or the emotional burden of caring for others in similar situations.

Social Deterioration Theory posits that when communities face widespread poverty, high dependency ratios, and resource scarcity, social ties rather than providing emotional or financial stability—can exacerbate stress and psychological distress. In Ekiti State, where extended family networks often struggle with extreme financial burdens, seeking social support may inadvertently increase feelings of guilt, obligation, or disappointment when expected support is unavailable or insufficient. Older adults who rely on family members may experience tension or even social withdrawal if they feel like a burden. Instead of alleviating PTSD symptoms, these strained relationships may amplify distress, underscoring the need to differentiate between functional and dysfunctional social support systems in mental health interventions for older adults in economically strained settings. In such environments, perceived social support may entail burdensome reciprocal obligations, unmet expectations, and heightened exposure to shared trauma within strained relationships, exacerbating psychological distress and increasing vulnerability to PTSD.

Contextually, Ekiti State has been characterized by declining levels of life satisfaction [136], with 73.8% of senior citizens reporting dissatisfaction with life and 81% depending on their relationships for support [10]. However, our findings reveal a notable negative indirect effect, demonstrating that senior citizens who effectively utilize SOC strategies to optimize and allocate their resources toward their chosen goals and activities are more likely to experience increased life satisfaction. This higher life satisfaction, in turn, is associated with a reduced likelihood of experiencing PTSD, suggesting a potential protective effect of life satisfaction against PTSD symptoms.

Our findings accentuates the importance of addressing both the emotional and resource-based challenges faced by the elderly, offering a path toward enhancing their quality of life and reducing the incidence of PTSD. By implementing targeted interventions that strengthen social support networks and empower senior citizens to manage their resources effectively through SOC strategies, we can better meet the mental health needs of this vulnerable population. This study contributes meaningfully to the broader field of gerontology, highlighting the role of SOC in mental health resilience among older adults in resource-limited set

Expanding on our path analysis, Hypothesis 8 examines variations in life satisfaction (LS), perceived social support (PSS), PTSD, and Selection, Optimization, and Compensation (SOC) strategies across three age groups: 65–74, 75–84, and 85 + . Our descriptive data indicate that the 65–74 group reported the highest LS (mean = 35.99) and PSS (mean = 52.08), while the 75–84 group showed a decline in LS (35.53) and PSS (49.12) along with the highest PTSD symptoms (mean = 60.08). The 85 + group further declines in PSS (46.12) and LS (34.94), though their PTSD scores (57.50) are lower than the 75–84 group.

The correlation analysis reveals that for individuals aged 75–84, LS and PSS are moderately and significantly positively correlated (r = 0.241, p = 0.037), indicating that higher social support is associated with increased life satisfaction. In contrast, the 65–74 and 85 + groups exhibit weak, non-significant correlations between LS and PSS. The moderate positive relationship in the 75–84 group aligns with prior research [248–250], whereas the slight negative correlation in the 85 + group may be influenced by contextual factors in Ekiti State—such as large family sizes, poverty, and caregiver unemployment—that undermine the benefits of social support.

In the 65–74 age group, a positive correlation (r = 0.795) between LS and PTSD, although not statistically significant, suggests that individuals in early retirement may buffer PTSD symptoms through robust social support (mean PSS = 52.08 and mean PTSD = 55.73). Conversely, the 75–84 group, which has the highest PTSD scores (60.08), shows a weak negative correlation between LS and PTSD (r = -0.117), implying that reduced life satisfaction in this group is linked to heightened distress. For the 85 + group, the positive correlation (r = 0.113) is also non-significant, highlighting the complex interplay between subjective well-being and trauma responses as individuals age.

Regarding adaptive coping, the data reveal a weak negative correlation between SOC and LS in the 65–74 (-0.076, p = 0.295) and 75–84 (-0.083, p = 0.477) groups, while a weak positive correlation (0.051, p = 0.706) is observed in the 85 + group. Additionally, SOC and PSS are weakly positively correlated in the 65–74 group (0.104, p = 0.015), negatively correlated in the 75–84 group (-0.404, p = 0.00), and strongly positively correlated in the 85 + group (0.539, p = 0.00).

These patterns can be understood through the lens of Conservation of Resources (COR) Theory [163], Socioemotional Selectivity Theory (SST) [181], and the Strength and Vulnerability Integration (SAVI) Model [251]. COR Theory posits that individuals strive to acquire, retain, and protect valuable resources—including social and psychological assets—to cope with stress. This helps explain why older adults with strong social support (in the 85 + group) may be more effective in utilizing SOC strategies, as they have a more stable resource base.

Similarly, SST suggests that as individuals age, they prioritize emotionally meaningful relationships and goals. The weak negative correlation between SOC and LS in the 65–74 and 75–84 groups could indicate that these individuals are still adjusting their strategies, balancing long-term goals with present needs. However, the strong positive relationship between SOC and PSS in the 85 + group aligns with SST’s idea that older adults prioritize social relationships to maintain well-being.

Lastly, SAVI theory emphasizes that while older adults become more skilled in emotional regulation, they are also more vulnerable to stressors when overwhelmed. The negative correlation between SOC and PSS in the 75–84 group may suggest that when external support is available, individuals in this age range may rely less on SOC strategies, potentially due to physical or emotional fatigue. Together, these findings highlight how age-related shifts in resource management and social priorities influence the effectiveness of SOC strategies in maintaining well-being.

Overall, the data indicate that vulnerability and adaptive functioning vary by age. The 65–74 group appears to be the most satisfied and least distressed, likely due to active employment or recent retirement and robust social support. In contrast, the 75–84 group, with its highest PTSD scores and declines in LS and PSS, may be particularly vulnerable—possibly exacerbated by sedentarism and transitional life challenges. The 85 + group, while exhibiting lower LS and PSS, shows a strong linkage between social support and SOC, suggesting a different adaptive profile. These findings underscore the need for tailored interventions that enhance social support and promote adaptive coping strategies across different stages of later adulthood in Ekiti State.

### Impact of sample characteristics on findings

The demographic composition of the sample likely influenced the study’s results, as participants were predominantly older adults from Ekiti State, Nigeria, a region with distinct socio-cultural and economic factors shaping their experiences. Below, we discuss key sample characteristics and their potential impact on the findings.

### Gender imbalance

The sample was predominantly male (81%**),** which may have influenced PTSD-related findings. While research on gender differences in PTSD remains mixed [252,253], some studies suggest that men and women experience different PTSD symptom clusters. In older populations, men have been found to report higher levels of hyperarousal and emotional numbing compared to women [254], which may be relevant to this study’s findings.

However, the underrepresentation of women (**19%**) limits the ability to fully examine gender-related PTSD differences. Cultural and societal factors may also play a role in this disparity, as elderly women in traditional settings are often primary caregivers and may be less likely to participate in research studies. Future research should aim for a more balanced gender distribution to improve generalizability.

### Educational attainment and economic realities

Ekiti State is often referred to as the “Fountain of Knowledge” due to its high concentration of professors and a strong emphasis on education, which is reflected in the sample profile of this study. Nearly half of the participants (48.9%) had attained education beyond secondary school, with many holding first degrees and higher qualifications. Research suggests that higher educational attainment is associated with better cognitive flexibility and problem-solving skills, which are beneficial for employing adaptive coping strategies like Selection, Optimization, and Compensation (SOC). However, despite the high levels of education within the sample, the results show a high prevalence of PTSD. This could be attributed to the socioeconomic challenges in Ekiti State, including underemployment and financial insecurity, which may limit the effectiveness of cognitive coping strategies. Moreover, limited access to mental health resources and support networks may exacerbate PTSD symptoms, even among highly educated individuals. Thus, while higher education may enhance cognitive abilities, economic realities **and** lack of resources may significantly influence the severity of PTSD in this context.

### Socio-economic challenges and employment

The employment history and socio-economic status of participants in this study likely played a significant role in shaping their mental health outcomes, especially regarding PTSD. Given that 42.27% of participants reported working in government roles (federal, state, or local), this indicates a reliance on government pensions. However, Ekiti State is currently facing a pension crisis, with an estimated 54 billion Naira in unpaid retirement benefits [54]. The financial insecurity that arises from this pension crisis may have directly contributed to heightened stress levels, potentially exacerbating PTSD symptoms.

The occupation and economic status of participants further contribute to this issue. While those employed in teaching (18.7%), trading (15.0%), and farming (15.6%) may have relatively stable occupations, these roles are often characterized by low income and limited financial security. The economic strain that comes with these occupations likely compounds feelings of financial uncertainty and stress, both of which are known risk factors for mental health issues like PTSD.

Moreover, 24.6% of participants reported being unemployed or underemployed, which is consistent with national trends and has been linked to feelings of isolation, financial stress, and a diminished sense of social support. These factors are critical in understanding why the PTSD symptoms observed in this study may have been more severe compared to what might be expected in populations with more financial stability and security.

Thus, the socio-economic context of Ekiti State, including economic insecurity, pension crises, and unemployment, likely shaped the mental health outcomes in this sample. These demographic characteristics are essential to interpreting the findings, as they underscore the role of financial and employment status in influencing mental health and PTSD symptoms in older adults. This points to the need for targeted mental health interventions that also address these broader socio-economic factors, particularly in regions with high levels of economic stress like Ekiti State.

### Marital status, family structure and housing

Marital status, family structure, and housing arrangements significantly influenced mental health outcomes in this study, as socio-economic challenges in Ekiti State likely contributed to diminished life satisfaction, which, in turn, may have exacerbated PTSD symptoms. With 72.3% of participants being married and a majority (62%) reporting four or more children, the pressures associated with raising large families in a financially constrained environment can contribute to stress, negatively impacting life satisfaction. Larger families, while providing emotional support, often come with increased financial strain, especially when pension crises and underemployment affect many in the region.

In addition, the housing situation of the participants, with 95% owning homes, points to a preference for stability, yet this may limit financial flexibility in times of economic difficulty. Unlike rented housing, homeownership doesn’t offer an easy option to adjust costs, which could lead to a greater sense of financial insecurity. Such factors contribute to diminished life satisfaction, as participants may experience chronic stress due to financial constraints and limited economic mobility.

Diminished life satisfaction can be both a direct result of economic hardship and a precursor to mental health difficulties like PTSD. When participants face unmet financial needs or struggle with large family responsibilities in a context of limited resources, their overall sense of life satisfaction can decline. This, in turn, amplifies the emotional toll of trauma exposure, potentially making them more vulnerable to severe PTSD symptoms. Therefore, the socio-economic factors tied to marital status, family structure, and housing arrangements likely play a critical role in explaining why participants in this study exhibited more severe PTSD symptoms, particularly in the context of low life satisfaction.

### Health status and age distribution

The health status of participants reveals that only 30.8% reported good health, suggesting that financial insecurity and limited healthcare access may have exacerbated PTSD symptoms. Moreover, the age distribution (59.2% in the 65–74 age group, 23.4% in 75–84, and 17.4% in 85+) reflects the well-known challenge in research where the oldest-old demographic is often underrepresented and difficult to engage in data collection. This limited number in the oldest-old group is unlikely to have significantly impacted the sample profile or the findings.

Socio-economic factors such as financial insecurity, poor health, and limited access to services likely contributed to heightened PTSD symptoms. These factors, along with the sample’s demographic characteristics, accentuate the need for addressing these issues in future studies. Future research should aim for a more balanced and diverse sample to fully capture the experiences of different subgroups.

### Implications of the study

This study has practical, theoretical, and policy implications. It underscores the protective role of Selection, Optimization, and Compensation (SOC) strategies in mitigating PTSD symptoms among senior citizens in Ekiti State. The significant negative correlation between SOC strategies and PTSD symptoms highlights the importance of incorporating these strategies into psychotherapeutic interventions and mental health programs to enhance resilience and psychological well-being in older adults.

### Practical implications

Age-specific interventions are crucial for maximizing the effectiveness of mental health programs. For the young-old (65–74), stress management techniques, mindfulness training, and active community involvement should be prioritized. The old-old (75–84) would benefit from reminiscence therapy, which fosters emotional resilience through reflections on life experiences. The oldest-old (85+) require adaptive strategies to cope with physical limitations, emphasizing holistic approaches that integrate physical, emotional, and social well-being. Connecting seniors to community resources, such as home healthcare and senior centers, is essential for enhancing their quality of life.

### Theoretical implications

This study contributes to existing resilience and aging theories by demonstrating that SOC strategies significantly reduce PTSD symptoms in older adults. The findings align with Conservation of Resources (COR) Theory, which posits that individuals strive to protect and enhance their resources; in this case, SOC strategies serve as key psychological resources against PTSD. Additionally, the study supports Social Erosion Theory, which suggests that PTSD can lead to declining social connections; however, SOC strategies help mitigate this effect by promoting engagement and adaptability. Strength and Vulnerability Integration (SAVI) Theory also applies, as it explains how older adults regulate emotions differently based on life experiences, further reinforcing the importance of targeted interventions for different age groups.

### Policy implications

Given the demonstrated mental health benefits of SOC strategies, policymakers should prioritize increased funding for senior mental health services, particularly in underserved regions like Ekiti State. Strengthening perceived social support (PSS) and promoting adaptive coping mechanisms through targeted community programs can significantly improve well-being. For younger seniors, policies should foster social connections to reduce isolation, while for the oldest-old, a shift toward personalized, holistic care addressing physical, emotional, and social dependencies is essential. Integrating SOC strategies into healthcare services and linking seniors to community resources can create a more supportive aging environment.

Indeed, this study highlights the importance of age-sensitive mental health strategies and emphasizes the theoretical foundations that explain why SOC strategies are effective in reducing PTSD symptoms. By addressing social and economic factors affecting older adults’ well-being, policymakers and healthcare providers can implement interventions that improve resilience, enhance psychological outcomes, and promote overall quality of life.

## Strength and limitations

This study makes a significant contribution to understanding the relationship between SOC strategies, PTSD, PSS, and life satisfaction among older adults in Ekiti State. Notably, it is the first study to directly link SOC strategies with PTSD and life satisfaction, making a pioneering contribution to the field. Additionally, it is the first study to explore the application of SOC strategies in Nigeria, specifically among older adults across three distinct age groups (young-old, 65–74; old-old, 75–84; and oldest-old, 85+). By examining these constructs in a resource-constrained environment, the study fills a gap in the literature and provides new insights into mental health outcomes in this under-researched population.

However, there are several limitations to the study. First, the use of composite scores for SOC, PTSD, PSS, and life satisfaction may not fully capture the domain-specific nuances of these variables. Future research could benefit from examining the subcomponents of these constructs to better understand their specific relationships and roles. Second, the absence of sociodemographic variables such as age, sex, socioeconomic status, and education limits the scope of our analysis. Lastly, while a majority of participants in Ekiti State have high educational attainment, a potential limitation is the literacy level of some participants, as a small portion had limited formal education. Although the questionnaires were originally designed in English and administered with verbal assistance in Yoruba, some participants may have faced challenges in fully understanding the questions, which could have influenced their responses. Future studies may explore additional strategies to ensure clarity and comprehension across all literacy levels. Addressing these limitations could provide a more nuanced understanding of how contextual factors influence the outcomes studied and help refine interventions for older adults.

Additionally, data collection from elderly participants posed a challenge, as it was not easy to reach them and obtain responses to the questionnaire. Future studies should consider alternative data collection methods, such as assisted interviews or community engagement strategies, to improve participation and data quality.

The study’s quantitative nature may have also overlooked valuable insights that could be captured through qualitative methods. Future research should explore older adults’ lived experiences using case studies, interviews, or focus groups to complement the quantitative findings and provide a richer, more comprehensive understanding. Finally, the study was conducted in Ekiti State, Nigeria, a region characterized by unique socioeconomic conditions, including poverty and high underemployment among senior citizens. These contextual factors limit the generalizability of the findings to other regions or populations. Future research could explore these relationships in different settings or countries to assess the applicability of the findings in diverse socio-cultural contexts. Comparative studies across regions with varying socioeconomic conditions would help to refine our understanding of how such factors influence the well-being of older adults

## Conclusion

This study aimed to explore the mediating roles of life satisfaction (LS) and perceived social support (PSS) in the relationship between selection optimization compensation (SOC) strategies and posttraumatic stress disorder (PTSD) among older adults in Ekiti State. It also examined the moderating effects of age groups (young-old, old-old, oldest-old) on the relationship between SOC and PTSD, as well as the impact of age on life satisfaction, perceived social support, PTSD, and SOC

The results show that age groups significantly moderated the relationship between SOC and PTSD, with older individuals exhibiting a stronger connection between SOC strategies and reduced PTSD symptoms. Furthermore, our mediation analysis revealed that both PSS and life satisfaction play partial mediating roles in the relationship between SOC and PTSD, suggesting that SOC strategies reduce PTSD not only directly but also through their positive influence on social support and life satisfaction. Additionally, the findings indicate that both life satisfaction and perceived social support decline with increasing age, with the oldest group reporting the lowest levels in these aspects.

This study contributes to the literature by being the first to establish a direct association between SOC strategies, PTSD, and perceived social support in older adults in Nigeria. It also represents pioneering research on PTSD among senior citizens in Nigeria, offering valuable insights into gerontopsychology within the Nigerian context. To date, no study has explored the application of SOC strategies in Nigeria or their connection to mental health outcomes like PTSD in older adults. This work lays the foundation for future research on SOC and PTSD in aging populations, particularly within resource-constrained environments.

However, this study has some limitations. It utilized a composite measure of SOC, perceived social support, life satisfaction, and PTSD, which may not fully capture the nuances of each component and their specific effects on PTSD. Nevertheless, this approach provided a comprehensive initial exploration of the relationship between SOC and PTSD in this under-researched population. Additionally, the cross-sectional design limits causal inferences. The data collection process relied on self-reported measures, making it susceptible to recall bias and social desirability effects. Moreover, collecting data from older adults presented challenges, including cognitive fatigue, varying literacy levels, and difficulties in recalling past experiences, which may have influenced the accuracy of responses.

Despite these limitations, this exploratory study offers valuable insights that can inform future longitudinal research and intervention strategies. Future studies should examine the long-term impact of SOC strategies and the role of social support in mitigating PTSD, particularly in resource-limited settings like Ekiti State. Strengthening SOC strategies and enhancing social support networks through targeted interventions may further improve the mental health and well-being of older adults in similar contexts.

## Supporting information

S1 File Additional references. (DOCX)
