# Supplementary material for: Examining the indirect effects of life satisfaction and perceived social support on selection optimization compensation and PTSD among the senior citizens of Ekiti state: A moderated mediation approach
Source: PLOS Ment Health. 2025 Jun 24;2(6):e0000186. doi: 10.1371/journal.pmen.0000186 (PMC12798381; doi:10.1371/journal.pmen.0000186)
Supplement: S1 File — (DOCX) [file pmen.0000186.s001.docx]

Aarts, P., & Op den Velde, W. (1996). Prior traumatization and the process of aging. In B. Van der Kolk, A. McFarlane, & L. Weisaeth (Eds.), Traumatic stress: The effects of overwhelming experience on mind, body, and society (pp. 359–377). Guilford Press.

Abrahams, N., Devries, K., Watts, C., Pallitto, C., Petzold, M., Shamu, S., et al. (2014). Worldwide prevalence of non-partner sexual violence: A systematic review. The Lancet, 383(9929), 1648–1654. https://doi.org/10.1016/S0140-6736(13)62243-6

Adebayo, S. O., & Arogundade, O. B. (2011). Determinants of Significant Single Best Predictor of Life Satisfaction among Nigerian Adults. Interdisciplinary Review of Economics and Management, 1(1).

Adepoju, A. (2020). Aging and social security challenges in Nigeria: Policy implications and future directions. African Journal of Social Sciences, 15(2), 45-67.

Adler, A. B., & Castro, C. A. (2013). An occupational mental health model for the military. Military Behavioral Health, 1(1), 41–45. https://doi.org/10.1080/21635781.2013.795763

Aina, F.O., Fakuade, B.O., Agbesanwa, T.A., Dada, M.U. and Fadare, J.O. (2023) Association between Support and Satisfaction with Life among Older Adults in Ekiti, Nigeria: Findings and Implications. Open Journal of Medical Psychology, 12, 117-128. https://doi.org/10.4236/ojmp.2023.123007

Alamdari, G., & Laganà, L. (2015). The relationship of older adults' physical pain to depression and post-traumatic stress disorder (PTSD): A review. Journal of Geriatric Medicine and Gerontology, 1(2), 007

Allemand, M., Schafhuser, K., & Martin, M. (2015). Long-term correlated change between personality traits and perceived social support in middle adulthood. Personality and Social Psychology Bulletin, 41(3), 420–432. https://doi.org/10.1177/0146167214568472

Allerhand, M., Gale, C. R., & Deary, I. J. (2014). The dynamic relationship between cognitive function and positive well-being in older people: A prospective study using the English Longitudinal Study of Aging. Psychology and Aging, 29(2), 306-318. https://doi.org/10.1037/a0036551

Andrykowski, M. A., & Cordova, M. J. (1998). Factors associated with PTSD symptoms following treatment for breast cancer: Test of the Andersen model. Journal of Traumatic Stress, 11(2), 189-203. https://doi.org/10.1023/a:1024490718043

Andualem F, Melkam M, Takelle GM, Nakie G, Tinsae T, Fentahun S, Rtbey G, Begashaw TD, Seid J, Tegegn LF, Gedef GM, Bitew DA and Godana TN (2024) Prevalence of posttraumatic stress disorder and associated factors among displaced people in Africa: a systematic review and meta-analysis. Front. Psychiatry 15:1336665. doi: 10.3389/fpsyt.2024.1336665

Mission reference these were previous correction made when the reviewers said that there were missing references. I later found the reference and I acknowledged it here as you are seeing in various sub-portion of the reference list below:

**Antonucci, T. C., Fiori, K. L., Birditt, K. S., & Jackey, L. M. H. (2014).** Convoys of social relations: Integrating life-span and life-course perspectives. Life-span Developmental Psychology, 2(1), 1-34.

**Upgraded reference**

American Psychiatric Association. (2022). Diagnostic and statistical manual of mental disorders (5th ed., text rev.). American Psychiatric Publishing

Mission reference

Aspinwall, L.G., Taylor, S.E., 1997. A stitch in time: self-regulation and proactive coping. Psychol. Bull. 121, 417–436

Asuquo, J. E., Edet, B. E., Abang, I. E., Essien, E. A., Osakwe, O. G., Aigbomain, E. J., & Chigbundu, K. C. (2017). Depression and posttraumatic stress disorder among road traffic accident victims managed in a tertiary hospital in Southern Nigeria. Nigerian Journal of Clinical Practice, 20(2), 170–175. https://doi.org/10.4103/1119-3077.196114

Arogundade, O. B., & Adebayo, S. O. (2011). The role of age in life satisfaction judgment among educated adults in Ado–Ekiti. *African Research Review: An International*

*Multidisciplinary Journal, Ethiopia*, *5*(6), 304-313. https://doi.org/10.4314/afrrev.v5i6.66160

Aspinwall, L. G., & Taylor, S. E. (1997). A stitch in time: Self-regulation and proactive coping. *Psychological Bulletin*, *121*(3), 417–436. https://doi.org/10.1037/0033-2909.121.3.417

Atwoli, L., Stein, D. J., Williams, D. R., Mclaughlin, K. A., Petukhova, M., Kessler, R. C., & Koenen, K. C. (2013). Trauma and posttraumatic stress disorder in South Africa: Analysis from the South African Stress and Health Study. BMC Psychiatry, 13, 182. https://doi.org/10.1186/1471-244X-13-182

Au, T. M., Dickstein, B. D., Comer, J. S., Salters-Pedneault, K., & Litz, B. T. (2013). Co-occurring posttraumatic stress and depression symptoms after sexual assault: A latent profile analysis. *Journal of Affective Disorders*, *149*, 209-216. https://doi.org/10.1016/j.jad.2013.01.034

Avrill PM, & Beck JG (2000). Posttraumatic stress disorder in older adults: A conceptual review. Journal of Anxiety Disorders, 14, 133–156. [PubMed: 10864382]

Missing reference

Baars, J. (2007). Chronological time and chronological age: Problems of temporal diversity. In J. Baars & H. Visser (Eds.), Aging and time: Multidisciplinary perspectives (pp. 1–13). Amityville, NY: Baywood Publishing.

Baker, C., & Kirk-Wade, E. (2024, March 1). Mental health statistics: Prevalence, services and funding in England. NHS Digital.

Bachmann, C. J., Czwikla, J., Jacobs, H., Fegert, J. M., & Hoffmann, F. (2017). Prevalence and treatment of posttraumatic stress disorder in Germany: An analysis of nationwide health insurance data. *European Journal of Psychotraumatology*, *8*(1), 1347565. https://doi.org/10.1080/20008198.2017.1347565

Bah, A. J., James, P. B., Bah, N., Sesay, A. B., Sevalie, S., & Kanu, J. S. (2020). Prevalence of anxiety, depression, and post-traumatic stress disorder among Ebola survivors in northern Sierra Leone: A cross-sectional study. BMC Public Health, 20, Article 1391. https://doi.org/10.1186/s12889-020-09488-8

Found missing reference

**Baker, C., & Kirk-Wade, E. (2024).** Mental health statistics: Prevalence, services, and funding in England. House of Commons Library. https://commonslibrary.parliament.uk

Removed repeated reference

Baltes, P. B., & Baltes, M. M. (1990). Psychological perspectives on successful aging: The model of selective optimization with compensation. In P. B. Baltes & M. M. Baltes (Eds.), *Successful aging* (pp. 1–34). Cambridge University Press.

Baltes, P. B. (1987). Theoretical propositions of life span developmental psychology: On the dynamics between growth and decline. Developmental Psychology, 23, 611–626

Baltes, P. B. (1997). On the incomplete architecture of human ontogeny. *American Psychologist*, *52*(4), 366–380. https://doi.org/10.1037/0003-066X.52.4.366

Baltes, M. M., & Carstensen, L. L. (2003). The process of successful aging: Selection, optimization, and compensation. In U. M. Staudinger & U. Lindenberger (Eds.), *Understanding human development: Dialogs with lifespan psychology* (pp. 81-104). Kluwer Academic Publishers.

**Baltes, P. B., & Lang, F. R. (1997).** Everyday functioning and successful aging: The impact of resources. *Psychology and Aging*, 12(3), 433–443. https://doi.org/10.1037/0882-7974.12.3.433

Bar-Haim, Y., Stein, M. B., Bryant, R. A., Bliese, P. D., Ben Yehuda, A., Kringelbach, M. L., Jain, S., & Pine, D. S. (2021). Intrusive traumatic reexperiencing: Pathognomonic of the psychological response to traumatic stress. American Journal of Psychiatry, 178(2), 119-126. https://doi.org/10.1176/appi.ajp.2020.19121231

Basharpoor, S., & Eyni, S. (2021). Developing a causal model of life satisfaction of veterans with PTSD based on loneliness: The moderating role of perceived social support. *Journal of Military Caring Sciences*, *8*(4), 206-217. https://doi.org/10.22037/jmcs.v8i4.40605

Birkeland, M. S., Knatten, C. K., Hansen, M. B., Hem, C., & Heir, T. (2016). Long-term relationships between perceived social support and posttraumatic stress after the 2011 Oslo bombing: A three-year longitudinal study. *Journal of Affective Disorders*, *202*, 230–235. https://doi.org/10.1016/j.jad.2016.03.037

Bishop, A., Younan, R., Low, J., & Pilkington, P. D. (2022). Early maladaptive schemas and depression in adulthood: A systematic review and meta-analysis. *Clinical Psychology & Psychotherapy*, *29*(1), 111–130. https://doi.org/10.1002/cpp.2587

Blanchard-Fields, F., Chen, Y., & Norris, L. (1997). Everyday problem solving across the adult life span: The influence of domain specificity and cognitive appraisal. *Psychology and Aging*, *12*, 684–693. https://doi.org/10.1037/0882-7974.12.4.684

Blanchard-Fields, F., Jahnke, H. C., & Camp, C. (1995). Age differences in problem-solving style: The role of emotional salience. *Psychology and Aging*, *10*, 173–180. https://doi.org/10.1037/0882-7974.10.2.173

Bola, B. (n.d.). Education as a correlate of life satisfaction among retired older people in Lagos State, Nigeria. University of Lagos, Faculty of Social Sciences.

Borg, C., Hallberg, I. R., & Blomqvist, K. (2006). Life satisfaction among older people (65+) with reduced self-care capacity: The relationship to social, health, and financial aspects. *Journal of Clinical Nursing*, *15*(5), 607–618. https://doi.org/10.1111/j.1365-2702.2006.01339.x

Missing reference

Brady, K. T., Killeen, T. K., Saladin, M. E., Dansky, B. S., & Becker, S. (2000). Comorbid substance abuse and posttraumatic stress disorder: Characteristics, treatment strategies, and 12-step fellowship involvement. Journal of Substance Abuse Treatment, 19(1), 1-8.

Brandstadter, J., & Greve, W. (1994). The aging self: Stabilizing and protective processes. *Developmental Review, 14*(1), 52–67. https://doi.org/10.1006/drev.1994.1003

Brandtstädter, J. (1999). The self in action and development: Cultural, biological, and ontogenetical bases of intentional self-development. In J. Brandtstädter & R. M. Lerner (Eds.), *Action and self-development: Theory and research through the life span* (pp. 37–65). Thousand Oaks, CA: Sage.

Brandstädter, J., & Rothermund, K. (2002). The life-course dynamic of goal pursuit and goal adjustment: A two-process framework. *Developmental Review, 22*(1), 1–24.

**Breslau, N., Davis, G. C., Peterson, E. L., & Schultz, L. R. (2000).** A second look at comorbidity in victims of trauma: The posttraumatic stress disorder–major depression connection. Biological Psychiatry, 48(9), 902-909.
[DOI: 10.1016/S0006-3223(00)00933-1]

**Missing refrence**

Breslau, N., Chilcoat, H. D., Kessler, R. C., & Davis, G. C. (1999). Previous exposure to trauma and PTSD effects of subsequent trauma: Results from the Detroit area survey of trauma. *American Journal of Psychiatry, 156*(6), 902–907.

Brewin, C. R., Andrews, B., & Valentine, J. D. (2000). Meta-analysis of risk factors for posttraumatic stress disorder in trauma-exposed adults. *Journal of Consulting and Clinical Psychology, 68*(5), 748–766.

British Broadcasting Corporation (BBC). (2020, October 3). Nduka Orjinmo and Salihu Adamu. Why some Nigerian families lock up children and the mentally ill. Retrieved October 8, 2020, from BBC News

**Brodaty, H., Joffe, C., Luscombe, G., & Thompson, C. (2004).** Vulnerability to post-traumatic stress disorder and psychological morbidity in aged Holocaust survivors. International Journal of Geriatric Psychiatry, 19(10), 968-979. https://doi.org/10.1002/gps.1178

Carstensen, L. L. (1995). Evidence for a life-span theory of socioemotional selectivity. *Current Directions in Psychological Science, 4*(5), 151–156. https://doi.org/10.1111/1467-8721.ep11512261

Carstensen, L. L., Isaacowitz, D. M., & Charles, S. T. (1999). Taking time seriously: A theory of socioemotional selectivity. *American Psychologist, 54*(3), 165–181. https://doi.org/10.1037/0003-066X.54.3.165

Carver, C. S., Scheier, M. F., & Segerstrom, S. C. (2010). Optimism. *Clinical Psychology Review, 30*(7), 879–889. https://doi.org/10.1016/j.cpr.2010.01.006

Charles, S. T. (2010). Strength and vulnerability integration: A model of emotional well-being across adulthood. *Psychological Bulletin, 136*(6), 1068–1091. https://doi.org/10.1037/a0021232

Charles, S. T., & Carstensen, L. L. (2009). Social and emotional aging. *Annual Review of Psychology, 61*, 383–409. https://doi.org/10.1146/annurev.psych.093008.100448

Missing reference

Chima, M. K., Halvorsen, C. J., & Okoye, U. O. (2022). Aging in Nigeria: A growing population of older adults requires the implementation of national aging policies.

Chopra MP, Zhang H, Pless Kaiser A, Moye JA, Llorente MD, Oslin DW, & Spiro A 3rd. (2016). PTSD is a chronic, fluctuating disorder affecting the mental quality of life in older adults. The American Journal of Geriatric Psychiatry : Official Journal of the American Association for Geriatric Psychiatry, 22(1), 86–97. doi:10.1016/j.jagp.2013.01.064

**Christiansen, D. M., & Elklit, A. (2008).** Risk factors predict post-traumatic stress disorder differently in men and women. Annals of General Psychiatry, 7(1), 24. https://doi.org/10.1186/1744-859X-7-24

Chruœciel, P., Kulik, T., Jakubowska, K., & Nalepa, D. (2018). Differences in the perception of social support among rural area seniors: A cross-sectional survey of Polish population. *International Journal of Environmental Research and Public Health, 15*(6), 1288. https://doi.org/10.3390/ijerph15061288

Missing references

Clapp, J. D., & Beck, J. G. (2008). Understanding the relationship between PTSD and social support: The role of negative network orientation. Behaviour Research and Therapy, 47(3), 237–244. https://doi.org/10.1016/j.brat.2008.12.006

**CNBC Africa.** (2020, July 29). Mental health legislation could help Nigeria survive COVID-19

Cobb, S. (1976). Social support as a moderator of life stress. *Psychosomatic Medicine, 38*(5), 300–314.

Cohen, S., & Wills, T. A. (1985). Stress, social support, and the buffering hypothesis. *Psychological Bulletin, 98*(2), 310–357.

Cohen, S. (2004). Social relationships and health. *American Psychologist, 59*(8), 676–684. https://doi.org/10.1037/0003-066X.59.8.676

Cook, J. M., McCarthy, E., & Thorp, S. R. (2017). Older adults with PTSD: Brief state of research and evidence-based psychotherapy case illustration. American Journal of Geriatric Psychiatry, 25(2), 107-115. https://doi.org/10.1016/j.jagp.2016.12.016

Cook, J. M., & Niederehe, G. (2007). Trauma in older adults. In M. J. Friedman, T. M. Keane, & P. A. Resick (Eds.), *Handbook of PTSD: Science and practice* (pp. 24-37). Guilford Press.

Couette, M., Mouchabac, S., Bourla, A., Nuss, P., & Ferreri, F. (2020). Social cognition in posttraumatic stress disorder: A systematic review. *British Journal of Clinical Psychology, 59*(2), 117–138. https://doi.org/10.1111/bjc.12186

Cruz, N., & Ribeiro, P. (2024). Relation between the SOC model and depressive symptoms in the elderly. Revista Interamericana de Psicología/Interamerican Journal of Psychology, 58(1), e1746.

Darves-Bornoz, J. M., Alonso, J., de Girolamo, G., de Graaf, R., Haro, J. M., Kovess-Masfety, V., & Gasquet, I. (2008). Main traumatic events in Europe: PTSD in the European study of the epidemiology of mental disorders survey. *Journal of Traumatic Stress, 21*(5), 455–462. https://doi.org/10.1002/jts.20357

Deeg, D. J. H., van Zonneveld, R. J., van der Maas, P. J., & Habbema, J. D. F. (1989). Medical and social predictors of longevity in older individuals: Total predictive value and interdependence. *Social Science & Medicine, 29*(11), 1271–1280.

Desmarais, P., Weidman, D., Wesse, A., Bruneau, M. A., & Friedland, (2020). The interplay between posttraumatic stress disorder and dementia: A systematic review. *American Journal of Geriatric Psychiatry, 28*(1), 48–60. https://doi.org/10.1016/j.jagp.2019.06.004

Diener, E., & Suh, M. E. (1998). Subjective well-being and age: An international analysis. In K. W. Schaie & M. P. Lawton (Eds.), *Annual review of gerontology and geriatrics: Focus on emotions and adult development* (Vol. 17, pp. 304–324). New York, NY: Springer.

Diener, E., Emmons, R. A., Larsen, R. J., & Griffin, S. (1985). The Satisfaction With Life Scale. *Journal of Personality Assessment, 49*(1), 71–75. https://doi.org/10.1207/s15327752jpa4901_13

Diener, E., Suh, E. M., Lucas, R. E., & Smith, H. L. (1999). Subjective well-being: Three decades of progress. *Psychological Bulletin, 125*, 276–302. https://doi.org/10.1037/0033-2909.125.2.276

DiMauro, J., Renshaw, K. D., Smith, B. N., & Vogt, D. (2016). Perceived support from multiple sources: Associations with PTSD symptoms. *Journal of Traumatic Stress, 29*(4), 332–339. https://doi.org/10.1002/jts.22114

Disney, R. (2000). Crisis in public pension programmes in OECD: What are the reform options? *The Economic Journal, 110*, 1–13. https://doi.org/10.1111/1468-0297.00500

Dokpesi, A. O. (2015). The future of elderly care in Nigeria: Borrowing a leaf from a foreign land. Ageing International, 40(2), 81–97. doi:10.1007/s12126-014-9205-z

Doron-LaMarca S, Niles BL, King DW, King LA, Pless Kaiser A, & Lyons MJ (2015). Temporal associations among chronic PTSD symptoms in U.S. combat veterans. Journal of Traumatic Stress, 28(5), 410–417. doi:10.1002/jts.22039 [PubMed: 26367017]

Duncan Wallace. (2020). Posttraumatic stress disorder in Australia. *Australasian Psychiatry, 28*(3), 251–252. https://doi.org/10.1177/1039856220922245

Ebimgbo, S. O., & Okoye, U. O. (2021). Aging in Nigeria. In Aging in Nigeria. Springer. https://doi.org/10.1007/978-3-030-76501-9_2

Ehlers, A., & Clark, D. M. (2000). A cognitive model of posttraumatic stress disorder. *Behavior Research and Therapy, 38*, 319–345. https://doi.org/10.1016/S0005-7967(99)00024-5

Ekman, P. (1999). Basic emotions. In T. Dalgleish & M. J. Power (Eds.), *Handbook of cognition and emotion* (pp. 45–60). New York, NY: John Wiley & Sons.

Ellison, C. G. (1991). Religious involvement and subjective well-being. *Journal of Health and Social Behavior, 32*, 80–99. https://doi.org/10.2307/2136801

**EpiAFRIC.** (n.d.). About EpiAFRIC. Retrieved October 6, 2020, from [Insert URL if available]

Federal Ministry of Health, Nigeria. (2018). Second National Strategic Health Development Plan 2018-2022. [Accessed 6 Oct. 2020].

Feller, S., Teucher, B., Kaaks, R., Boeing, H., & Vigl, M. (2013). Life satisfaction and risk of chronic diseases in the European Prospective Investigation into Cancer and Nutrition (EPIC)-Germany study. *PLOS ONE, 8*(8), e70085. https://doi.org/10.1371/journal.pone.0070085

Missing reference

Freedman, S. A., Gilad, M., Ankri, Y., Roziner, I., & Shalev, A. Y. (2015). Social relationship satisfaction and PTSD: Which is the chicken and which is the egg? European Journal of Psychotraumatology, 6, 28864. https://doi.org/10.3402/ejpt.v6.28864

Freund, A. M., & Baltes, P. B. (2002). Life-management strategies of selection, optimization, and compensation: Measurement by self-report and construct validity. *Journal of Personality and Social Psychology, 82*(4), 642–661. https://doi.org/10.1037/0022-3514.82.4.642

Freund, A. M. (2008). Successful aging as management of resources: The role of selection, optimization, and compensation. *Research in Human Development, 5*(2), 94–106. https://doi.org/10.1080/15427600802058326

**Freund, A. M., & Baltes, P. B. (1998).** Selection, optimization, and compensation as strategies of life management: Correlations with subjective indicators of successful aging. Psychology and Aging, 13(4), 531–543. https://doi.org/10.1037/0882-7974.13.4.531

**Freund, A. M., & Baltes, P. B. (1999).** Selection, optimization, and compensation as strategies of life management: Correlations with subjective indicators of successful aging. Psychology and Aging, 14(4), 531–543. https://doi.org/10.1037/0882-7974.14.4.531

Missing reference

Freund, A. M., & Baltes, P. B. (2000). The orchestration of selection, optimization and compensation: An action–theoretical conceptualization of a theory of developmental regulation. In W. J. Perrig & A. Grob (Eds.), Control of human behavior, mental processes, and consciousness: Essays in honor of the 60th birthday of August Flammer (pp. 35–58). Lawrence Erlbaum Associates Publishers.

Freund, A. M., & Baltes, P. B. (2002a). Life-management strategies of selection, optimization and compensation: Measurement by self-report and construct validity. Journal of Personality and Social Psychology, 82, 642 662.

Freund, A. M., & Baltes, P. B. (2002b). The adaptiveness of selection, optimization, and compensation as strategies of life management: evidence from a preference study on proverbs. The Journals of Gerontology. Series B, Psychological Sciences and Social Sciences, 57, 426 434.

Galea, S., Rockers, P., Saydee, G., Macauley, R., Varpilah, S., & Kruk, M. (2010). Persistent psychopathology in the wake of civil war: Long-term posttraumatic stress disorder in Nimba county, Liberia. American Journal of Public Health, 100(9), 1745–1751.

Geng, F., Li, S., Yang, Y., Zou, J., Tu, L., & Wang, J. (2021). Trauma exposure and posttraumatic stress disorder in a large community sample of Chinese adults. Journal of Affective Disorders, 291, 368-374. https://doi.org/10.1016/j.jad.2021.05.051

Missing reference

George LK (2010). Still happy after all these years: research frontiers on subjective well-being in later life. Journals of Gerontology: Psychological Sciences and Social Sciences 65B, 331–339

Gerstorf, D., Ram, N., Röcke, C., Lindenberger, U., & Smith, J. (2008). Decline in life satisfaction in old age: Longitudinal evidence for links to distance-to-death. *Psychology and Aging, 23*(1), 154–168. https://doi.org/10.1037/0882-7974.23.1.154

Ghimire, S., Singh, D. R., Nath, D., Jeffers, E. M., & Kaphle, M. (2018). Adult children’s migration and well-being of left-behind Nepalese elderly parents. *Journal of Epidemiology and Global Health, 8*(3), 154–161. https://doi.org/10.2991/jegh.k.180511.001

Glick, D. M., Cook, J. M., Moye, J., & Kaiser, A. P. (2018). Assessment and treatment considerations for post-traumatic stress disorder at end of life. *American Journal of Hospice & Palliative Medicine, 35*(8), 11–33. https://doi.org/10.1177/1049909118764208

Reference recoved

Gorman, M. (2000). "The Growing Problem of Violence Against Older Persons in Africa" Southern African Journal of Gerontology.

Gross J.J., Carstensen L.L., Pasupathi M., Tsai J., Skorpen C.G., Hsu A.Y.C. (1997). Emotion and aging: Experience, expression, and control. Psychology and Aging, 12, 590–599

Grove, L. J., Loeb, S. J., & Penrod, J. (2009). Selective optimization with compensation: a model for elder health programming. Clinical Nurse Specialist CNS, 23(1), 25- 32. https://doi.org/10.1097/01.NUR.0000343080.57838.2f

Missing reference

Grubaugh, A. L., Elhai, J. D., Cusack, K. J., Wells, C., & Frueh, B. C. (2006). Screening for PTSD in public-sector mental health settings: The diagnostic utility of the PTSD Checklist. Depression and Anxiety, 24(2), 124–129. https://doi.org/10.1002/da.20226

Gureje, O., & Alem, A. (2002). Mental health policy development in Africa. *Bulletin of the World Health Organization, 78*(4), 475–482. https://www.ncbi.nlm.nih.gov/pmc/articles/PMC2569425/

Gureje, O., Lasebikan, V. O., Ephraim-Oluwanuga, O., Olley, B. O., & Kola, L. (2005). Community study of knowledge of and attitude to mental illness in Nigeria. *British Journal of Psychiatry, 186*, 436–441. https://doi.org/10.1192/bjp.186.5.436

Missing reference

Haber, M. G., Cohen, J. L., Lucas, T., & Baltes, B. B. (2007). The relationship between self-reported received and perceived social support: A meta-analytic review. American Journal of Community Psychology, 39(1-2), 133-144.

Hair, J. F., Black, W. C., Babin, B. J., & Anderson, R. E. (2010). Multivariate Data Analysis (7th ed.). Pearson.

Harandi, T. F., Taghinasab, M. M., & Nayeri, T. D. (2017). The correlation of social support with mental health: A meta-analysis. *Electronic Physician, 9*(9), 5212–5222. https://doi.org/10.19082/5212

Corrected references

Hartman-Stein, P. E., & Potkanowiez, E. S. (2003). Behavioral determinants of healthy aging: God news for the baby boomer generation. 2003 Online Journal of Issues in Nursing 8, 2

Haveman-Nies, A., Groot, L. L. P. G. M., & Staveren, W. A. V. (2003). Dietary quality, lifestyle factors and healthy ageing in Europe: The SENECA study Age and Ageing 32 427-434

**He, W. (2022).** Why study aging in Africa, the region with the world’s youngest population? [Website Name]. Retrieved from [URL]

Headley, B. W., Kelley, J., & Wearing, A. J. (1993). Dimensions of mental health: Life satisfaction, positive affect, anxiety, and depression. *Social Indicators Research, 29*(1), 63–82. https://doi.org/10.1007/BF01079081

Hegel, M. T., Unützer, J., Tang, L., Areán, P. A., Katon, W., Noël, P. H., Williams, J. W. Jr., & Lin, E. H. B. (2005). Impact of comorbid panic and posttraumatic stress disorder on outcomes of collaborative care for late-life depression in primary care. The American Journal of Geriatric Psychiatry, 13(1), 48–58. https://doi.org/10.1176/appi.ajgp.13.1.48

Hertz, D. (1990). Trauma and nostalgia. *International Journal of Psychiatry and Related Sciences, 27*, 189–198.

Hilft, B. E. (2017). *World risk report: Analysis and prospects*.

Hiskey, S., Luckie, M., Davies, S., & Brewin, C. R. (2008). The emergence of posttraumatic distress in later life: A review. *Journal of Geriatric Psychiatry and Neurology, 21*, 232–241. https://doi.org/10.1177/0891988708321674

MISSING REFERENCES

Hobfoll, S.E. (1988). The ecology of stress. New York: Hemisphere Publishing Corporation.

Hobfoll, S.E. (1989). Conservation of resources: A new attempt at conceptualizing stress. American Psychologist, 44, 513±524. Hobfoll, S.E. (1998). Stress, culture, and community: The psychology and philosophy of stress. New York: Plenum

Hobfoll, S. E., Canetti-Nisim, D., & Johnson, R. J. (2006). Exposure to terrorism, stress-related mental health symptoms, and defensive coping among Jews and Arabs in Israel. *Journal of Consulting and Clinical Psychology, 74*, 207–218. https://doi.org/10.1037/0022-006X.74.2.207

Hobfoll, S. E., Halbesleben, J., Neveu, J.-P., & Westman, M. (2018). Conservation of resources in the organizational context: The reality of resources and their consequences. Annual Review of Organizational Psychology and Organizational Behavior, 5(1), 103–128. https://doi.org/10.1146/annurev-orgpsych-032117-104640

Holeva, V., Tarrier, N., & Wells, A. (2001). Prevalence and predictors of acute stress disorder and PTSD following road traffic accidents: Thought control strategies and social support. *Behavior Therapy, 32*, 65–83. https://doi.org/10.1016/S0005-7894(01)80009-1

Corrected reference

Holstein, M. B., & Minkler, M. (2003). Self, society, and the "new gerontology". The Gerontologist, 43(6), 787-796.

Holt-Lunstad J, Smith TB, Layton JB (2010) Social Relationships and Mortality Risk: A Meta-analytic Review. PLoS Med 7(7): e1000316. doi:10.1371/ journal.pmed.1000316

House, J. S. (1987). Social support and social structure. *Sociological Forum, 2*, 135–146. https://doi.org/10.1007/BF01110002

**Wrongly cited**

**Igbolo, M. A., Salami, U. J., & Uzochukwu, C. O. (2017).** The impact of family and social support on the health and well-being of people in Calabar Metropolis, Cross River State, Nigeria. European Journal of Social Sciences Studies. https://doi.org/10.46827/ejsss.v0i0.54

Iteke, O., Bakare, M. O., Agomoh, A. O., Uwakwe, R., & Onwukwe, J. U. (2011). Road traffic accidents and posttraumatic stress disorder in an orthopedic setting in southeastern Nigeria: A controlled study. *Scandinavian Journal of Trauma, Resuscitation and Emergency Medicine, 19*(1), 39. https://doi.org/10.1186/1757-7241-19-39

Jenkins, R., Othieno, C., Omollo, R., Ongeri, L., Sifuna, P., Mboroki, J. K., Kiima, D., & Ogutu, B. (2015). Probable post-traumatic stress disorder in Kenya and its associated risk factors: A cross-sectional household survey. International Journal of Environmental Research and Public Health, 12(10), 13494–13509. https://doi.org/10.3390/ijerph121013494

Jimenez, D. E., Alegria, M., Chen, C., Chan, D., & Laderman, M. (2010). Prevalence of psychiatric illnesses in older ethnic minority adults. *Journal of the American Geriatrics Society, 58*, 256–264. https://doi.org/10.1111/j.1532-5415.2009.02685.x

Missing reference

Johansen, V. A., Milde, A. M., Nilsen, R. M., Breivik, K., Nordanger, D. Ø., Stormark, K. M., & Weisæth, L. (2020). The relationship between perceived social support and PTSD symptoms after exposure to physical assault: An 8-year longitudinal study. *Journal of Interpersonal Violence, 37*(9-10), NP7679–NP7706. https://doi.org/10.1177/0886260520970314

Jopp, D., & Smith, J. (2006). Resources and life-management strategies as determinants of successful aging: On the protective effect of selection, optimization, and compensation.

Jukic, M., Lukinac, A. M., Pozain, I., Talapko, J., Jukic, M., & Flakovic, P. (2020). The role of perceived social support in assessing posttraumatic stress disorder and mental health-related quality of life in veterans. Healthcare (Basel), 8(4), 396. https://doi.org/10.3390/healthcare8040396

Julia, N. (2023). Posttraumatic stress disorder (PTSD) statistics: 2023 update. Center for Advancing Health. https://cfah.org/ptsd-statistics

Kaniasty, K., & Norris, F. H. (2008). Longitudinal linkages between perceived social support and posttraumatic stress symptoms: Sequential roles of social causation and social selection. Journal of Trauma Stress, 21(3), 274–281. https://doi.org/10.1002/jts.20334

Karatzias, T., Chouliara, Z., Power, K., Brown, K., Bequm, M., & Goldrick, T. (2013). Life satisfaction in people with posttraumatic stress disorder. Journal of Mental Health, 22(6), 501-508. https://doi.org/10.3109/09638237.2013.832546

Kawakami, N., Tsuchiya, M., Umeda, M., Koenen, K. C., & Kessler, R. C., & The World Mental Health Survey Japan. (2014). Trauma and posttraumatic stress disorder in Japan: Results from the World Mental Health Japan Survey. Journal of Psychiatric Research, 53, 157-165. https://doi.org/10.1016/j.jpsychires.2014.02.003

Keane, T. M., Scott, W. O., Chavoya, G. A., Lamparski, D. M., & Fairbank, J. A. (1985). Social support in Vietnam veterans with posttraumatic stress disorder: A comparative analysis. Journal of Consulting and Clinical Psychology, 53(1), 95–102. https://doi.org/10.1037/0022-006X.53.1.95

Keltner, D., & Gross, J. J. (1999). Functional accounts of emotions. Cognition and Emotion, 13(5), 467–480. https://doi.org/10.1080/026999399379140

Kessler, R. C., Berglund, P., Demler, O., Jin, R., Merikangas, K. R., & Walters, E. E. (2005). Lifetime prevalence and age-of-onset distributions of DSM-IV disorders in the National Comorbidity Survey Replication. Archives of General Psychiatry, 62(6), 593-602. https://doi.org/10.1001/archpsyc.62.6.593

Missing reference

Kessler, R. C., Sonnega, A., Bromet, E., Hughes, M., & Nelson, C. B. (1995). Posttraumatic stress disorder in the National Comorbidity Survey. *Archives of General Psychiatry*, 52(12), 1048–1060. jamanetwork.com

Kiarsipour, N., Borhani, F., Esmaeili, R., & Zayeri, F. (2017). The correlation of aging perceptions and life satisfaction in Iranian older adults. Annals of Tropical Medicine and Public Health, 10(4), 861.

Kim, J. B., Ryu, S. Y., & Ahn, H. (2005). A review of Korean mental health studies related to trauma and disasters. Psychiatry Investigation, 2(2), 22-30.

Kline, R. B. (2015). Principles and Practice of Structural Equation Modeling (4th ed.). Guilford Press.

Kimerling, R., Allen, M. C., & Duncan, L. E. (2018). Chromosomes to social contexts: Sex and gender differences in PTSD. Current Psychiatry Reports, 20(12), 1–9. https://doi.org/10.1007/s11920-018-0981-0

King, D. W., King, L. A., Foy, D. W., Keane, T. M., & Fairbank, J. A. (1999). Posttraumatic stress disorder in a national sample of female and male Vietnam veterans: Risk factors, war-zone stressors, and resilience recovery variables. Journal of Abnormal Psychology, 108(1), 164-170. https://doi.org/10.1037/0021-843X.108.1.164

Missing reference

Koenen KC, Harney R, Lyons MJ, Wolfe J, Simpson JC, Goldberg J, Eisen SA, Tsuang M. A twin registry study of familial and individual risk factors for trauma exposure and posttraumatic stress disorder. J Nerv Ment Dis 2002;190:209–218. [PubMed: 11960081]

Kogan J, Edelstein B, & McKee D (2000). Assessment of anxiety in older adults: Current status. Journal of Anxiety Disorders, 14(2), 109–132. [PubMed: 10864381]

Krause, N. (1999). Assessing change in social support during late life. Research on Aging, 21(4), 539–569. https://doi.org/10.1177/0164027599214001

Kunzmann, U., Little, T. D., & Smith, J. (2000). Is age-related stability of subjective well-being a paradox? Cross-sectional and longitudinal evidence from the Berlin Aging Study. Psychology and Aging, 15(3), 511–526. https://doi.org/10.1037/0882-7974.15.3.511

Kydd, A., Fleming, A., Paoletti, I., & Hvalic-Touzery, S. (2020). Exploring terms used for the oldest old in the gerontological literature. Journal of Aging and Social Change, 10(2).

Missing reference

**Lampinen, P., Heikkinen, R.-L., Kauppinen, M., & Heikkinen, E. (2006).** Activity as a predictor of mental well-being among older adults. Aging & Mental Health, 10(5), 454–466. https://doi.org/10.1080/13607860600638581

Lang, J. E., Moore, M. J., Harris, A. C., & Anderson, L. A. (2005). Healthy aging: Priorities and programs of the Centers for Disease Control and Prevention. Generations, 29 (2 ), 24-29.

Lang, F. R., Rieckmann, N., & Baltes, M. M. (2002). Adapting to aging losses: Do resources facilitate strategies of selection, compensation, and optimization in everyday functioning? Journals of Gerontology, Series B: Psychological Sciences and Social Sciences, 57(6), P501–P509. https://doi.org/10.1093/geronb/57.6.P501

Lapp, L. K., Agbokou, C., & Ferreri, F. (2011). PTSD in older individuals: The interaction between trauma and aging. International Psychogeriatrics, 1-11. https://doi.org/10.1017/S1041610211000366

Lawton, M. P., Kleban, M. H., Rajagopal, D., & Dean, J. (1992). Dimensions of affective experience in three age groups. Psychology and Aging, 7(2), 171–184. https://doi.org/10.1037/0882-7974.7.2.171

Lazarus, R. S., & Folkman, S. (1984). Stress, appraisal, and coping. Springer Publishing Company.

Lench, H. C., Bench, S. W., Darbor, K. E., & Moore, M. A. (2014). Functionalist manifesto: Goal-related emotions from an evolutionary perspective.

Levy, M., Deschênes, S. S., Burns, R. J., Elgendy, R., & Schmitz, N. (2019). Trajectories of social support in adults with type 2 diabetes: Associations with depressive symptoms and functional disability. International Journal of Geriatric Psychiatry, 34(3), 480–487. https://doi.org/10.1002/gps.4971

Li, W., Cheng, P., Liu, Z., Ma, C., Liu, B., Zheng, W., Scarisbrick, D., Lu, J., Li, L., Huang, Y., Wang, L., Yan, Y., Xiao, S., Zhang, Y., Zhang, T., Yan, J., Yu, Y., Xu, X., ... Liu, B. (2023). Post-traumatic stress disorder and traumatic events in China: A nationally representative cross-sectional epidemiological study. Psychiatry Research, 326, 115282. https://doi.org/10.1016/j.psychres.2023.115282

Lonnen, E., & Paskell, R. (2024). Gender, sex, and complex PTSD clinical presentation: A systematic review. European Journal of Psychotraumatology, 15(1), 2320994. https://doi.org/10.1080/20008066.2024.2320994

Louwers, M. A. M. (2015). Treatment of PTSD and comorbid symptoms in adult and senior citizens refugees: Does age matter? Clinical and Health Psychology, Faculty of Social Sciences, Utrecht University.

Lucanin, D. J., Lucanin, D., Bjelajac, A. K., Delale, A. D., & Stambuk, M. E. (2020). Life satisfaction determinants in older adults: Do different living arrangements count? Book of Selected Proceedings of the 21st Psychology Days in Zadar.

Lui, A., Glynn, S., & Shetty, V. (2009). The interplay of perceived social support and posttraumatic psychological distress following orofacial injury. Journal of Nervous and Mental Disease, 197(9), 639–645. https://doi.org/10.1097/NMD.0b013e3181b8d02d

Luong, G., Charles, S. T., & Fingerman, K. L. (2011). Better with age: Social relationships across adulthood. Journal of Social and Personal Relationships, 28(1), 9–23. https://doi.org/10.1177/0265407510391362

Maercker, A., Forstmeier, S., Wagner, B., Glaesmer, H., & Brähler, E. (2008). Posttraumatic stress disorder in Germany: The results of a nationwide epidemiological study. Der Nervenarzt, 79(5), 577-586. https://doi.org/10.1007/s00115-008-2467-5

Mainous, A. G., 3rd, Smith, D. W., Acierno, R., & Geesey, M. E. (2005). Differences in posttraumatic stress disorder symptoms between senior citizens non-Hispanic Whites and African Americans. Journal of the National Medical Association, 97(4), 546-549.

Recovered reference

Martins, B., Florjanczyk, J., Jackson, N. J., Gatz, M., & Mather, M. (2018). Age differences in emotion regulation effort: Pupil response distinguishes reappraisal and distraction for older but not younger adults. Psychology and Aging, 33(2), 338–349. https://doi.org/10.1037/pag0000227

Recovered reference

Mather, M., & Knight, M. (2005). Goal-directed memory: The role of cognitive control in older adults' emotional memory. Psychology and Aging, 20(4), 554–570. https://doi.org/10.1037/0882-7974.20.4.554

**Mbamalu, S. (2019, October 2).** Nigeria has a mental health problem. Al Jazeera. https://www.aljazeera.com

McCanlies, E. C., Mnatsakanov, A., Andrew, M. E., Burchfiel, C. M., & Violanti, J. M. (2014). Positive psychological factors are associated with lower PTSD symptoms among police officers post-Hurricane Katrina. Stress and Health, 30(5), 405–415. https://doi.org/10.1002/smi.2584

McDougall, M. A., Walsh, M., Wattier, K., Knigge, R., Miller, L., Stevermer, M., & Fogas, B. S. (2016). The effect of social networking sites on the relationship between perceived social support and depression. Psychiatry Research, 246, 223229https://doi.org/10.1016/j.psychres.2016.09.018

Missing reference

McFarlane, A. C. (2000). Posttraumatic stress disorder: A model of the longitudinal course and the role of risk factors. Journal of Clinical Psychiatry, 61(Suppl 5), 15–20; discussion 21–23.

Melkam, M., Tinsae, T., Andualem, F., & Nakie, G. (2023). Post-traumatic stress disorder and associated factors among adults exposed to stress in Ethiopia: A meta-analysis and systematic review. SAGE Open Medicine, 11, 1–10. https://doi.org/10.1177/20503121231160884

Mishara, W. L., & Gbaden, E. A. (2014). The prevalence of depression among the youths as an aftermath of the internal insurgency attacks in Maiduguri, Nigeria. *IOSR Journal of Humanities and Social Science (IOSR-JHSS)*, 19(10), 32–35. http://www.iosrjournals.org/iosr-jhss/papers/Vol19-issue10/Version-7/F0191073235.pdf https://doi.org/10.9790/0837-191073235

Mobolaji JW (2024) Multidimensional Deprivations and Associated Factors Among Older Adults in Urban Geographies of Nigeria: Implications for Poor Health Outcomes in Later Life. Int J Public Health 69:1606572. doi: 10.3389/ijph.2024.1606572

Mohammed, S. D., Garba, S., Sani, A. A., Ibrahim, R. T., Muhammad, Y. M., & Kassim, I. A. (2024). Pay As You Earn (PAYE) tax as panacea to backlog of gratuities and pensions of civil servants in Nigeria. Research Gate Preprint. https://doi.org/10.21203/rs.3.rs-4359446/v1

Mokuolu, O. B., & Yarseah, D. A. (2013). Assessment of post-traumatic stress and its pre-traumatic factors among Liberian refugees in Nigeria. IOSR Journal of Humanities and Social Science (IOSR-JHSS), 9(4), 1-5. https://doi.org/10.9790/0837-0940105

Missed reference

Moody, H. R. (2001). Productive aging and the ideology of old age. In N. Morrow-Howell, J. Hinterlong & M. Sherraden (Eds.), Productive aging: Concepts and challenges (pp. 176-196). Baltimore: The Johns Hopkins University Press

Moore, S. A. (2008). Cognitive abnormalities in posttraumatic stress disorder. *Current Opinion in Psychiatry*, 22, 19–24.

Mossakowski, K. N. (2014). Social causation and social selection. In W. C. Cockerham, R. Dingwall, & S. R. Quah (Eds.), *The Wiley Blackwell encyclopedia of health, illness, behavior, and society* (pp. 1-5). John Wiley & Sons, Ltd.

Mroczek, D. K., & Kolarz, C. M. (1998). The effect of age on positive and negative affect: A developmental perspective on happiness. *Journal of Personality and Social Psychology*, 75(5), 1333–1349. https://doi.org/10.1037/0022-3514.75.5.1333

National Population Commission. (2022).

Mugisha, J., Muyinda, H., Wandiembe, P., & Kinyanda, E. (2015). Prevalence and factors associated with posttraumatic stress disorder seven years after the conflict in three districts in northern Uganda (The Wayo-Nero Study). BMC Psychiatry, 15, 170. https://doi.org/10.1186/s12888-015-0551-5

Naito R, McKee M, Leong D, Bangdiwala S, Rangarajan S, Islam S, et al. (2023) Social isolation as a risk factor for all-cause mortality: Systematic review and meta-analysis of cohort studies. PLoS ONE 18(1): e0280308. https://doi. org/10.1371/journal.pone.0280308

Missing reference

National Bureau of Statistics. Retrieved from https://www.nigerianstat.gov.ng/elibrary/read/1123

**National Institute of Mental Health (NIMH).** (2023). NIMH website. https://www.nimh.nih.gov

**National Population Commission of Nigeria. (2020).** Nigeria living standards survey 2020.

National Bureau of Statistics. Retrieved from https://www.nigerianstat.gov.ng/elibrary/read/1123

Naveed, S., Waqas, A., Kumar, S., Abba, N., et al. (2020). Prevalence of common mental disorders in South Asia: A systematic review and meta-regression analysis. *Frontiers in Psychiatry*, 11, Article 573150. https://doi.org/10.3389/fpsyt.2020.573150

Nduanya, C. U., Odinka, J. I., Ndukuba, A. C., Okonkwo, O. A., Amadi, K. U., Iyidobi, T. O., Muomah, R. C., Oraegbunam, C. C., & Odinka, P. C. (2022). Pattern of psychiatric morbidity and somatic symptoms in a family medicine clinic of a university teaching hospital in Enugu, Southeast Nigeria. International Journal of Science and Research Archive, 6(1), 244–256. https://doi.org/10.30574/ijsra.2022.6.1.0129

Missing references

Neugarten, B. L. (1974). Age groups in American society and the rise of the young-old. Annals of the American Academy of Political and Social Science, 415(1), 187–198. https://doi.org/10.1177/000271627441500114

Neugarten, B. L., Havighurst, R. I., & Tobin, S. S. (1961). The measurement of life satisfaction. *Journal of Gerontology*.

Ng, L. C., Stevenson, A., Kalapurakel, S. S., Hanlon, C., et al. (2020). National and regional prevalence of posttraumatic stress disorder in sub-Saharan Africa: A systematic review and meta-analysis. *Frontiers in Public Health*.

Nichols, B., & Czirr, R. (1986). Post-traumatic stress disorder: Hidden syndrome in elders. Clinical Gerontologist, 5(3), 417–433. https://doi.org/10.1300/J018v05n03_07

Nikitin, J., & Freund, A. M. (2019). The process of aging. In R. Fernandez-Ballesteros, J. M. Robine, & A. Benetos (Eds.), *The Cambridge handbook of successful aging* (pp. 281–298). Cambridge University Press.

Norris, F., & Kaniasty, K. (1996). Received and perceived social support in times of stress: A test of the social support deterioration deterrence model. *Journal of Personality and Social Psychology*, 71(3), 498–511.

Norris, F. H. (1992). Epidemiology of trauma: Frequency and impact of different potentially traumatic events on different demographic groups. *Journal of Consulting and Clinical Psychology*, 60(3), 409–418. https://doi.org/10.1037/0022-006X.60.3.409

Odetol, T., Ankintayo-Usman, Okrie, A., Afolabe, T., & Ouwatosin, A. (2020). Life satisfaction assessment of senior citizens living in geriatric homes: Case study of a geriatric home in Ibadan, Nigeria. *African Journal of Biomedical Research*, 23(3), 335–342.

O'Donovan, A., Epel, E., Lin, J., Wolkowitz, O., Cohen, B., Maguen, S., & Neylan, T. C. (2011). Childhood trauma associated with short leukocyte telomere length in posttraumatic stress disorder. *Biological Psychiatry*, 70(5), 465–471. https://doi.org/10.1016/j.biopsych.2011.01.035

Ogle, C. M., Rubin, D. C., & Siegler, I. C. (2013a). Cumulative exposure to traumatic events in older adults. *Aging & Mental Health*, 18(3), 316–325. https://doi.org/10.1080/13607863.2013.841221

Ojagbemi, A., Bello, T., & Gureje, O. (2018). Gender differential in social and economic predictors of incident major depressive disorder in the Ibadan Study of Aging. *Social Psychiatry and Psychiatric Epidemiology*, 53(4), 351–361. https://doi.org/10.1007/s00127-018-1520-3

Ojeahere, M. I., Uwakwe, R., Piwunaa, C. G., Audua, M., et al. (2021). Assessment of full and subsyndromal PTSD and quality of life of internally displaced older adults in northern Nigeria. *Aging and Health Research*, 100040. https://doi.org/10.1016/j.ahr.2021.100040

Updated references

Okafor, C. J., Edet, B., & Asibong, U. (2015). Somatization symptoms in a primary care clinic at a tertiary hospital in southern Nigeria. Journal of Biosciences and Medicines, 3(10), 67. https://doi.org/10.4236/jbm.2015.310009

**Okoye, U., Ebimgbo, S., & Eneh, J. (2017).** Social work with older adults. In U. Okoye, N. Chukwu, & P. Agwu (Eds.), Social work in Nigeria: Book of readings (pp. 160–171). University of Nigeria Press Ltd.

Olugbile, O., Coker, M. P., Zachariah, Coker, O., Kuvinu, O., & Isichei, B. (2008). Provision of mental health services in Nigeria: Lagos State University Teaching Hospital, Ikeja, Lagos State, Nigeria. *International Psychiatry*, 5(2), 32.

Missed reference

**Onalu, C., & Nwafor, N. (Year).** Social supports available to persons with disabilities in Nigeria. In Evolutionary Psychology Meets Social Neuroscience. IntechOpen. https://doi.org/10.5772/intechopen.97790

Replaced

**Onyedika-Ugoeze, N. (2018, December 4).** Mental ill-health in Nigeria. The Guardian (Nigeria). Retrieved from https://www.irb-cisr.gc.ca/en/country-information/rir/Pages/index.aspx

Ouwehand, C., de Ridder, D. T., & Bensing, J. M. (2007). A review of successful aging models: Proposing proactive coping as an important additional strategy. *Clinical Psychology Review*, 27(8), 873–884. https://doi.org/10.1016/j.cpr.2007.04.002

Owens, G. P., Baker, D. G., Kasckow, J., Ciesla, J. A., & Mohamed, S. (2005). Review of assessment and treatment of PTSD among senior citizens. American armed forces veterans. International Journal of Geriatric Psychiatry, 20(12), 1118–1130. https://doi.org/10.1002/gps.1408

Owens, G. P., Steger, M. F., Whitesell, A. A., & Herrera, C. J. (2009). Posttraumatic stress disorder, guilt, depression, and meaning in life among military veterans. *Journal of Traumatic Stress*, 22(6), 654–657. https://doi.org/10.1002/jts.20456

Ozer, E. J., Best, S. R., Lipsey, T. L., & Weiss, D. S. (2003). Predictors of posttraumatic stress disorder and symptoms in adults: A meta-analysis. *Psychological Bulletin*, 129(1), 52–73. https://doi.org/10.1037/0033-2909.129.1.52

Missing reference

Peeters, M. C. W., & Le Blanc, P. M. (2001). Towards a match between job demands and sources of social support: A study among oncology care providers. European Journal of Work and Organizational

**Philipp, M. C. (2012).** The influence of social support on the relationship between individual differences and well-being. Journal of Individual Differences, 33(1), 1–9. https://doi.org/10.1027/1614-0001/a000059

Pierce, G. R., Lakey, B., Sarason, I. G., Sarason, B., & Joseph, H. J. (1997). Personality and social support processes: A conceptual overview. In G. R. Pierce, B. Lakey, I. G. Sarason, & B. Sarason (Eds.), *Sourcebook of social support and personality* (pp. 3-18). New York, NY: Plenum Press.

Pietrzak RH, Goldstein RB, Southwick SM, & Grant BF (2011). Prevalence and axis I comorbidity of full and partial posttraumatic stress disorder in the United States: Results from wave 2 of the national epidemiologic survey on alcohol and related conditions. Journal of Anxiety Disorders, 25(3), 456–465. doi:10.1016/j.janxdis.2010.11.010 [PubMed: 21168991]

Pietrzak, R. H., Goldstein, R. B., Southwick, S. M., & Grant, B. F. (2012b). Psychiatric comorbidity of full and partial posttraumatic stress disorder among older adults in the United States: Results from wave 2 of the national epidemiologic survey on alcohol and related conditions. *The American Journal of Geriatric Psychiatry*, 20(5), 380–390. https://doi.org/10.1097/JGP.0b013e31820d92e7

Prakash, O., & Kukreti, P. (2013). State of geriatric mental health in India. *Current Translational Geriatrics and Experimental Gerontology Reports*, 2(1), 6. https://doi.org/10.1007/s13670-013-0028-4

Prakash, S., & Srivastava, A. S. (2020). Perceived social support and life satisfaction among elderly people living separately from their adult children in the community: A cross-sectional comparative study. *Indian Journal of Gerontology*, 34(3), 281–292.

Proctor, C. L., Linley, P. A., & Maltby, J. (2009). Youth life satisfaction: A review of the literature. *Journal of Happiness Studies, 10*(5), 583–630. https://doi.org/10.1007/s10902-008-9110-9

PTSD UK. (2023). Post Traumatic Stress Disorder stats and figures. https://www.ptsduk.org/ptsd-stats/

Qureshi, S., Pyne, J., Magruder, K., Schulz, P., & Kunik, M. (2009). The link between posttraumatic stress disorder and physical comorbidities: A systematic review. *Psychiatric Quarterly, 80*(2), 87–97. https://doi.org/10.1007/s11126-009-9096-4

Riediger, M., Li, S. C., & Lindenberger, U. (2006). Selection, optimization, and compensation as developmental mechanisms of adaptive resource allocation: Review and preview. In J. E. Birren & K. W. Schaie (Eds.), *Handbook of the psychology of aging* (pp. 298–313). Academic Press. https://doi.org/10.1016/B978-012101264-9/50016-1

Robinson, S., Netanel, R., & Rapaport, J. (1992). Reactions of Holocaust survivors to the Gulf War and Scud missile attacks on Israel. In S. Robinson (Ed.), *Echoes of the Holocaust* (Vol. 1, pp. 1–12). Talbieh Mental Health Center.

Missing reference

Rohr, M.K., Lang, F.R., 2009. Aging well together: a mini-review. Gerontology 55, 333–343

Russell, D. W., & Cutrona, C. E. (1991). Social support, stress, and depressive symptoms among older individuals: Test of a process model. *Psychology and Aging, 6*(2), 190–201. https://doi.org/10.1037/0882-7974.6.2.190

Ryan, G. K., Nwefoh, E., Aguocha, C., Ode, P. O., Ocheche, P., et al. (2020). Partnership for the implementation of mental health policy in Nigeria: A case study of the Comprehensive Community Mental Health Programme in Benue State. *International Journal of Mental Health Systems*. https://doi.org/10.1186/s13033-020-00344-z

Şahin, D. S., Özer, Ö., & Yanardağ, M. Z. (2019). Perceived social support, quality of life, and satisfaction with life in senior citizens. *Educational Gerontology, 45*, 69–77. https://doi.org/10.1080/03601277.2019.1585065

Saleem, J., Begum, R., & Naheem, M. (2022). Impacts of depressive symptoms on life satisfaction among senior citizen women of Bahawalpur, Pakistan. *Pakistan Journal of Social Research, 4*(2), 666–674.

Samantaray, N. N., & Kar, N. (2021). Improving depression and well-being in older adults using selection, optimization, and compensation model: A case series article. *Journal of Geriatric Mental Health*. https://doi.org/10.4103/jgmh.jgmh_7_21

Samantaray, N. N., & Kay, N. (2021). Improving depression in older adults: Using selection optimization and compensation. *Journal of Geriatric Mental Health, 8*(10).

Samaranayake, C. B., & Fernando, A. T. (2011). Satisfaction with life and depression among medical students in Auckland, New Zealand. *The New Zealand Medical Journal, 124*(1341), 12–17.

**Sasu, D. D. (2022).** Poverty headcount rate in Nigeria 2022, by state. Statista. Retrieved from https://www.statista.com/statistics/1234383/poverty-headcount-rate-in-nigeria-by-state/

**Missing reference**

Schaie, K. W. (2016). Theoretical perspectives for the psychology of aging in a lifespan context. In K. W. Schaie & S. L. Willis (Eds.), Handbook of the psychology of aging (8th ed., pp. 3–13). Elsevier.

Schmidt, U., Holsboer, F., & Rein, T. (2011). Epigenetic aspects of posttraumatic stress disorder. [10.3233/DMA-2011-0749]. Disease Markers, 30(2), 77-87.

Schuitevoerder, S., Rosen, J. W., Twamley, E. W., Ayers, C. R., Sones, H., Lohr, J. B., Goetter, E. M., Fonzo, G. A., Holloway, K. J., & Thorpa, S. R. (2013). A meta-analysis of cognitive functioning in older adults with PTSD.J Anxiety Disord 27:550–558

Missing reference

Schulz, U., & Schwarzer, R. (2004). Long-term effects of spousal support on coping with cancer after surgery. Journal of Social and Clinical Psychology, 23(5), 716-732.

Sekonil, O., Mall, S., & Christofides, N. (2021). Prevalence and factors associated with PTSD among female urban slum dwellers in Ibadan, Nigeria: A cross-sectional study. *BMC Public Health, 21*, 1546. https://doi.org/10.1186/s12889-021-11508-y

Sheikh, T. L., Mohammed, A., Eseigbe, E., Adekeye, T., Nuhu, F. T., Lasisi, M., et al. (2016). Descriptive characterization of psycho-trauma, psychological distress, and posttraumatic stress disorder among children and adolescent internally displaced persons in Kaduna, Nigeria. *Frontiers in Psychiatry, 7*, 179. https://doi.org/10.3389/fpsyt.2016.00179

Shensa, A., Sidani, J. E., Escobar-Viera, C. G., Switzer, G. E., Primack, B. A., & Choukas-Bradley, S. (2020). Emotional support from social media and face-to-face relationships: Associations with depression risk among young adults. *Journal of Affective Disorders, 260*, 38–44. https://doi.org/10.1016/j.jad.2019.08.092

Missing reference

Shirzadifard, M., Shahghasemi, E., & Hejazi, E. (2020). Life management strategies as mediators between information processing style and subjective well-being. SAGE Open, 10(4), 1–10. https://doi.org/10.1177/2158244020962806

**Shock, N. (2025). Blood tests suggest that social disadvantage can accelerate aging and increase disease risk.** ( March 14). Medical Xpress. Retrieved from https://www.medicalxpress.com/

Siedlecki, K. L., Salthouse, T. A., Oishi, S., & Jeswani, S. (2014). The relationship between social support and subjective well-being across age. *Social Indicators Research, 117*, 561–576. https://doi.org/10.1007/s11205-013-0361-4

Simon, N., Roberts, N. P., Lewis, M. C. E., Gelderen, J. V., & Bisson, J. (2019). Associations between perceived social support, posttraumatic stress disorder (PTSD), and complex PTSD (CPTSD): Implications for treatment. *European Journal of Psychotraumatology, 10*, 1573129. https://doi.org/10.1080/20008198.2019.1573129

Sledjeski, E. M., Speisman, B., & Dierker, L. (2008). Does the number of lifetime traumas explain the relationship between PTSD and chronic medical conditions? Answers from the National Comorbidity Survey-Replication (NCS-R). *Journal of Behavioral Medicine, 31*(4), 341–349. https://doi.org/10.1007/s10865-008-9158-3

Missing data

Song, J., & Fan, H. (2013). A meta-analysis of the relationship between social support and subjective well-being. Advances in Psychological Science, 21(8), 1357-1370. https://doi.org/10.3724/SP.J.1042.2013.01357

Spitzer, C., Barnow, S., Volzke, H., John, U., Freyberger, H. J., & Grabe, H. J. (2008). Trauma and posttraumatic stress disorder in older people: Findings from a German community study. *Journal of Clinical Psychiatry, 69*(5), 693–700. https://doi.org/10.4088/JCP.v69n0514

**Stawski, R. S., Sliwinski, M. J., Almeida, D. M., & Smyth, J. M. (2008).** Reported exposure and emotional reactivity to daily stressors: The roles of adult age and global perceived stress. Psychology and Aging, 23(1), 52–61. https://doi.org/10.1037/0882-7974.23.1.52

Stonewall. (2023). List of LGBTQ+ terms. https://www. stonewall.org.uk/list-lgbtq-terms

Stovall-McClough, K. C., & Cloitre, M. (2006). Unresolved attachment, PTSD, and dissociation in women with childhood abuse histories. *Journal of Consulting and Clinical Psychology, 74*(2), 219–228. https://doi.org/10.1037/0022-006X.74.2.219

Stuber, J., Resnick, H., & Galea, S. (2006). Gender disparities in posttraumatic stress disorder after mass trauma. *Gender Medicine, 3*, 54–67. https://doi.org/10.1016/j.genm.2006.04.004

Missing reference

Summerfield, D. (2004). Cross-cultural perspectives on the medicalization of human suffering. In G.M.

Rosen (Ed.), Posttraumatic stress disorder: Issues and controversies (p. 233-245). West Sussex,

England: John Wiley & Sons

Swickert, R. (2009). Personality and social support. In P. Corr & G. Matthews (Eds.), *Cambridge handbook of personality* (pp. 524–540). Cambridge University Press.

Tade, O. (2020). Conflict between herders and farmers: Nigeria needs to accept there are victims on both sides. *University of Ibadan*.

Teshale, S. M., & Lachman, M. E. (2016). Managing daily happiness: The relationship between selection, optimization, and compensation strategies and well-being in adulthood. *Psychology and Aging, 31*(7), 687–692. https://doi.org/10.1037/pag0000132

Tesfaye AH, Sendekie AK, Kabito GG, Engdaw GT, Argaw GS, Desye B, et al. (2024) Posttraumatic stress disorder and associated factors among internally displaced persons in Africa: A systematic review and meta-analysis. PLoS ONE 19(4): e0300894. https://doi.org/10.1371/journal. pone.0300894

THISDAYLIVE. (2019, September 5). Nigeria’s healthcare budget falls short of AU target. Retrieved from https://www.thisdaylive.com

**Thoits, P. A. (1986).** Social support as coping assistance. Journal of Health and Social Behavior, 27(1), 77–89. https://doi.org/10.2307/2136504

**The Guardian [Nigeria].** (2020, January 13). Psychiatrists Harp on Passage of Mental Health Bill. Retrieved October 16, 2020, from [Insert URL if available]

Thomas, S., Kanske, P., Schaf, J., Hummel, K. V., & Trautmann, S. (2022). Examining bidirectional associations between perceived social support and psychological symptoms in the context of stressful event exposure: A prospective, longitudinal study. *BMC Psychiatry, 22*(1), 736. https://doi.org/10.1186/s12888-022-04386-0

Missing reference

Thompson, M. P., Norris, F. H. and Hanacek, B. (1993). Age differences in the psychological consequences of Hurricane Hugo. Psychology And Aging, 8, 606–616.

Thoresen, S., Birkeland, M. S., Arnberg, F. K., Wentzel-Larsen, T., & Blix, I. (2019). Long-term mental health and social support in victims of disaster: Comparison with a general population sample. *BJPsych Open, 5*(1), e2.

Togonu-Bickersteth, F., & Akinyemi, A. I. (2014). Ageing and national development in Nigeria: Costly assumptions and challenges for the future. *African Population Studies, 27*(2 Suppl).

Missing references

**Uchino, B. N. (2004).** Social support and physical health: Understanding the health consequences of relationships. Yale University Press.

**Uchino, B. N. (2009).** Understanding the links between social support and physical health: A life-span perspective with emphasis on the separability of perceived and received support. Perspectives on Psychological Science, 4(3), 236-255. https://doi.org/10.1111/j.1745-6924.2009.01122.x

Uchino BN, Carlisle MC, Birmingham W, Vaughn AA. Social Support and the Reactivity Hypothesis: Conceptual Issues in Examining the Efficacy of Received Support During Acute Psychological Stress. Biological Psychology. 2011; 86:137–142.10.1016/j.biopsycho.2010.04.003 [PubMed: 20398724]

Ullman, S. E., & Filipas, H. H. (2001). Predictors of PTSD symptom severity and social reactions in sexual assault victims. *Journal of Traumatic Stress, 14*(2), 369–389. https://doi.org/10.1023/a:1011125220522

United Nations (UN). (2018). World Health Organization (WHO). *Nigeria: Mental Health Atlas 2017 Member State Profile.* [Accessed 6 Oct. 2020].

Van der Wal, S. J., Vermetten, E., & Eric, G. (2020). Long-term development of posttraumatic stress symptoms and associated risk factors in military service members deployed to Afghanistan: Results from the PRISMO 10-year follow-up. *European Psychiatry, 64*(1), e10, 1–9. https://doi.org/10.1192/j.eurpsy.2020.113

Vanguard News. (2021, August 26). Nigeria’s health sector and budgetary allocation challenges. Retrieved from https://www.vanguardngr.com

Wagner, A. C., Monson, C. M., & Hart, T. L. (2016). Understanding social factors in the context of trauma: Implications for measurement and intervention. *Journal of Aggression, Maltreatment & Trauma, 25*(8), 831–853.

Wang, Y., Chung, M. C., Wang, N., Yu, X., & Kenardy, J. (2021). Social support and posttraumatic stress disorder: A meta-analysis of longitudinal studies. *Clinical Psychology Review, 85*, 101998.

Ward, R. A. (1985). Informal networks and well-being in later life: A research agenda. *Gerontologist, 25*(1), 55–61.

Weiland, M., Dammermann, C., & Stoppe, G. (2011). Selective optimization with compensation (SOC) competencies in depression. *Journal of Affective Disorders, 133*, 114–119.

West, S. G., Finch, J. F., & Curran, P. J. (1995). Structural equation models with nonnormal variables: Problems and remedies. In R. H. Hoyle (Ed.), Structural equation modeling: Concepts, issues, and applications (pp. 56–75). Sage Publications, Inc.

Missing references

Wethington, E., & Kessler, R. C. (1986). Perceived support, received support, and adjustment to stressful life events. Journal of Health and Social Behavior, 27(1), 78–89.

Whitbourne, S. K., & Sneed, J. R. (2002). The paradox of well-being, identity processes, and stereotype threat: Ageism and its potential relationships to the self in later life. In T. D. Nelson (Ed.), Ageism: Stereotyping and prejudice against older persons (pp. 247–273). The MIT Press

Wiese, B. S., Freund, A. M., & Baltes, P. B. (2000). Selection, optimization, and compensation: An action-related approach to work and partnership. *Journal of Vocational Behavior, 57*, 273–300. https://doi.org/10.1006/jvbe.2000.1752

Wiese, B. S., & Freund, A. M. (2000). The interplay of work and family in young and middle adulthood. In J. Heckhausen (Ed.), *Motivational psychology of human development: Developing motivation and motivating development* (pp. 233–249). Elsevier.

**Williams, R., & Joseph, S. (1999).** Conclusions: An integrative psychosocial model of PTSD. In W. Yule (Ed.), Posttraumatic stress disorders: Concepts and therapy (pp. 297–314). Wiley.

Wisco, B. E., Marx, B. P., & Keane, T. M. (2012). Screening, diagnosis, and treatment of post-traumatic stress disorder. *Military Medicine, 177*(7), 7–13.

Woodward, M. J., Eddinger, J., Henschel, A. V., Dodson, T. S., Tran, H. N., & Beck, J. G. (2015). Social support, posttraumatic cognitions, and PTSD: The influence of family, friends, and a close other in an interpersonal and noninterpersonal trauma group. *Journal of Anxiety Disorders, 35*, 60–67. https://doi.org/10.1016/j.janxdis.2015.09.002

Woodward, M. J., Morissette, S. B., Kimbrel, N. A., Meyer, E. C., DeBeer, B. B., Gulliver, S.

B., & Beck, G. J. (2018). A cross-lagged panel approach to understanding social support and chronic posttraumatic stress disorder symptoms in veterans: Assessment modality matters. *Behavior Therapy, 49*(5), 796–808.

World Bank. (2019). Fragile and conflict-affected situations: World Bank Group. Retrieved July 11, 2019, from https://data.worldbank.org/region/fragile-and-conflict-affected-situations.

World Health Organization (2013). *Global and regional estimates of violence against women: Prevalence and health effects of intimate partner violence and non-partner sexual violence*. Geneva.

World Health Organization (2008). mhGAP Mental Health Gap Action Programme. Available from http://www.who.int/mental_health/evidence/mhGAP/en/index.html.

World Health Organization. (2023). PTSD. Creative Commons Attribution-NonCommercial-ShareAlike 3.0 IGO. https://creativecommons.org/licenses/by-nc-sa/3.0/igo

Wu, N., Ding, F., Zhang, R., Cai, Y., & Zhang, H. (2022). The relationship between perceived social support and life satisfaction: The chain mediating effect of resilience and depression among Chinese medical staff. *Journal of Environmental Research and Public Health, 9*(24), 16646.

Yarseah, A. D. (2017). *Posttraumatic stress disorder among Liberian refugees in Nigeria* (Master's thesis).

**Yarseah, D. A., Ogunsanmi, J. O., Ibimiluyi, O. F., Olaoye, E. O., Ezeani, E. S., & Ogunsanmi, O. O. (2023).** The mediating effects of perceived social support and shame on psychological distress and their dimensions among Liberian refugees in Nigeria [Preprint]. Research Square. https://doi.org/10.21203/rs.3.rs-3243472/v1

Young, L. M., Baltes, B. B., & Pratt, A. K. (2007). Using selection, optimization, and compensation to reduce job/family stressors: Effective when it matters. *Journal of Business and Psychology, 21*, 511–539.

Yuen, H. K., & Vogtle, L. K. (2016). Multimorbidity, disability, and adaptation strategies among community-dwelling adults aged 75 years and older. *Disability and Health Journal, 9*, 593–599. https://doi.org/10.1159/000189213

Yunitri, N., Chu, H., Xiao, L. K., Jen, H., et al. (2022). Global prevalence and associated risk factors for posttraumatic stress disorder during the COVID-19 pandemic: A meta-analysis. *International Journal of Nursing Studies*. https://doi.org/10.1016/j.ijnurstu.2021.104136

Yuvakgil, Z., & Akyil, R. C. (2021). Assessing perceived social support, social network, and healthy lifestyle behaviors in older adults. *International Journal of Caring Sciences, 14*(2), 1274.

Zając-Lamparska, L. (2021). Selection, optimization, and compensation strategies and their relationship with well-being and impulsivity in early, middle, and late adulthood in a Polish sample. *BMC Psychology*.

Zeiss, R., Dickman, H., & Nichols, B. (1985). Posttraumatic stress disorder in former prisoners of war. Paper presented at the annual convention of the American Psychological Association, Los Angeles, CA.

Zelst, W. H. V., de Beurs, E., Beekman, A. D. F., Deeg, D. J. H., & Dyc, D. V. (2017). Prevalence and risk factors of posttraumatic stress disorder in older adults. *Journal of Psychotherapy and Psychometrics, 72*, 333–342.

Zlotnick, C., Johnson, J., Kohn, R., & Viente, B. (2006). Epidemiology of trauma, posttraumatic stress disorder (PTSD), and comorbid disorders in Chile. *PubMed*. https://doi.org/10.1017/S0033291706008282

Zuroff, D.C., Blatt, S.J., Sanislow, C.A.I., Bondi, C.M., Pilkonis, P.A., 1999. Vulnerability to depression: reexamining state dependence and relative stability. J. Abnorm. Psychol. 108 (1), 76–89.
